# Genome-wide transcript expression analysis reveals major chickpea and lentil genes associated with plant branching

**DOI:** 10.3389/fpls.2024.1384237

**Published:** 2024-06-19

**Authors:** Marcos Fernando Basso, Giacomo Girardin, Chiara Vergata, Matteo Buti, Federico Martinelli

**Affiliations:** ^1^ Department of Biology, University of Florence, Florence, Italy; ^2^ Department of Agriculture, Food, Environment and Forestry (DAGRI), University of Florence, Florence, Italy

**Keywords:** legume, biotechnological tool, branching, plant architecture, pulse, RNA-Seq, transcription factor

## Abstract

The search for elite cultivars with better architecture has been a demand by farmers of the chickpea and lentil crops, which aims to systematize their mechanized planting and harvesting on a large scale. Therefore, the identification of genes associated with the regulation of the branching and architecture of these plants has currently gained great importance. Herein, this work aimed to gain insight into transcriptomic changes of two contrasting chickpea and lentil cultivars in terms of branching pattern (little *versus* highly branched cultivars). In addition, we aimed to identify candidate genes involved in the regulation of shoot branching that could be used as future targets for molecular breeding. The axillary and apical buds of chickpea cultivars Blanco lechoso and FLIP07–318C, and lentil cultivars Castellana and Campisi, considered as little and highly branched, respectively, were harvested. A total of 1,624 and 2,512 transcripts were identified as differentially expressed among different tissues and contrasting cultivars of chickpea and lentil, respectively. Several gene categories were significantly modulated such as cell cycle, DNA transcription, energy metabolism, hormonal biosynthesis and signaling, proteolysis, and vegetative development between apical and axillary tissues and contrasting cultivars of chickpea and lentil. Based on differential expression and branching-associated biological function, ten chickpea genes and seven lentil genes were considered the main players involved in differentially regulating the plant branching between contrasting cultivars. These collective data putatively revealed the general mechanism and high-effect genes associated with the regulation of branching in chickpea and lentil, which are potential targets for manipulation through genome editing and transgenesis aiming to improve plant architecture.

## Introduction

Chickpea (*Cicer arietinum* L.) and lentil (*Lens culinaris* Medik.) are remarkable pulse crops (*Fabaceae* family) of outstanding importance for human consumption as sources of vegetable proteins for several European and Asian countries (Landi et al., 2021; Karalija et al., 2022). The chickpea is a self-pollinated diploid, annual-perennial, and dicotyledon, semi-erect, with a genome size estimated in 738 Mb organized in sixteen chromosomes (2n = 2x = 16) and 28,200 annotated genes (Varshney et al., 2013). In turn, lentil is a self-pollinated diploid (2n = 2x = 14), annual, and dicotyledonous, semi-erect, with a genome size estimated in 3.69 Gb organized in fourteen chromosomes and 58,243 annotated genes (Ramsay et al., 2021). To date, several germplasm banks worldwide with a high number of accessions, genotypes, lines, and commercial cultivars are available for these crops. However, there is an enormous genotypic and phenotypic variability among these genetic materials, being that the majority of these cultivars have a high number of non-dominant lateral branching and few branches with dominant growth and erect stem (Cici et al., 2008; Singh et al., 2019; Liber et al., 2021). These intrinsic agronomic characteristics need to be improved since nowadays typical chickpea and lentil cultivars have a highly complex architecture for open-field management, making mechanical harvesting difficult and increasing lodging and susceptibility to biotic and abiotic stresses (Silva-Perez et al., 2022; Tripathi et al., 2022).

The increasing and severe climate change and demand for healthy food in sufficient quantity are major factors that are challenging agriculture and consumer populations around the world (Arif et al., 2021; Grossi-de-Sa and Basso, 2024; Basso et al., 2024b). Given this, it is urgent to spend breeding efforts to improve the agronomic traits of these crops associated with abiotic and biotic tolerance, grain yield, nutritional features, and plant architecture to produce more food at a lower cost per area (Weller and Ortega, 2015; Haile et al., 2021; Asati et al., 2022; Basso et al., 2023). In particular, a significant effort still needs to be made to develop more adapted cultivars to enhance the mechanization of planting and harvesting systems (Yang et al., 2021). Fortunately, for both these crops there is a huge amount of genetic variability in wild accessions and commercial cultivars in germplasm banks that can be explored using next-generation sequencing approaches (Piergiovanni, 2022). Therefore, understanding the molecular basis that contributes to the increased or reduced plant branching of these two crops is an important advance for developing these new cultivars with an architecture more suitable to mechanized harvesting (Sandhu and Singh, 2007; Koul et al., 2022; Beveridge et al., 2023). The identification of genes regulating branching architecture in both lentil and chickpea will allow to deliver of candidate targets for biotechnological breeding approaches such as new genome editing technologies and genetic engineering techniques (Basso et al., 2019, 2020). Although knowledge of the genetic basis associated with different agronomically important traits of these two crops has been explored in recent years, little is known about the molecular mechanisms involved in the branching and architecture of chickpea and lentil. A recent study identified and characterized the expression profile of *SMAX/SMXL* family genes in the chickpea and lentil revealing several strigolactones-associated genes with positive or negative correlations with the plant branching level (Basso et al., 2024a).

Herein, the global transcript expression profile in axillary and apical buds of contrasting cultivars of chickpea and lentil in terms of branching patterns (little and highly branched) was explored by RNA-seq. These collective data revealed several genes putatively associated with the regulation of branching in both chickpea and lentil. These genes are highlighted and discussed as targets for genetic manipulation through genome editing and transgenesis aiming to improve the plant architecture of chickpea and lentil.

## Materials and methods

### Plant material

In this study, two contrasting cultivars of chickpea and lentil were selected dealing with plant branching, according to a previous study carried out by Basso et al. (2024a). The chickpea cultivars Blanco lechoso and FLIP07–318C were used as little and highly branched, respectively. Likewise, lentil cultivars Castellana and Campisi were also used as little and highly branched, respectively. Seeds of the chickpea and lentil cultivars were superficially sterilized with 1.5% sodium hypochlorite solution for 1 minute, washed abundantly with distilled water, soaked for 3 minutes in distilled water, and germinated in Petri plate containing humid filter paper during three days at room temperature. The germinated seeds with a 1–2 cm radicle were transferred to pots containing commercial substrate and kept well-watered and fertilized under greenhouse conditions.

### Experimental design

For this study, the chickpea cultivars Blanco lechoso (little branched) and FLIP07–318C (highly branched), and lentil cultivars Castellana (little branched) and Campisi (highly branched) were selected based on a previous study where the architecture/branching of these four cultivars was characterized and, among several cultivars, these four were considered most contrasting for this phenotype (Basso et al., 2024a). The cultivars Blanco lechoso and Castellana are characterized by presenting a low number of lateral branches and a dominant, well-defined, and semi-erect stem (Scarrone-type plant architecture; Hallé and Oldeman, 1970). In contrast, the cultivars FLIP07–318C and Campisi are characterized by presenting a high number of lateral branches and the absence of a dominant, well-defined, and erect stem (Schoute-type plant architecture; Hallé and Oldeman, 1970). Axillary buds are the precursor of the branches and lateral shoots, while the apical buds regulate the apical dominance. For this reason, we analyzed both axillary and apical buds for each of the four genotypes. Physiological, hormonal, and transcriptional balance are considered the main factors that define the prevalence of axillary bud or apical bud growth in a given cultivar (Beveridge et al., 2023). This study focused on the identification of genes involved in plant branching using a transcriptomic approach. For this, plant material of chickpea and lentil contrasting cultivars, highly integrity RNA, libraries preparation, high-throughput cDNA sequencing, and RNA-seq raw data were successfully conducted and achieved.

### Construction and sequencing of RNA libraries

Axillary and apical buds were collected separately from at least 15 plants randomized per biological replicate after 20 days of transplanting and the samples were kept in liquid nitrogen. Frozen tissues (50–100 mg) were ground to a fine powder with a mortar and pestle using liquid nitrogen. The total RNA was purified with GenUP™ Total RNA Kit (Biotechrabbit, Volmerstraße, Berlin, Germany). The RNA integrity was checked through agarose electrophoresis, while the concentration of total RNA was measured using a Qubit 4 Fluorometer and Qubit kit (Invitrogen, Waltham, Massachusetts, USA). The purity and integrity of RNA were confirmed by the Agilent Bioanalyser 2100 system (RNA 6000 Nano Kit, Agilent Technologies, Santa Clara, CA, USA). Twenty-four sequencing libraries were prepared using Truseq Stranded mRNA Library Prep and Truseq RNA Single Indexes (Illumina, San Diego, CA, USA) following the manufacturer’s instructions. A unique dual index combination was used for each sample/library for barcoding. The concentration of each of the 24 libraries was determined using the Qubit 4 Fluorometer and the dsDNA High Sensitivity Kit (Invitrogen). All samples were sequenced using a NovaSeq 6000 platform (Illumina) and the Novaseq 6000 S1 Reagent Kit (2 x 100 + 10 + 10 bp parameters) following Illumina standard procedure in XP mode. All libraries were run in a single lane of the flow cell.

### RNA-seq data elaboration, and differential expression analyses

The RNA-seq raw data in.*fastq* format were obtained from BCL files using bcl2fastq2 v2.20 tool (Illumina). The quality assessment of the sequenced libraries was performed with FastQC v0.11.9 (Andrews, 2010). Adaptors and low-quality bases were removed using Trimmomatic PE v0.39 (Bolger et al., 2014). Filtered reads were aligned to the chickpea and lentil genome assemblies using the HiSat2 v2.2.1 tool (Kim et al., 2019). The reference genome used for chickpea data was the *C. arietinum* CDC Frontier genome ASM33114 assembly v1 (Varshney et al., 2013) while, for lentil data, the CDC Redberry genome v2.0 (Ramsay et al., 2021) was used. Read count was performed using the FeatureCounts v2.0.3 tool with default parameters (Liao et al., 2013) based on the reference transcripts predictions. Differential expression analyses were carried out using the Bioconductor EdgeR package v3.28.1 (Robinson et al., 2009). EdgeR was used to filter out unexpressed or poorly expressed transcripts, normalize the RNA libraries, and perform the differential expression analyses with the Likelihood-Ratio Test (LTR). A transcript was considered ‘active’ if the reads per million mapping to that transcript were >1 in at least two libraries. Transcripts with a false discovery rate (FDR) <0.05 and log(fold change) [the acronym of log2(fold change)] lower than -2 or greater than +2 were considered to be differentially expressed.

### Functional data mining and enrichment analyses

According to the differential expression analyses results, transcripts with the same expression trend (up- or down-regulation) were detected for the four pairwise comparisons: chickpea (*i*) Blanco lechoso axillary bud *versus* Blanco lechoso apical bud (BX x BA), (*ii*) FLIP07–318C axillary bud *versus* FLIP07–318C apical bud (FX x FA), (*iii*) FLIP07–318C axillary bud *versus* Blanco lechoso axillary bud (FX x BX), (*iv*) FLIP07–318C apical bud *versus* Blanco lechoso apical bud (FA x BA), lentil (*v*) Campisi axillary bud *versus* Campisi apical bud (CmX x CmA), (*vi*) Castellana axillary bud *versus* Castellana apical bud (CsX x CsA), (*vii*) Castellana axillary bud *versus* Campisi axillary bud (CsX x CmX), and (*viii*) Castellana apical bud *versus* Campisi apical bud (CsA x CmA). For each differentially expressed transcript in chickpea and lentil their corresponding orthologous genes were identified in *Arabidopsis thaliana* using BlastX against TAIR10 proteome with an *e-*value threshold of 10^-5^. The MapMan 3.6.0RC1 software was used with the available *A. thaliana* mapping file (https://mapman.gabipd.org/mapman) to identify and visualize genes in functional overviews of cell pathways and gene categories (Thimm et al., 2004). The transcript set enrichment analysis was carried out with the same list of differentially expressed transcripts using PageMan software (https://mapman.gabipd.org/pageman) (Usadel et al., 2006). The PageMan analysis was performed using the Wilcoxon test without correction and with a cutoff value = 1 (Wilcoxon, 1945). The DAVID database v.6.8 (Dennis et al., 2003) was used to obtain the gene ontology (GO) information related to each biological process. KEGG pathway enrichment analyses were carried out on differentially expressed transcript sets to identify relevant pathways enriched for each pairwise comparison. The KEGG pathway enrichment analyses were conducted with KOBAS-i web tool (Bu et al., 2021). While chickpea is a species supported by KOBAS-i, lentil is not, so DETs Arabidopsis orthologs were used for lentil’s enrichment analyses. The bubble diagrams were plotted with ggplot2 v3.4.3 R visualization package (Wickham, 2016). The chromosomal location of the chickpea and lentil genes was evidenced by the MapGene2Chrom program v2 (Jiangtao et al., 2015).

### Gene expression profile by real-time RT-PCR

The RNA samples purified as described above were treated with RNase-free RQ1 DNase I (Promega, Madison, Wisconsin, EUA) and used for cDNA synthesis using oligo-(dT)_20_ primer and SuperScript III RT mix (Life Technologies, Carlsbad, CA, USA). The cDNA samples were diluted 1:10 (v:v) with nuclease-free water, while the real-time RT-PCR assays were performed in QuantStudio 7 Flex Real-Time PCR platform (Applied Biosystems, Waltham, MA, USA) using 2.5 µL cDNA, 0.1 µM gene-specific primers (**Supplementary Table S1**), and SYBR Green PCR Master Mix (Applied Biosystems, Waltham, MA, USA). For validation of RNA-seq data, the *CaBES1*, *CaFHY3*, *CaFAR1*, *CaDOF4.2*, and *CaFHY1* genes were selected for evaluation in chickpea samples, while *LcFITNESS*, *LcFHY3*, *LcFAR1*, *LcDOF4.2*, and *LcBS1* genes were selected for lentil samples (**Supplementary Tables S1**, **S2**). The *CaCAC* (Reddy et al., 2016) and *LcTUB* (Sinha et al., 2019) were used as endogenous reference genes for normalization (**Supplementary Table S1**). The reference genes *CaG6PD* and *CaTIP41*, *LcRPL2*, and *LcRBC1* were also tested, but *CaCAC* and *LcTUB* were more stable in our preliminary test as a reduced number of samples. The relative gene expression, fold change, and log(fold change) were calculated with the 2^-ΔCt, 2^-ΔΔCt, and Log(fold change) formulas, respectively. Three biological replicates for each treatment and at least 15 plants for each biological replicate were used. All cDNA samples were carried out in technical triplicates. The target-specific amplification for each pair primer was confirmed by the occurrence of a single peak observed in the melting curve. To validate the transcriptional level obtained from RNA-seq datasets, the relative or normalized expression values (2^-ΔCt) obtained from real-time RT-PCR were correlated using the Pearson correlation coefficient to normalized expression values based on transcript per million (TPM) values obtained from RNA-seq for each of the five selected genes in each library or sample, for both chickpea and lentil.

## Results

### RNA-seq libraries construction, data elaboration, and differential expression analysis

**Figure 1:**
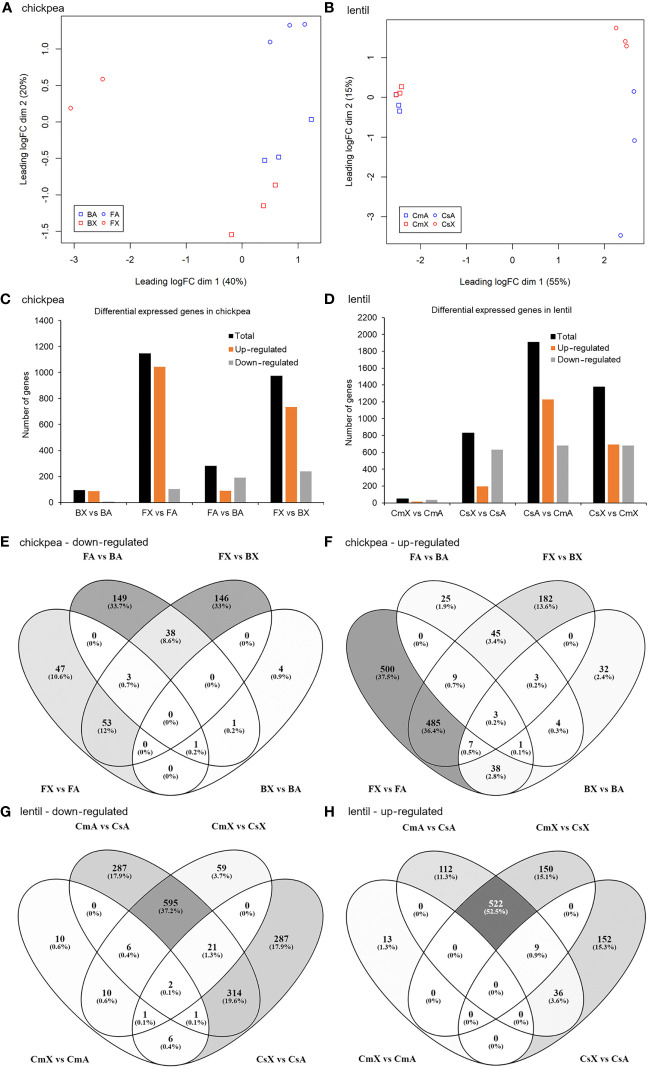
Correlation analysis among different samples of chickpea and lentil based on transcript expression values and number of differentially expressed transcripts in each pairwise comparison for both chickpea and lentil genotypes and tissues. MDS analysis of the 23 RNA-seq datasets for **(A)** chickpea and **(B)** lentil samples. Percentages represent variance captured by each principal component 1 and 2 in each analysis. Comparison between **(C)** chickpea cv. Blanco lechoso (B; little branched cultivar) and cv. FLIP07–318C (F; highly branched cultivar), **(D)** lentil cv. Castellana (Cs; little branched cultivar), and cv. Campisi (Cs; highly branched cultivar). BX: Blanco lechoso axillary bud, BA: Blanco lechoso apical bud, FX: FLIP07–318C axillary bud, FA: FLIP07–318C apical bud, CsX: Castellana axillary bud, CsA: Castellana apical bud, CmX: Campisi axillary bud, and CmA: Campisi apical bud. Only transcripts with FDR <0.05 and log(fold change) lower than -2 or greater than +2 were considered as differentially expressed transcripts. Venn diagrams of the overlapped differentially expressed transcripts by comparing the contrast between different genotypes and tissues of **(E, F)** chickpea and **(G, H)** lentil. The number and percentage of commonly and uniquely differentially expressed transcripts were indicated.

In total, 24 libraries were constructed and sequenced, 12 libraries for chickpea and 12 for lentil (two cultivars each x two tissues x three biological replicates). One library of the cultivar FLIP07–318C corresponding to the axillary bud sample was removed from subsequent bioinformatic analyses due to the reduced number of reads. The raw sequences of the RNA libraries were deposited on the EMBL-EBI ArrayExpress database (https://www.ebi.ac.uk/biostudies/arrayexpress) under the accession number E-MTAB-13679. Overall, taking together the RNA-seq raw reads generated from the 11 chickpea libraries, 90.79 to 93.59% of these paired reads passed quality control and filtering steps. In total, 3,091.832 to 13,316.821 filtered reads were obtained, of which 97.39 to 98.57% were mapped to the transcript dataset of the reference genome (**Supplementary Table S2**). In contrast, from RNA-seq raw reads generated from the 12 lentil libraries, 90.76 to 94.02% of these paired reads passed quality control and filtering steps, 5,862.483 to 12,410.646 filtered reads were obtained, of which 93.68 to 96.56% were mapped to the transcript dataset of the reference genome (**Supplementary Table S2**). The number of reads per library mapped to each of the chickpea and lentil reference transcripts was estimated and, among them, only 14,324 and 20,884, respectively, resulted as active transcripts, and were used for further analyses (**Supplementary Files S1**, **S2**). The 23 RNA libraries were normalized according to the amounts of filtered reads. Then, filtered and normalized counts were plotted in a multidimensional scaling (MDS) graph. The PCA graphs showed groups partially separated by cultivar and tissue evaluated both for chickpea (**Figure 1A**) and lentil (**Figure 1B**).

A total of 1,624 and 2,512 differentially expressed transcripts were identified after our cutoff between pairwise comparisons of chickpea (BX vs BA, FX vs FA, FX vs BX, and FA vs BA) and lentil (CmX vs CmA, CsX vs CsA, CsX vs CmX, and CsA vs CmA), respectively (**Supplementary Files S1**, **S2**). Among differentially expressed chickpea transcripts, a total of 94 (BX vs BA), 1,147 (FX vs FA), 974 (FX vs BX), and 282 (FA vs BA) were considered up- or down-regulated (**Figure 1C**). In contrast, in lentil, a total of 49 (CmX vs CmA), 829 (CsX vs CsA), 1,375 (CsX vs CmX), and 1,905 (CsA vs CmA) were considered up or down-regulated (**Figure 1D**). Taking together the eight pairwise comparisons in chickpea, the number of up-regulated transcripts ranged from 88 to 1,043 while the down-regulated transcripts ranged from 6 to 240 (**Figures 1C, E, F**). Meanwhile, in the pairwise comparisons of the eight lentil treatments, the number of up-regulated transcripts ranged from 13 to 1,226, while the down-regulated transcripts ranged from 36 to 681 (**Figures 1D, G, H**). Therefore, several transcripts were identified as differentially expressed in pairwise comparison between different tissues and contrasting cultivars.

### Differentially expressed transcript set enrichment analyses reveal the modulated biological processes

**Figure 2:**
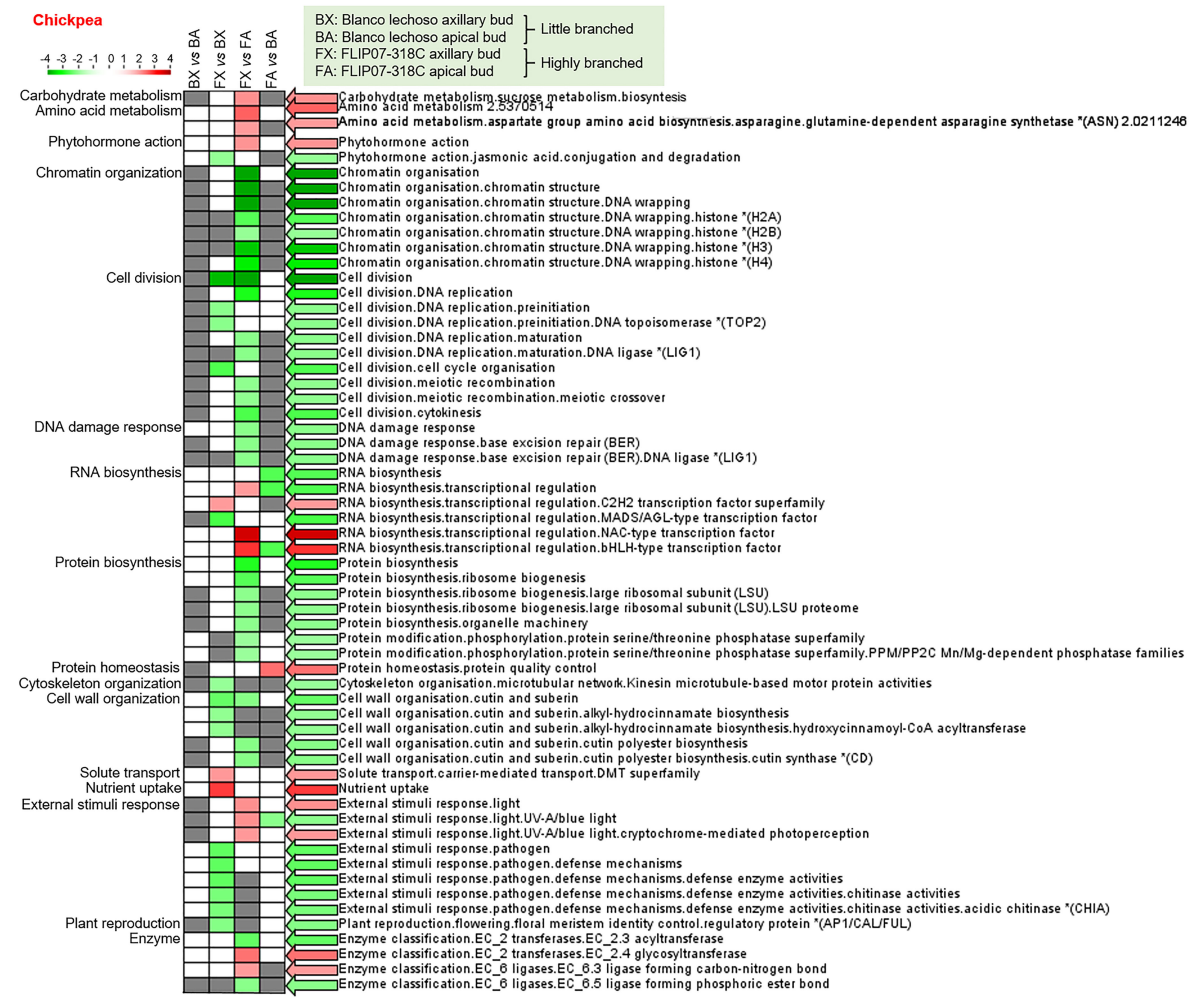
Transcript set enrichment categories for the two pairwise comparisons using the PageMan web tool. The green and red extremes represent the metabolic pathways differentially modulated between contrasting cultivars and tissues of chickpea. Only differentially expressed transcripts with FDR <0.05 and log(fold change) lower than -2 or greater than +2 were considered in the pathway analysis. The color intensity is correlated with the statistical significance based on the Wilcoxon test default implemented in the PageMan tool.

The enrichment analysis of differentially expressed transcript set from chickpea showed that jasmonic acid (JA) metabolism, cell division, DNA replication, cell cycle, RNA biosynthesis (MADS/AGL-type transcription factor), cell wall organization, and plant reproduction were significantly down-regulated, while RNA biosynthesis (C2H2 transcription factor), solute transport, and nutrient uptake were significantly up-regulated in axillary buds of cultivar Blanco lechoso (little branched) compared with the cultivar FLIP07–318C (highly branched) (**Figure 2**). In contrast, RNA biosynthesis and external stimuli response (UV-A/blue light) were significantly down-regulated, while protein homeostasis and protein quality control were significantly up-regulated in apical buds of cultivar Blanco lechoso (little branched) compared with the cultivar FLIP07–318C (highly branched) (**Figure 2**). Moreover, the enriched categories with differentially expressed transcripts between axillary and apical buds of the chickpea cultivar Blanco lechoso were not differentially modulated, while chromatin organization, cell division, DNA replication, cell division, cell cycle, DNA damage response, protein biosynthesis, protein phosphorylation, cell wall organization, acyltransferases (EC 2.3), and ligases (EC 6.5) were significantly down-regulated, while carbohydrate metabolism, amino acid metabolism, phytohormone action, RNA biosynthesis, external stimuli response (UV-A/blue light), and glycosyltransferases (EC 2.4), and ligases (EC 6.3) were up-regulated in apical buds of the chickpea cultivar FLIP07–318C compared with the axillary buds of the same cultivar (**Figure 2**).

**Figure 3:**
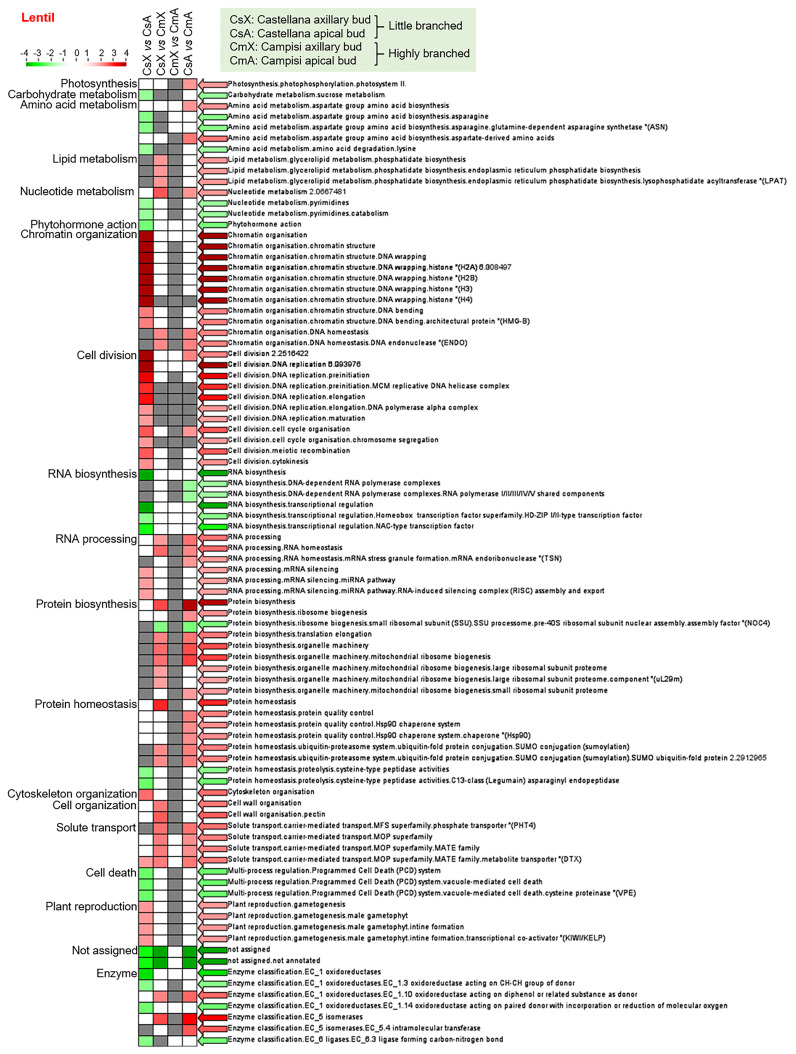
Transcript set enrichment categories for the two pairwise comparisons using the PageMan web tool. The green and red extremes represent the metabolic pathways differentially modulated between contrasting cultivars and tissues of lentil. Only differentially expressed transcripts with FDR <0.05 and log(fold change) lower than -2 or greater than +2 were considered in the pathway analysis. The color intensity is correlated with the statistical significance based on the Wilcoxon test default implemented in the PageMan tool.

Similarly, the enrichment analysis of differentially expressed transcript set from lentil showed that protein biosynthesis (pre-40S ribosomal subunit) was down-regulated, while lipid metabolism, nucleotide metabolism, chromatin organization, RNA processing, protein biosynthesis and homeostasis (quality control and ubiquitin-proteasome system), cell wall organization, solute transport, oxidoreductases (EC 1.10), and isomerases (EC 5) were up-regulated in axillary buds of cultivar Campisi (highly branched) compared with the cultivar Castellana (little branched) (**Figure 3**). In contrast, RNA biosynthesis and protein biosynthesis (pre-40S ribosomal subunit) were significantly down-regulated, while photosynthesis, amino acid metabolism, nucleotide metabolism, chromatin organization, cell division and cycle, RNA processing, protein biosynthesis, protein homeostasis, solute transport, oxidoreductases (EC 1.10), and isomerases (EC 5 and EC 5.4) were up-regulated in apical buds of cultivar Campisi (highly branched) compared with the cultivar Castellana (little branched) (**Figure 3**). In addition, the enriched categories with differentially expressed transcripts between axillary and apical buds of the lentil cultivar Castellana showed that carbohydrate metabolism, amino acid metabolism, nucleotide metabolism (pyrimidines), phytohormone action, RNA biosynthesis, protein homeostasis, proteolysis, programmed cell death, oxidoreductases (EC 1.3 and EC 1.14), and ligases (EC 6.3) were significantly down-regulated, while chromatin organization, cell division, DNA replication, cell division, cell cycle, RNA processing (silencing), cytoskeleton organization, solute transport (MATE family), and plant reproduction were up-regulated in apical buds of cultivar Castellana (little branched) compared with the axillary buds of the same cultivar (**Figure 3**). Meanwhile, the enriched categories with differentially expressed transcripts between axillary and apical buds of the lentil cultivar Campisi were not differentially modulated (**Figure 3**). Therefore, several biological processes were modulated in pairwise comparison between different tissues and contrasting cultivars of chickpea and lentil, highlighting hormonal pathways, cell cycle, RNA and protein synthesis, and plant development and reproduction.

### GO and KEGG pathway enrichment analyses reveal the functional profile of differentially expressed transcripts

The GO enrichment analyses were carried out with differentially expressed transcripts to evidence the biological mechanisms associated with little or highly branched. The GO enrichment analysis of differentially expressed transcript set from chickpea showed that several clusters were arranged to represent the categories associated with the photosystem, cytochrome P450, transmembrane, transport, stress protein, and secondary metabolism from up-regulated transcripts, while the categories associated with the cell division, cell cycle, cell organization, transferases, secondary metabolism, oxidoreductases, and DNA transcription were represented from down-regulated transcripts in axillary buds of cultivar Blanco lechoso (little branched) compared with the cultivar FLIP07–318C (highly branched) (**Supplementary File S3**). In contrast, the categories associated with the response to heat stress, chaperone, DnaJ transcription factors, and cell wall were up-regulated, while the categories associated with sugar metabolism, DNA transcription, oxidoreductase, metal binding, and RNA binding were down-regulated in apical buds of cultivar Blanco lechoso compared with the cultivar FLIP07–318C (**Supplementary File S3**). In addition, the categories associated with DNA transcription, oxidoreductase, dioxygenase, and peptidase were up-regulated, while no category was down-regulated in apical buds of cultivar Blanco lechoso compared with axillary buds of the same cultivar (**Supplementary File S3**). Meanwhile, the categories associated with transmembrane, oxidoreductase, dioxygenase, metal binding, cytochrome P450, gibberellin biosynthesis, amino acid transport, nitrate assimilation, sugar metabolism, DNA binding, kinases, and secondary metabolism were up-regulated, while the categories associated with genome integrity, histone, lipid metabolism, DNA methylation, DNA binding, and metal binding were down-regulated in apical buds of cultivar FLIP07–318C compared with axillary buds of the same cultivar (**Supplementary File S3**).

Similarly, the GO enrichment analysis of differentially expressed transcript set from lentil showed that several categories associated with the transmembrane transport, lipid metabolism, cytoskeleton organization, metal binding, cytochrome P450, sugar metabolism, response to endoplasmic reticulum stress, and proteolysis were up-regulated, while the categories associated with ATP-binding, kinases, signaling, transport, DNA binding, and ubiquitin were down-regulated in axillary buds of cultivar Campsi (highly branched) compared with the cultivar Castellana (little branched) (**Supplementary File S3**). In addition, the categories associated with lipid metabolism, transmembrane transport, peptidase, metal binding, cell cycle, and cytoskeleton organization were up-regulated, while the categories associated with ATP-binding, sugar metabolism, lipid metabolism, glucosyltransferase, kinase, chaperone, metal binding, DNA binding, signaling, and chloroplast stroma were down-regulated in apical buds of cultivar Campsi compared with the cultivar Castellana (**Supplementary File S3**). Meanwhile, the categories associated with the DNA-binding, cell cycle, genome integrity, ATP-binding, and zinc finger were up-regulated transcripts, while the categories associated with sugar metabolism, oxidoreductase, cytochrome P450, metal binding, DNA-binding, signaling, kinases, hormone biosynthesis, secondary metabolism, and transmembrane were down-regulated in apical buds of cultivar Castellana compared with axillary buds of the same cultivar (**Supplementary File S3**). In the same sense, no GO category was up-regulated, while the categories associated with metabolic pathways and kinase activity were down-regulated in apical buds of cultivar Campisi compared with axillary buds of the same cultivar (**Supplementary File S3**). Therefore, the differentially expressed transcripts in chickpea modulated for greater energy production and lower cell cycle in axillary buds while greater metabolism and lower development in apical buds of the highly branched cultivar. Meanwhile, in lentil these transcripts modulated for lower metabolism and proteolysis and greater signaling in axillary buds while lower cell cycle and higher metabolism in apical buds of the highly branched cultivar. Similarly, KEGG pathway enrichment analyses on differentially expressed transcripts among pairwise comparisons of genotypes and tissues showed significant enrichments for pathways as metabolic processes, biosynthesis of secondary metabolites, and signal transduction for both chickpea (**Supplementary Figures S2A-D**) and lentil (**Supplementary Figures S3A-D**).

### Sucrose-triggered signaling pathway

Representative sets of differentially expressed transcripts were identified as interconnected in the sucrose-triggered signaling pathway in the comparison between apical and axillary buds and contrasting cultivars of chickpea and lentil (**Table 1**). From the chickpea datasets, 19 main transcripts involved in carbohydrate transport, inositol transport, raffinose biosynthesis, sucrose biosynthesis, dihydroxyacetone phosphate biosynthesis, carbohydrate efflux, sugar sensing, sucrose transport, and sugar signaling were identified as differentially expressed (**Table 1**). The main changes observed in chickpea were the up-regulation of all these genes, except for the down-regulation of *CaGPT2* and *CaSWEET3* genes, in axillary buds compared to apical buds of cultivar FLIP07–318C (highly branched). Meanwhile, the *CaSUS3* and *CaSnRK1/KING1* genes were up-regulated in the axillary buds compared with apical buds of cultivar Blanco lechoso (little branched). Similarly, all 19 genes were also considered up-regulated in the axillary buds of cultivar FLIP07–318C (highly branched) compared to the axillary buds of cultivar Blanco lechoso (little branched), except for the down-regulation of the *CaSWEET3* gene. Also, the *CaGPT2*, *CaSWEET4*, and *CaSIP2* genes were considered up-regulated while *CaSIP1* and *CaFBP1* were down-regulated in the apical buds of cultivar FLIP07–318C compared to cultivar Blanco lechoso (little branched). Therefore, these chickpea data suggest that metabolism and sucrose-mediated signaling are more active in axillary buds of the highly branched cultivar compared with apical buds of the same cultivar and axillary buds of the little branched cultivar.

Table 1Expression profile of major genes involved in the sucrose-triggered signaling pathway in each pairwise comparison for both chickpea and lentil genotypes and tissues.

**Table 1:** 

Chickpea								
Gene name	Function	Gene ID		Transcript ID	BX_vs BA	FX_vs FA	FX_vs BX	FA_vs BA
*CaGPT2*	carbohydrate transport	Ca_03358		XM_004486075	0	-1.91	0	2.55
*CaINT1*	inositol transport	Ca_18506		XM_004488224	0	2.76	1.31	0
*CaINT2*	inositol transport	Ca_18504		XM_004488226	0	3.31	2.37	0
*CaSIP1*	raffinose biosynthesis	Ca_12601		XM_004489170	0	2.62	0	-1.83
*CaSPS3F*	sucrose biosynthesis	Ca_15248		XM_004491268	0	5.56	6.33	0
*CaFBA*	dihydroxyacetone phosphate	Ca_09753		XM_004491482	0	4.15	3.78	0
*CaZIP2*	carbohydrate efflux	Ca_07345		XM_004493575	0	1.85	2.22	0
*CaSUS3*	sucrose biosynthesis	Ca_00979		XM_004494334	1.18	2.81	1.81	0
*CaSWEET1*	carbohydrate transport	Ca_03475		XM_004498321	0	2.36	2.13	0
*CaSWEET3*	carbohydrate transport	Ca_13079		XM_004498340	0	-2.92	-3.06	0
*CaSWEET4*	carbohydrate transport	Ca_03924		XM_004502557	0	0	2.04	3.19
*CaSWEET14*	carbohydrate transport	Ca_05699		XM_004503721	0	2.65	2.52	0
*CaSWEET12*	carbohydrate transport	Ca_01418		XM_004503722	0	4.58	5.31	0
*CaEXL2.1*	sugar sensing	Ca_05262		XM_004504754	0	3.57	1.59	0
*CaEXL2.2*	sugar sensing	Ca_22023		XM_004504903	0	2.37	1.78	0
*CaSUC2*	sucrose transport	Ca_27098		XM_004515533	0	2.88	1.78	0
*CaSnRK1/KING1*	sugar signaling	Ca_08758		XM_004515759	1.43	3.25	1.36	0
*CaFBP1*	sucrose biosynthesis	Ca_26449		XM_004516586	0	4.07	2.67	-1.72
*CaSIP2*	sucrose biosynthesis	Ca_07255		XM_012713836	0	2.78	3.08	0.81

**Table:** 

Lentil								
Gene name	Function	Gene ID			CmX_vs CmA	CsX_vs CsA	CsX_vs CmX	CsA_vs CmA
*LcFBA*	dihydroxyacetone phosphate	Lcu.2RBY.2g001250			0	-2.14	0	1.32
*LcINT2*	inositol transport	Lcu.2RBY.2g011220			0	0	0	4.07
*LcINT1*	inositol transport	Lcu.2RBY.3g002380			0	-2.75	0	2.82
*LcSWEET10*	carbohydrate transport	Lcu.2RBY.3g059330			0	-2.86	0	2.21
*LcINT3*	inositol transport	Lcu.2RBY.4g010830			0	0	0	4
*LcEXL2.1*	sugar sensing	Lcu.2RBY.4g060530			0	-3.3	0	2.79
*LcEXL2.2*	sugar sensing	Lcu.2RBY.4g060540			0	-3.71	0	2.52
*LcSWEET11*	carbohydrate transport	Lcu.2RBY.4g074460			0	-2.89	0	3.27
*LcSuSy1*	sucrose to fructose and glucose	Lcu.2RBY.5g020600			0	0	-7.37	-6.92
*LcSPS*	sucrose biosynthesis	Lcu.2RBY.5g063440			0	-2.81	0	3.28
*LcSuSy2*	sucrose to fructose and glucose	Lcu.2RBY.6g058720			0	0	-6.89	-3.83
*LcSuSy3*	sucrose to fructose and glucose	Lcu.2RBY.6g064170			0	-5.96	0	2.78
*LcUST1*	UDP-sugar transport	Lcu.2RBY.7g027450			0	0	-3.83	-7.04
*LcSnRK1/KING1*	sugar signaling	Lcu.2RBY.7g057850			0	-3.88	0	2.26
*LcSUC1*	sucrose transport	Lcu.2RBY.7g077150			0	0	0	-2.55

0: statistically non-significant, *p*-value <0.05 and FDR <0.05. BX: Blanco lechoso axillary bud, BA: Blanco lechoso apical bud, FX: FLIP07–318C axillary bud, FA: FLIP07–318C apical bud, CsX, Castellana axillary bud; CsA, Castellana apical bud; CmX, Campisi axillary bud; and CmA, Campisi apical bud. Blanco lechoso: little branched; FLIP07–318C: highly branched; Castellana: little branched; and Campisi: highly branched.

Likewise, from the lentil datasets, 15 main transcripts involved in dihydroxyacetone phosphate biosynthesis, inositol transport, carbohydrate transport, sugar sensing, sucrose degradation, UDP-sugar transport, sugar signaling, and sucrose transport were identified as differentially expressed (**Table 1**). The main changes observed in lentil were the down-regulation of almost all these genes in axillary buds compared to apical buds of cultivar Castellana (little branched). Meanwhile, the *LcSuSy1*, *LcSuSy2*, and *LcUST1* genes were down-regulated in the axillary buds of cultivar Castellana (little branched) compared with Campisi (highly branched) (**Table 1**). Similarly, the up-regulation of almost all these genes in apical buds of cultivar Castellana compared to cultivar Campisi was observed, except for the down-regulation of *LcSuSy1*, *LcSuSy2*, *LcUST1*, and *LcSUC1* genes. In particular, the expression of this gene set showed no changes in the expression profile between axillary and apical buds of highly branched cultivar. These lentil data suggest that metabolism and sucrose-mediated signaling are more active in the apical buds of little branched cultivar compared to highly branched cultivar, while this process is balanced between apical and axillary buds of highly branched cultivar. Therefore, the metabolism and sucrose-mediated signaling pathway have a strong positive correlation in the increased branching or apical dominance in both chickpea and lentil.

### Trehalose-6-phosphate-triggered signaling pathway

Several *trehalose-6-phosphate synthase* (TPS) transcripts were differentially expressed in the comparison between apical and axillary buds and contrasting cultivars of chickpea and lentil (**Table 2**). In particular, four *CaTPS* genes were up-regulated in apical buds of cultivar FLIP07–318C (highly branched) compared with axillary buds of the same cultivar, while three of these genes were down-regulated in apical buds of cultivar Blanco lechoso (little branched) compared with apical buds of cultivar FLIP07–318C. Likewise, four *LcTPS* genes were down-regulated in apical buds of cultivar Castellana (little branched) compared with axillary buds of the same cultivar, while these same genes were up-regulated in apical buds of cultivar Campisi (highly branched) compared with the cultivar Castellana. Meanwhile, two *trehalose-6-phosphate phosphatase* (TPP) genes were also up- and down-regulated when compared axillary and apical buds of cultivar FLIP07–318C, and up-regulated in these tissues when compared both cultivars (**Table 2**). Likewise, three *LcTPP* genes were down-regulated, mainly in apical and axillary buds of cultivar Campisi compared with the cultivar Castellana. For instance, both *hexokinase-1* (HXK1) genes were down-regulated in apical buds of cultivar FLIP07–318C compared with axillary buds of the same cultivar, and in axillary buds of cultivar Blanco lechoso compared with the cultivar FLIP07–318C (**Table 2**). Meanwhile, only the *LcHXK1.2* gene was up-regulated in apical buds of cultivar Castellana compared with axillary buds of the same cultivar. Likewise, the *CaSnRK1/KIN10* and *CaSnRK1/KIN11* genes were respectively down-regulated in axillary buds of cultivar Blanco lechoso compared with the cultivar FLIP07–318C, and up-regulated in apical buds of cultivar FLIP07–318C compared with axillary buds of the same cultivar (**Table 2**). Likewise, *LcSnRK1/KIN11* gene was down-regulated in apical buds of cultivar Castellana compared with the axillary buds of the same cultivar, while was up-regulated in apical buds of cultivar Campisi compared with the cultivar Castellana. Lastly, the *sugar transporter protein 1* (STP1) gene, which is not directly related to the trehalose-6-phosphate pathway, but contributes to the regulation of genes involved in shoot branching through carbon partitioning, was up-regulated in apical buds of cultivar FLIP07–318C compared with axillary buds of the same cultivar, and also up-regulated in axillary buds of cultivar Blanco lechoso compared with the cultivar FLIP07–318C (**Table 2**). Meanwhile, the *LcSTP1* gene was down-regulated in apical buds of cultivar Castellana compared with axillary buds of the same cultivar, and up-regulated in apical buds of cultivar Campisi compared with the cultivar Castellana. Therefore, these collective data showed that the trehalose-6-phosphate biosynthesis and signaling pathway and TPS1-mediated signaling were differentially modulated between different tissues and contrasting cultivars of both chickpea and lentil.

Table 2Expression profile of major genes involved in the trehalose-6-phosphate-triggered signaling pathway in each pairwise comparison for both chickpea and lentil genotypes and tissues.

**Table 2:** 

Chickpea							
Gene name		Gene ID	Transcript ID	BX_vs BA	FX_vs FA	FX_vs BX	FA_vs BA
*CaTPS1*		Ca_12942	XM_012712537	0	0	0	0
			XM_004488930				
			XM_004488929				
*CaTPS2*		Ca_08642	XM_004504195	0	0	0	0
*CaTPS3*		Ca_26853	XM_027330909	0	0	0	0
*CaTPS4*		Ca_10407	XM_004503283	0	1.95	0	-1.57
*CaTPS5*		Ca_05529	XM_004496995	0	0	0	0
*CaTPS6*		Ca_15155	XM_004498177	1.13	2.8	0	-1.13
*CaTPS7*		Ca_16322	XM_004505410	0	0	0	0
*CaTPS8*		Ca_07508	XM_027335107	0	0	0	0
			XM_004501888				
			XM_004501889				
			XM_004501890				
*CaTPS9*		Ca_14509	XM_004509783	0	1.17	0	0
*CaTPS10*		Ca_03283	XM_004507745	0	2.88	0	-2.3
*CaTPS11*		Ca_03956	XM_004502560	0	0	0	0
			XM_027334815				
			XM_027334814				
*CaTPS12*		Ca_21271	XM_027331164	0	0	0	0
*CaTPP1*		Ca_26079	XM_004513697	0	0	0	0
*CaTPP2*		Ca_12686	XM_004500559	0	-1.67	0	2.01
		Ca_16633					
*CaTPP3*		Ca_09577	XM_004504003	0	1.92	1.26	0
*CaTPP4*		Ca_19952	XM_004514466	0	0	0	0
			XM_027330512				
*CaTPP5*		Ca_24715	XM_004516296	0	0	0	0
		Ca_24716	XM_027331128				
*CaTPP6*		Ca_16320	XM_027331355	0	0	0	0
*CaTRE1*		Ca_05859	XM_027336125	0	0	0	0
			XM_004503518				
			XM_004503519				
*CaHXK1.1*		Ca_05924	XM_004503434	0	-0.44	0	0
*CaHXK1.2*		Ca_10135	XM_004510258	0	-1.59	-0.96	0
*CaHXK1.3*		Ca_17861	XM_004512996	0	-0.62	-0.71	0
*CaSnRK1/KIN10*		Ca_10492	XM_004489368	0	0	-0.36	0
*CaSnRK1/KIN11*		Ca_22087	XM_004491795	0	0.63	0	0
*CaSTP1*		Ca_22023	XM_004504903	0	2.36	1.77	0

**Table:** 

Lentil							
Gene name		Gene ID		CmX_vs CmA	CsX_vs CsA	CsX_vs CmX	CsA_vs CmA
*LcTPS1*		Lcu.2RBY.L003530		0	0	0	1.24
*LcTPS2*		Lcu.2RBY.L020570		0	0	0	0
*LcTPS3*		Lcu.2RBY.2g076850		0	0	0	0
*LcTPS4*		Lcu.2RBY.6g000780		0	0	0	0
*LcTPS5*		Lcu.2RBY.1g073150		0	0	0	0
*LcTPS6*		Lcu.2RBY.1g007850		0	-1.48	0	1.78
*LcTPS7*		Lcu.2RBY.4g047840		0	0	0	0
*LcTPS8*		Lcu.2RBY.4g081030		0	-1.05	0	1.28
*LcTPS9*		Lcu.2RBY.L000760		0	-1.5	0	1.96
*LcTPS10*		Lcu.2RBY.7g075560		0	-2.73	0	3.16
*LcTPS11*		Lcu.2RBY.3g052770		0	0	0	0
*LcTPP1*		Lcu.2RBY.5g040340		0	0	0	-2.71
*LcTPP2*		Lcu.2RBY.4g067950		0	0	0	0
*LcTPP3*		Lcu.2RBY.3g033740		0	0	-1.69	0
*LcTPP4*		Lcu.2RBY.4g016870		0	-1.28	-1.2	0
*LcTPP5*		Lcu.2RBY.7g056410		0	0	0	0
*LcTPP6*		Lcu.2RBY.3g001930		0	0	0	0
*LcTPP7*		Lcu.2RBY.L018740		0	0	0	0
*LcTRE1*		Lcu.2RBY.4g077780		0	0	0	0
*LcHXK1.1*		Lcu.2RBY.4g078960		0	0	0	0
*LcHXK1.2*		Lcu.2RBY.7g001330		0	0.81	0	0
*LcHXK1.3*		Lcu.2RBY.2g069450		0	0	0	0
*LcSnRK1/KIN10*		Lcu.2RBY.2g054810		0	0	0	0
*LcSnRK1/KIN11*		Lcu.2RBY.5g061730		0	-0.73	0	0.56
*LcSTP1*		Lcu.2RBY.4g057720		0	-1.39	0	0.87

0: statistically non-significant, *p*-value <0.05 and FDR <0.05. BX: Blanco lechoso axillary bud, BA: Blanco lechoso apical bud, FX: FLIP07–318C axillary bud, FA: FLIP07–318C apical bud, CsX, Castellana axillary bud; CsA, Castellana apical bud; CmX, Campisi axillary bud; and CmA, Campisi apical bud. Blanco lechoso: little branched; FLIP07–318C: highly branched; Castellana: little branched; and Campisi: highly branched.

### Different hormonal signaling pathways

Additional enrichment analyses were focused on hormonal pathways modulated in the comparison of the same tissue between little and highly branched genotypes. The comparison of axillary buds of cultivar Blanco lechoso (little branched) with the cultivar FLIP07–318C (highly branched) showed that several up-regulated transcripts were involved in abscisic acid (ABA) biosynthesis and auxin, ethylene, cytokinin and brassinosteroid, strigolactone biosynthesis and signal transduction, while down-regulated categories were involved in auxin conjugation and degradation, ethylene biosynthesis, gibberellin biosynthesis, and JA biosynthesis, conjugation and degradation (**Supplementary File S4**). In addition, the up-regulated transcripts involved in auxin biosynthesis, JA biosynthesis, and strigolactones biosynthesis, while the down-regulated transcripts involved in auxin conjugation and degradation, cytokinin biosynthesis and signal transduction, gibberellin biosynthesis, and JA biosynthesis were represented in apical buds of cultivar Blanco lechoso contrasted with the cultivar FLIP07–318C (**Supplementary File S4**). Meanwhile, up-regulated transcripts involved in ABA biosynthesis, auxin conjugation and degradation, ethylene biosynthesis, JA biosynthesis, conjugation and degradation were represented in apical buds of cultivar Blanco lechoso contrasted with axillary buds of the same cultivar, while the down-regulated transcripts did not impact hormonal pathways (**Supplementary File S4**). In the same sense, up-regulated transcripts involved in ABA biosynthesis, signaling and degradation, auxin signaling and degradation, cytokinin biosynthesis and signaling, ethylene biosynthesis, gibberellin biosynthesis, signal transduction and degradation, and JA biosynthesis and degradation, while down-regulated transcripts involved in cytokinin signaling were represented in the apical buds of cultivar FLIP07–318C contrasted with axillary buds of the same cultivar (**Supplementary File S4**).

Similarly, the differentially expressed transcript set from lentil showed that several up-regulated transcripts involved in auxin signaling, cytokinin degradation, and JA biosynthesis, while the down-regulated transcripts involved in ABA signaling and degradation, and auxin degradation were represented in the axillary buds of cultivar Campisi (highly branched) contrasted with the cultivar Castellana (little branched) (**Supplementary File S4**). In addition, up-regulated transcripts involved in ABA biosynthesis and transport, auxin signaling, auxin signaling, brassinosteroid biosynthesis and signaling, cytokinin biosynthesis and degradation, ethylene biosynthesis and signaling, gibberellin biosynthesis, signaling and degradation, and JA biosynthesis, signaling and degradation were represented in the apical buds of cultivar Campisi contrasted with the cultivar Castellana (**Supplementary File S4**). Meanwhile, the up-regulated transcripts involved in JA degradation, and down-regulated transcripts involved in ABA biosynthesis, signaling and degradation, auxin transport, brassinosteroid signaling, cytokinin biosynthesis, ethylene biosynthesis and signaling, gibberellin biosynthesis, signaling and degradation, JA biosynthesis, and strigolactones signaling were represented in the apical buds of cultivar Castellana contrasted with axillary buds of the same cultivar (**Supplementary File S4**). In the same sense, the down-regulated transcripts involved in JA biosynthesis were represented in the apical buds of cultivar Campisi contrasted with axillary buds of the same cultivar, while the up-regulated transcripts did not impact hormonal pathways (**Supplementary File S4**). Therefore, the differentially expressed transcripts in chickpea modulated the ABA, auxin, brassinosteroid, cytokinin, ethylene, gibberellin, JA, and strigolactones in axillary buds while auxin, JA, and strigolactones in apical buds of the highly branched cultivar compared with little branched cultivar. Meanwhile, in lentil these transcripts modulated auxin, cytokinin, JA, and ABA in axillary buds while ABA, auxin, brassinosteroid, cytokinin, ethylene, gibberellin, and JA in apical buds of the highly branched cultivar compared with little branched cultivar.

### Cytokinin and auxin signaling pathways

Several transcripts enriched for cytokinin and auxin pathways were found to be differentially expressed in contrasting chickpea and lentil cultivars (**Supplementary File S4**; **Table 3**). In the chickpea dataset, the 10 main genes involved in the cytokinin pathway are annotated as involved in cytokinin degradation, transmembrane receptor, biosynthesis, signaling, and transport, while the five main genes associated with the auxin pathway are annotated as involved in auxin signaling, biosynthesis, transport, and degradation (**Table 3**). All of these genes were up-regulated in axillary buds compared to apical buds of the FLIP07–318C cultivar (highly branched), except for the down-regulation of *CaAHP6* gene. In contrast, only *CaILR1* gene was up-regulated in axillary buds compared to apical buds of the Blanco lechoso cultivar (little branched). Meanwhile, nine genes were up-regulated and two genes (*CaAHP6* and *CaILR1*) were down-regulated in axillary buds of the highly branched cultivar compared with the little branched cultivar. Likewise, six genes were down-regulated and two genes (*CaAux/IAA14* and *CaYUC10*) were up-regulated in apical buds of the highly branched cultivar compared with the little branched cultivar.

Table 3Expression profile of major genes involved in the cytokinin and auxin signaling pathways in each pairwise comparison for both chickpea and lentil genotypes and tissues.

**Table 3:** 

Chickpea							
Gene name	Function	Gene ID	Transcript ID	BX_vs BA	FX_vs FA	FX_vs BX	FA_vs BA
Cytokinin							
*CaCKX3*	degradation	Ca_20618	XM_004488000	0	1.86	2.56	0
*CaAHK1*	receptor	Ca_09957	XM_004509318	0	2.29	1.86	0
*CaLOG3*	biosynthesis	Ca_17140	XM_004500892	0	1.81	0	-2.01
*CaAHP1*	signaling	Ca_00554	XM_004486025	0	2.25	0	-1.9
*CaAHP2*	signaling	Ca_01193	XM_004494591	0	3.66	2.18	0
*CaAHP4*	signaling	Ca_10140	XM_004510253	0	3.01	0	-2.76
*CaAHP6*	signaling	Ca_02886	XM_004486606	0	-2.21	-1.7	0
*CaARR1*	signaling	Ca_15151	XM_004498184	0	2.03	0	0
*CaABCG21*	transport	Ca_08447	XM_004496158	0	1.79	2.19	0
*CaCYP735A1*	biosynthesis	Ca_03562	XM_004495795	0	5.5	5.22	0
Auxin							
*CaAux/IAA14*	signaling	Ca_12139	XM_004495335	0	2.32	3.67	0.98
*CaYUC10*	biosynthesis	Ca_00921	XM_004494264	0	1.55	3.72	2.09
*CaPIN2*	transport	Ca_15089	XM_004498250	0	2.07	1.11	-1.22
*CaILR1*	degradation	Ca_14555	XM_004509830	3.72	5.23	-3.5	-5.01
*CaAux/IAA2*	signaling	Ca_06692	XM_012718021	0	5.74	5.34	-1.92

**Table:** 

Lentil							
Gene name	Function	Gene ID		CmX_vs CmA	CsX_vs CsA	CsX_vs CmX	CsA_vs CmA
Cytokinin							
*LcAHK1*	receptor	Lcu.2RBY.5g018950		0	0	-9.04	-10.93
*LcARR12*	signaling	Lcu.2RBY.3g049570		0	0	-7.2	-8.01
*LcCYP735A1*	biosynthesis	Lcu.2RBY.1g054400		0	-5.61	0	2.93
*LcZOG1*	degradation	Lcu.2RBY.1g022770		0	-0.83	10.11	10.94
Auxin							
*LcPILS1*	transport	Lcu.2RBY.5g012110		0	-2.17	0	3.36
*LcAux/LAX1*	transport	Lcu.2RBY.5g024480		0	0	8.52	5.72
*LcAux/LAX2*	transport	Lcu.2RBY.6g061550		0	0	6.84	5.3
*LcTMK1*	receptor	Lcu.2RBY.4g081270		0	0	-4.28	-3.85
*LcTMK3*	receptor	Lcu.2RBY.3g010920		0	0	-7.73	-10.21
*LcTMK2*	receptor	Lcu.2RBY.4g015900		0	0	3.46	2.08
*LcAux/IAA14*	signaling	Lcu.2RBY.7g001340		0	-1.85	0	2.21

0: statistically non-significant, *p*-value <0.05 and FDR <0.05. BX: Blanco lechoso axillary bud, BA: Blanco lechoso apical bud, FX: FLIP07–318C axillary bud, FA: FLIP07–318C apical bud, CsX, Castellana axillary bud; CsA, Castellana apical bud; CmX, Campisi axillary bud; CmA, Campisi apical bud. Blanco lechoso: little branched; FLIP07–318C: highly branched; Castellana: little branched; and Campisi: highly branched.

In the lentil dataset, the four main genes involved in the cytokinin pathway are annotated as involved in cytokinin transmembrane receptor, signaling, biosynthesis, and degradation, while the seven main genes associated with the auxin pathway are annotated as involved in auxin transport, transmembrane receptor, and signaling (**Table 3**). Four of these genes (*LcCYP35A1*, *LcZOG1*, *LcPILS1*, and *LcAux/IAA14*) were down-regulated in axillary buds compared to apical buds of the Castellana cultivar (little branched). In contrast, none of these genes were differentially expressed in axillary buds compared to apical buds of the Campisi cultivar (highly branched). Meanwhile, four genes were up-regulated and another four genes were down-regulated in the axillary buds of the little branched cultivar compared with the highly branched cultivar. Likewise, four genes were down-regulated and another seven genes were up-regulated in apical buds of the little branched cultivar compared with the highly branched cultivar. Therefore, since it is well known that the cytokinin and auxin pathways act on each other, providing regulatory feedback to control apical dominance and plant branching, the differential expression profile of several genes involved in different functions suggests that these two hormonal pathways play a remarkable role in modulating the branching of contrasting chickpea and lentil cultivars.

### Strigolactones signaling pathway

The *CCD* subfamily genes (Basso et al., 2023) and *SMAX/SMXL* family genes (Basso et al., 2024a) of chickpea and lentil, both involved in carotenoids and dependent and independent strigolactones and karrikins pathways, were also exploited to evidence the strigolactones signaling modulation and eventual association with the branching phenotype. In particular, the CCD subfamily contains genes involved in the degradation of carotenoids for the production of strigolactones and other volatile and non-volatile compounds, while chickpea and lentil *SMAX1/SMXL1* genes are involved in the strigolactones and karrikins-dependent signaling pathway for regulation of shoot branching and hairy root elongation (Basso et al., 2023; Basso et al., 2024a). Meanwhile, the chickpea and lentil *SMXL6* to *SMXL8* genes are involved in the strigolactones-dependent signaling pathway for the regulation of shoot branching and elongation, and the chickpea and lentil *SMXL2* and *SMXL3* genes are involved in the strigolactones- and karrikins-independent signaling pathway for the regulation of phloem formation (Basso et al., 2024a). In this study, the *CaCCD2*, *CaSMAX1/SMXL1*, *CaSMXL2*, and *CaSMXL7* genes were up-regulated while the *CaSMXL5* gene was down-regulated in the apical buds of cultivar FLIP07–318C (highly branched) and axillary buds of cultivar Blanco lechoso (little branched) contrasted with axillary buds of the cultivar FLIP07–318C (**Table 4**). Meanwhile, the *LcCCD1*, *LcCCD5*, *LcSMAX1/SMXL1*, *LcSMXL6*, *LcSMXL7*, and *LcBRC1* genes were down-regulated in the apical buds of cultivar Castellana (little branched) compared with axillary buds of the same cultivar (**Table 4**). In addition, the *LcCCD1*, *LcCCD5*, *LcSMXL3*, and *LcSMXL7* genes were up-regulated in the apical buds of cultivar Campisi (highly branched) contrasted with the cultivar Castellana (**Table 4**). Therefore, the strigolactones biosynthesis and signaling pathway is differentially modulated between different tissues and contrasting cultivars of both chickpea and lentil and this differential modulation is marginally associated with the different branching profiles of the plants.

Table 4Expression profile of major genes of the carotenoids and strigolactones pathway involved in the plant branching in each pairwise comparison for both chickpea and lentil genotypes and tissues.

**Table 4:** 

Chickpea						
Gene name	Gene ID	Transcript ID	BX_vs BA	FX_vs FA	FX_vs BX	FA_vs BA
*CaCCD1*	Ca_10684	XM_004512251	0	0	0	0
*CaCCD2*	Ca_10683	XM_004512251	0	4.45	4.62	0
*CaCCD3*	Ca_01903	XM_004501106	0	0	0	0
*CaCCD4*	Ca_10867	XM_004513878	0	0	0	0
*CaCCD5*	Ca_01909	XM_027334990	0	0	0	0
*CaSMAX1/SMXL1*	Ca_03282	XM_004507746	0	1.14	0.58	0
*CaSMXL2*	Ca_14415	XM_004497611	0	0.80	0	0
*CaSMXL3*	Ca_08355	XM_004496060	0	0	0	0
*CaSMXL4*	Ca_22117	XM_004487952	0	0	0	0
*CaSMXL5*	Ca_03214	XM_004507845	0	-0.81	-0.55	0
*CaSMXL6*	Ca_09043	XM_004500211	0	0	0	0
*CaSMXL7*	Ca_14279	XM_004490545	0	1.25	0.85	0
*CaSMXL8*	Ca_13409	XM_004501105	0	0	0	0
*CaSMXL9*	Ca_20371	XM_012715065	0	0	0	0
*CaBRC1*	Ca_06609	XM_004508517	0	0	0	0
*CaTiE1*	Ca_17893	XM_004512959	0	0	0	0
*CaLAP1*	Ca_12381	XM_004509697	0	0	0	0
*CaBES1*	Ca_04963	XM_004500981	0	0	0	0
*CaCXE15*	Ca_15216	XM_004506191	0	0	0	0

**Table:** 

Lentil						
Gene name	Gene ID		CmX_vs CmA	CsX_vs CsA	CsX_vs CmX	CsA_vs CmA
*LcCCD1*	Lcu.2RBY.7g016190		0	-0.99	0	0.87
*LcCCD2*	Lcu.2RBY.5g012290		0	0	0	0
*LcCCD3*	Lcu.2RBY.6g017700		0	0	0	0
*LcCCD4*	Lcu.2RBY.3g069140		0	0	0	0
*LcCCD5*	Lcu.2RBY.7g016210		0	-0.77	0	0.77
*LcCCD6*	Lcu.2RBY.3g069000		0	0	0	0
*LcSMAX1/SMXL1*	Lcu.2RBY.7g075550		0	-1.08	0	0
*LcSMXL2*	Lcu.2RBY.1g030760		0	0	0	0
*LcSMXL3*	Lcu.2RBY.1g050370		0	0	0	0.97
*LcSMXL4*	Lcu.2RBY.2g022070		0	0	0	0
*LcSMXL5*	Lcu.2RBY.7g074400		0	0	0	0
*LcSMXL6*	Lcu.2RBY.3g027500		0	-0.58	0	0
*LcSMXL7*	Lcu.2RBY.5g047590		0	-1.33	0	1.20
*LcSMXL8*	Lcu.2RBY.3g037360		0	0	0	0
*LcSMXL9*	Lcu.2RBY.1g009790		0	0	0	0
*LcBRC1*	Lcu.2RBY.7g064070		0	-5.95	0	0
*LcTiE1*	Lcu.2RBY.2g068860		0	0	0	0
*LcLAP1*	Lcu.2RBY.7g030270		0	0	0	0
*LcBES1*	Lcu.2RBY.3g070670		0	0.53	0	0
*LcCXE15*	Lcu.2RBY.6g062590		0	0	0	0

0: statistically non-significant, *p*-value <0.05 and FDR <0.05. BX: Blanco lechoso axillary bud, BA: Blanco lechoso apical bud, FX: FLIP07–318C axillary bud, FA: FLIP07–318C apical bud, CsX, Castellana axillary bud; CsA, Castellana apical bud; CmX, Campisi axillary bud; CmA, Campisi apical bud. Blanco lechoso: little branched; FLIP07–318C: highly branched; Castellana: little branched; and Campisi: highly branched.

### Branching-related transcription factors

Several transcription factors with notable involvement in the regulation of plant branching were particularly monitored in the RNA-seq data of chickpea and lentil (**Table 5**). The first gene set corresponds to the transcription factor known as involved in the regulation of axillary branching (**Supplementary Figure S1**) as well as other transcription factors with similar functions (Zhang et al., 2022). Among them, the *CaEXB1*, *CaAGL8*, and *CaWOX4* genes which are considered positive regulators of plant branching were found as down-regulated in axillary buds of chickpea cultivar Blanco lechoso (little branched) compared with FLIP07–318C (highly branched). In addition, the *CaHB21*, *CaHB40*, and *CaHB53* genes which are considered negative regulators of the plant branching were more up-regulated in the apical buds of cultivar FLIP07–318C compared with axillary buds of the same cultivar, suggesting a potential inhibition of apical branches and increased axillary activity (**Table 5**). In addition, the *CaBAS1* gene, which is positively regulated by the *CaLOB1* gene and considered a negative regulator of plant branching by negatively regulating brassinosteroids, was up-regulated in axillary buds of cultivar Blanco lechoso compared with FLIP07–318C. Meanwhile, the *LcLOF2* gene which is considered a positive regulator of plant branching was found up-regulated in axillary buds of lentil cultivar Campisi (highly branched) compared with Castellana (little branched). In addition, the *LcAS1*, *LcHB21*, *LcHB53*, and *LcPIF4* genes which are considered negative regulators of the plant branching were more up-regulated in the apical buds of cultivar Campisi compared with the Castellana, suggesting a potential inhibition of apical branches and increased axillary activity (**Table 5**; **Supplementary Figure S1**). The chromosomal location analysis of the major ten and seven genes of chickpea and lentil, respectively, suggested the presence of two branching-associated quantitative trait locus (QTL#1: *CaBAS1* and *CaAGL8* in chromosome 7; and QTL#2: *CaHB53* and *CaCCD2* in chromosome 8) in chickpea (**Supplementary Figure S4A**), while in lentil, all seven genes were located distantly from each other (**Supplementary Figure S4B**). These collective data suggested that several branching-related transcription factors in the chickpea and lentil may be associated with the differential architecture between contrasting cultivars exploited in this study.

Table 5Expression profile of major genes and transcription factors involved in the plant branching regulation in each pairwise comparison for both chickpea and lentil genotypes and tissues.

**Table 5:** 

Chickpea						
Gene name	Gene ID	Transcript ID	BX_vs BA	FX_vs FA	FX_vs BX	FA_vs BA
*CaLOF1*	Ca_16374	XM_004505358	0	0	0	0
*CaEXB1*	Ca_05173	XM_004504650	0	0	-3.21	0
*CaCUC3*	Ca_04804	XM_004500775	0	0	0	0
*CaLAS*	Ca_26425	XM_004515840	0	0	0	0
*CaARR1*	Ca_02989	XM_004508115	0	0	0	0
*CaRAX1*	Ca_17470	XM_004506000	0	0	0	0
*CaROX*	Ca_09396	XM_027332557	0	1.81	3.30	0
*CaREV*	Ca_14560	XM_004505942	0	0	0	0
*CaDRNL*	Ca_18127	XM_004489718	0	0	0	0
*CaSTM*	Ca_00668	XM_004486133	0	0	0	0
*CaCUC2*	Ca_22532	XM_004488689	0	1.11	0	0
*CaBAS1* (N)	Ca_06638	XM_004508479	0	1.66	2.05	0
*CaLOB1*	Ca_04287	XM_004496275	0	0	0.98	0
*CaAS2* (N)	Ca_20200	XM_004511026	0	-0.90	0	0
*CaAS1* (N)	Ca_21130	XM_004492296	0	0	0	0
*CaWUS*	Ca_01974	XM_004512172	0	0	0	0
*CaAGL6*	Ca_06280	XM_004492609	0	0	0	0
*CaAGL8*	Ca_13222	XM_004508599	0	0	-4.29	-3.78
*CaCUC1*	Ca_19144	XM_004489663	0	0	2.08	0
*CaLOF2*	Ca_08179	XM_004493180	0	0	0	0
*CaRAX2*	Ca_00703	XM_004494007	0	0	0	0
*CaRAX3*	Ca_09203	XM_004498879	0	0	0	0
*CaMYB2* (N)	Ca_03535	XM_004495828	0	0	0	0
*CaWOX4*	Ca_19272	XM_004498986	0	-1.32	-0.83	0
*CaEBE*	Ca_01387	XM_004501702	0	0	0	0
*CaERF053*	Ca_14089	XM_004487241	0	0	0	0
*CaBRC2* (N)	Ca_16227	XM_004509983	0	-1.06	-0.99	0
*CaSPL13A* (N)	Ca_05711	XM_004503686	0	-0.80	0	0
*CaSPL13B* (N)	Ca_01426	XM_004501658	0	0	0	0
*CaHB53* (N)	Ca_02070	XM_004511956	2.37	5.04	0	-1.71
*CaHB21* (N)	Ca_12539	XM_004489241	0	1.32	0	-1.00
*CaHB40* (N)	Ca_12720	XM_004502345	0	0.42	0	0
*CaPIF4* (N)	Ca_21576	XM_004499481	0	0	0	0
*CaWRKY72*	Ca_15343	XM_004508711	0	0	0	0
*CaDOF4.2*	Ca_00318	XM_004485743	0	0	0	0
*CabZIP11* (N)	Ca_15397	XM_004500735	0	0	0	0
*CaATH1*	Ca_09180	XM_004498855	0	0	0	0

**Table:** 

Lentil					
Gene name	Gene ID	CmX_vs CmA	CsX_vs CsA	CsX_vs CmX	CsA_vs CmA
*LcLOF1*	Lcu.2RBY.4g049650	0	0	0	0
*LcEXB1*	Lcu.2RBY.4g062520	0	0	0	0
*LcCUC3*	Lcu.2RBY.3g073260	0	0	0	0
*LcLAS*	Lcu.2RBY.6g011840	0	0	0	0
*LcARR1*	Lcu.2RBY.7g070580	0	-0.97	0	0.93
*LcRAX1*	Lcu.2RBY.4g030260	0	0	0	0
*LcROX*	Lcu.2RBY.6g030130	0	0	0	0
*LcREV*	Lcu.2RBY.4g031310	0	0	0	0
*LcDRNL*	Lcu.2RBY.L014690	0	0	0	0
*LcSTM*	Lcu.2RBY.2g010450	0	0	0	0
*LcCUC2*	Lcu.2RBY.2g079890	0	0	0	0
*LcBAS1*	Lcu.2RBY.7g064680	0	0	0	0.97
*LcLOB1*	Lcu.2RBY.2g089890	0	0	0	0
*LcAS2* (N)	Lcu.2RBY.7g017820	0	0	0	0
*LcAS1* (N)	Lcu.2RBY.6g023970	0	0	0	0.49
*LcWUS*	Lcu.2RBY.5g010110	0	0	0	0
*LcAGL6*	Lcu.2RBY.7g014250	0	0	0	0
*LcAGL8*	Lcu.2RBY.2g065300	0	0	0	0
*LcCUC1*	Lcu.2RBY.2g079910	0	0	0	0
*LcLOF2*	Lcu.2RBY.6g045870	0	0.89	1.22	0
*LcRAX2*	Lcu.2RBY.2g091040	0	0	0	0
*LcRAX3*	Lcu.2RBY.5g070630	0	0	0	0
*LcMYB2* (N)	Lcu.2RBY.1g053830	0	0	0	0
*LcWOX4*	Lcu.2RBY.2g020250	0	0	0	0
*LcEBE*	Lcu.2RBY.3g058850	0	0	0	0
*LcERF053*	Lcu.2RBY.2g088350	0	0	0	0
*LcBRC2* (N)	Lcu.2RBY.2g051900	0	0	0	0
*LcSPL13A* (N)	Lcu.2RBY.4g074930	0	0	0	0
*LcSPL13B* (N)	Lcu.2RBY.3g059590	0	0	0	0
*LcHB53* (N)	Lcu.2RBY.5g008250	-3.68	-3.74	2.97	3.03
*LcHB21* (N)	Lcu.2RBY.2g058660	0	-1.19	0	0.92
*LcHB40* (N)	Lcu.2RBY.6g059580	0	0	0	-0.81
*LcPIF4* (N)	Lcu.2RBY.3g017690	0	-1.13	0	0.77
*LcWRKY72*	Lcu.2RBY.7g061480	0	0	0	0
*LcDOF4.2*	Lcu.2RBY.2g004530	0	1.29	0	0
*LcbZIP11* (N)	Lcu.2RBY.4g039970	0	0	1.53	1.37
*LcATHB1*	Lcu.2RBY.5g070290	0	0	0	0

(N), negative regulator of plant branching.

0, statistically non-significant, *p*-value <0.05 and FDR <0.05; BX, Blanco lechoso axillary bud; BA, Blanco lechoso apical bud; FX, FLIP07–318C axillary bud; FA, FLIP07–318C apical bud; CsX, Castellana axillary bud; CsA, Castellana apical bud; CmX, Campisi axillary bud; CmA, Campisi apical bud; Blanco lechoso, little branched; FLIP07–318C, highly branched; Castellana, little branched; and Campisi, highly branched.

### RNA-seq validation by real-time RT-PCR

In order to validate the RNA-seq expression data, five genes of chickpea and five genes of lentil were randomly selected to evaluate the expression profile via real-time RT-PCR in the same tissues and contrasting cultivars. The RNA-seq results were successfully validated by real-time RT-PCR for the five selected genes both in chickpea and lentil. The Pearson correlation coefficient alongside the *p*-values showed that genes had a significant positive correlation supported by *p*-value ≤0.05 (**Supplementary Table S3**), indicating that these genes exhibited equivalent expression patterns between RNA-seq and real-time RT-PCR datasets. The chickpea *CaBES1* (branching-related; Hu et al., 2020), *CaFHY1*, *CaFHY3* and *CaFAR1* (branching-related; Stirnberg et al., 2012; Xie et al., 2020), and *CaDOF4.2* (branching-related; Zou et al., 2012) genes were monitored and revealed accordance for differential expression level between RNA-seq *versus* real-time RT-PCR of 90% (**Supplementary Table S3**; **Supplementary Figures S5**, **S6**). Similarly, lentil *LcFITNESS* (related to broad stress tolerance and improved yield; Osella et al., 2018; Mengarelli et al., 2021), *LcFHY3* and *LcFAR1* (branching-related; Stirnberg et al., 2012; Xie et al., 2020), *LcDOF4.2* (branching-related; Zou et al., 2012), and *LcBS1* (related to seed yield and plant growth; Ge et al., 2016) genes were monitored and also revealed accordance for differential expression level between RNA-seq *versus* real-time RT-PCR of 90% (**Supplementary Table S3**; **Supplementary Figures S5**, **S6**). Therefore, transcript expression data via RNA-seq are supported with high agreement by real-time RT-PCR data.

## Discussion

There is currently a considerable number of chickpea and lentil accessions, genotypes, and cultivars in germplasm banks around the world with enormous genetic and phenotypic variability mainly related to plant architecture (Piergiovanni, 2022). In particular, chickpea and lentil plants with low branching, erect growing stems, high apical dominance, high pod productivity, and high grain yield per plant are desired agronomic characteristics in commercial cultivars (Asati et al., 2022; Mitache et al., 2024). Therefore, significant efforts are still needed in plant breeding and genetic engineering to develop superior cultivars of chickpea and lentil better adapted to mechanized planting and harvesting systems (Singh et al., 2019; Yang et al., 2021). Furthermore, improving plant architecture can impact the grain productivity versus biomass ratio, reduce susceptibility to abiotic and biotic stresses, and increase production and yield per cultivated area (Basso et al., 2024a). In this way, expanding knowledge about the genetic basis associated with the regulation of plant branching can provide biotechnological assets and contribute to the improvement of these crops. In this present study, the global transcript expression profile was evaluated in two contrasting cultivars and two main tissues associated with the modulation of branching in chickpea and lentil plants. For this, the chickpea cultivars Blanco lechoso and FLIP07–318C and the lentil cultivars Castellana and Campisi were previously determined as phenotypically contrasting with each other in terms of branching profile (Basso et al., 2024a). In this sense, the axillary and apical buds were chosen for evaluation, since they are the major tissues involved in the plant branching. In addition, it is important to mention that the fine-tuning between apical and axillary activity are determining factors to regulate cotyledonary branching or apical dominance (Beveridge et al., 2023). This balance is orchestrated by numerous factors, mainly gene expression and hormones, and is led by the signaling coming from the primary shoot apex (Kebrom, 2017; Yuan et al., 2023). For example, if the main apex is removed or its activity reduced, dormant axillary buds below can be activated (Ongaro et al., 2008; Müller & Leyser, 2011). Our RNA-seq study revealed a total of 1,624 and 2,512 differentially expressed transcripts in chickpea and lentil datasets, respectively. Objectively, part of them can be categorized into mechanisms closely associated with the modulation of branching, while the other part is involved secondarily or indirectly in plant branching. Furthermore, it must be considered that many mechanisms are interconnected and act on each other to provide regulatory feedback (Barbier et al., 2019; Salam et al., 2021). In view of this, herein were desiccated the influence of differentially expressed transcripts on the major pathways closely associated with the regulation of chickpea and lentil branching, such as sucrose- and trehalose-6-phosphate-triggered signaling pathways, hormonal balance, auxin, cytokinin and strigolactones signaling pathways, and major transcriptions factors and genes linked to multiple mechanisms. Therefore, the dissection of these major pathways, transcription factors, and genes can provide consolidated data to improve understanding of the mechanisms involved in the branching control of chickpea and lentil and can reveal suitable target genes to be evaluated for the biotechnological potential through transgenesis and genome editing.

### Sucrose-triggered signaling pathway

The proper functioning of essential biological processes are determining factors for plant growth, branching, flowering, and seed production (Julius et al., 2017; Wingler and Henriques, 2022). The tuning of these processes and transitioning to the next phase is finely adjusted and modulated by the influence of good or stressful conditions to which the plants are exposed (Lemoine et al., 2013; Beveridge et al., 2023). In particular, similar to increased auxin concentration in the apical buds, the availability and supply of sugars to meet the demand of the apical meristem and the limitation for axillary buds are some of the main factors that determine apical dominance (Mason et al., 2014). Therefore, the signaling pathway triggered by these sugars such as sucrose, glucose, fructose, and trehalose-6-phosphate contributes to regulating from the developmental stage transitions to plant branching, following source-to-sink flux and linked with hormonal signaling (Wingler, 2017; Barbier et al., 2019; Salam et al., 2021). In particular, sucrose and trehalose-6-phosphate are closely related to plant branching regulation, while glucose and fructose act secondarily on the modulation of plant growth and branching (Figueroa and Lunn, 2016; Barbier et al., 2019). Sucrose is the main sugar since it can be transported by phloem over long distances and may regulate plant branching by directly inducing bud outgrowth, by inhibiting or antagonizing the strigolactones signaling pathway in different steps, or by inducing cytokinin biosynthesis (Lemoine et al., 2013; Salam et al., 2017; Barbier et al., 2019). Overall, the shoot tip growth inhibits axillary bud outgrowth because the shoot tip is a sink for sucrose, depriving axillary buds of sugar (Barbier et al., 2015). Although sucrose acts directly in certain signaling processes, once in the axillary bud or apical meristem, it also leads to trehalose-6-phosphate accumulation and both can inhibit the central growth repressors SnRK1 kinases (Barbier et al., 2019; Fichtner et al., 2021). In this way, both sucrose and trehalose-6-phosphate act on each other to provide feedback under the regulatory pathway (Stein and Granot, 2019). In particular, the sucrose-triggered signaling pathway for branching modulation is mediated mainly by trehalose-6-phosphate and secondly by glucose, fructose, and other intermediate sugars (Miyagawa et al., 2001; Barbier et al., 2015; Otori et al., 2017). In our RNA-seq datasets were identified 19 and 15 main differentially expressed transcripts as involved in sucrose metabolism, transport, signaling, and sensing both in chickpea and lentil, respectively. In particular, among the main differentially expressed genes identified as associated with chickpea and lentil branching modulation are *SWEETs* involved in sugar bidirectional transport (Gautam et al., 2022), *EXL2* involved in sugar sensing (Schröder et al., 2012), *SnRK1* involved in sugar signaling and bud outgrowth inhibition (Barbier et al., 2019), *INTs* involved in inositol transport (Strobl et al., 2018), as well as several other genes involved in sucrose biosynthesis or catabolism, such as, for example, *SIP2* (Peters et al., 2010) and *SuSy* (Stein and Granot, 2019). The transgenic overexpression of the *CmSWEET17* gene promoted axillary bud growth in *Chrysanthemum morifolium* by also inducing up-regulation of several auxin transporter genes (Liu et al., 2019). In turn, *EXL2* (EXORDIUM-like) genes are associated with bud dormancy and are involved in sugar sensing with a role under carbon starvation conditions (Schröder et al., 2012; Tarancón et al., 2017). In the meantime, the SnRK1 kinase complex acts as a central repressor of plant growth and bud dormancy, integrating nutrient status at the cellular level and regulating cell growth arrest in nutrient-limiting conditions (Martín-Fontecha et al., 2018; Barbier et al., 2019). The *SnRK1/KING1* gene was identified as differentially modulated with a positive correlation between expression with branching or apical dominance in both chickpea and lentil, which is a major regulator connecting sucrose metabolism with enzyme activities through the SnRK1 targets (Stefan et al., 2022). Likewise, the inositol transporters encoded by *INT* genes act as H^+^/myo-inositol symporters across the plasma membrane from the vacuole into the cytoplasm and are closely related to cell elongation, plant growth, and branching (Schneider et al., 2006; Strobl et al., 2018). Therefore, these data support that metabolism and sucrose-mediated signaling pathway are positively correlated with enhanced axillary branching or apical dominance in these crops. Similar results were observed in Arabidopsis and tobacco, indicating that carbon partitioning alterations significantly affect shoot branching development (Freixes et al., 2002; Tamoi et al., 2014; Otori et al., 2017).

### Trehalose-6-phosphate-triggered signaling pathway

Trehalose is used as a carbon source and protective compound towards adverse conditions, while its phosphorylated intermediate, trehalose-6-phosphate, is a sugar-signaling metabolite that regulates several biological processes including plant branching (Ponnu et al., 2011; Nunes et al., 2013; Paul et al., 2018). The trehalose-6-phosphate promotes plant branching by inhibiting the activity of SnRK1/KIN10 and SnRK1/KIN11 proteins (Zhang et al., 2009; Wingler and Henriques, 2022; Morales-Herrera et al., 2023). In Arabidopsis, TPS enzymes convert glucose-6-phosphate and UDP-glucose into trehalose-6-phosphate, while trehalose-6-phosphate is dephosphorylated into trehalose by TPP enzymes, and then hydrolyzed by trehalase (TRE1) enzyme into two glucose molecules (Ponnu et al., 2011; Gazzarrini and Tsai, 2014). For instance, the HXK1 enzyme converts glucose into glucose 6-phosphate, which is used by TPS enzymes to produce trehalose-6-phosphate (Barbier et al., 2021). The *TPS* gene overexpression in Arabidopsis increased trehalose and trehalose-6-phosphate levels and resulted in a dehydration tolerance phenotype and delayed flowering (Avonce et al., 2004; Fichtner et al., 2020). Likewise, *TPP* gene overexpression in Arabidopsis improved stress tolerance by accumulating soluble sugar and jasmonic acid and reduced plant branching, while the knockout mutant resulted in drought-sensitive plants (Lin et al., 2019; Fichtner et al., 2021; Lin et al., 2023). In contrast, the overexpression of the *TRE1* gene in Arabidopsis improves drought tolerance (Van Houtte et al., 2013). Herein, the trehalose-6-phosphate pathway was emphasized and a parallel was drawn with the contrasting branching profile of chickpea and lentil cultivars. In particular, our transcript expression data showed up-regulation of some *TPS* genes and suggested a higher trehalose-6-phosphate accumulation in apical buds of highly branched cultivars of chickpea and lentil. However, in apical buds of these cultivars highly branched there was also up-regulation of transcripts coding for the SnRK1 protein that inhibits branching, while down-regulation of transcripts coding for the HXK1 proteins that stimulate branching both in chickpea and lentil. The HXK1 acts as a central sugar-sensing and -signaling protein and is involved in stimulating bud outgrowth, increasing plant branching, and promoting juvenile-to-adult phase transition upstream of cytokinin and strigolactone signaling pathways (Wingler, 2017; Barbier et al., 2021). The *AtHXK1* gene overexpression resulted in Arabidopsis plants without apical dominance and increased emergence of lateral shoots (Kelly et al., 2012), while knockout mutant plants showed decreased cytokinin levels, increased expression of *MAX2* gene, sugar-insensitive phenotype, and reduced growth and branching (Avonce et al., 2004; Barbier et al., 2021; Li et al., 2023). Likewise, the *STP1* gene was finely up-regulated in apical buds of cultivar highly branched of both chickpea and lentil. In particular, the STP1 contributes to the regulation of the genes involved in shoot branching via carbon partitioning in Arabidopsis (Cordoba et al., 2015; Otori et al., 2019). In addition, STP1 is also a regulator of glucose, abscisic acid, and stress signaling (Avonce et al., 2004; Cordoba et al., 2015). The constitutive overexpression of the *STP1* gene reduced plant growth and branching while the knockout mutant plants showed a phenotype similar to the wild-type plants (Otori et al., 2019). Therefore, these collective data revealed that several genes of trehalose-6-phosphate pathway are closely associated with plant branching modulation in chickpea and lentil, and are suggested as suitable targets for branching-directed biotechnological tools.

### Broad hormonal changes

The auxin, cytokinin, and strigolactones are the major hormones involved in plant branching, while other plant hormones such as ABA, JA, and brassinosteroids act indirectly on the modulation of branching and plant growth (Ongaro and Leyser, 2007; Ferguson and Beveridge, 2009). In this context, auxin moves down the dominant shoot and stem to prevent the formation of new buds and branches, while cytokinin promotes meristem activity and bud growth (Müller and Leyser, 2011). In turn, in addition to acting mainly in signaling to plant defense against biotic and abiotic stresses, ABA, salicylic acid, and JA act by inhibiting plant branching (Wasternack, 2015; Yao and Finlayson, 2015; Li et al., 2022). Meanwhile, strigolactones act mainly by regulating branching, which can also be linked to resilience towards stresses (Wang et al., 2015; Wallner et al., 2017; Sun et al., 2022). In turn, brassinosteroids act by promoting an increase in cell volume in the meristem and control multiple processes related to bud outgrowth, branching, and apical dominance (Wei and Li, 2020; Xia et al., 2021). In contrast, ethylene acts mainly in the formation of lateral roots, inhibiting leaf and shoot growth, and regulating plant senescence, while gibberellin acts in seed germination, root and shoot elongation, flowering, fruit patterning, and regulating positively or negatively the axillary bud development (Dubois et al., 2018; Katyayini et al., 2020). In general, all these hormones work in a complex signaling network in a highly interconnected and finely regulated way, depending on the environmental context, plant stage, and plant tissue. Therefore, the action of these hormones in lateral branching and apical dominance is highly complex (Müller and Leyser, 2011; Kebrom, 2017). Herein, it was observed that several transcripts involved in the biosynthesis, signaling, or degradation of all these hormones mentioned above were differentially modulated between apical and axillary buds and contrasting cultivars of chickpea and lentil. In this context, there was less differential modulation of these transcripts in the apical buds of chickpea cultivar lower branched compared to the axillary buds of the same cultivar, with most of these transcripts being involved in the degradation of hormones that inhibit branching. In contrast, there was greater differential modulation of these transcripts in the apical buds of chickpea cultivar highly branched compared to the axillary buds of the same cultivar, with most of these transcripts being involved in the signaling and degradation of different hormones. In the same sense, in the comparison between different tissues and contrasting cultivars of chickpea, several up- or down-regulated transcripts were observed, indicating that there is a significant difference at the hormonal level between these contrasting cultivars of chickpea. In lentil, while there was negative regulation of several of these transcripts involved in the hormonal pathway in the apical buds compared to the axillary buds of the cultivar lower branched, in the cultivar highly branched it was found that there was almost no difference in these transcripts between apical and axillary buds. In the same sense, the number of these transcripts differentially modulated indicated a high difference between contrasting cultivars for both apical and axillary buds. These observations at the hormonal level are in agreement with the fact that multiple pathways regulate bud outgrowth, shoot branching, and apical dominance (Ongaro and Leyser, 2007; Ferguson and Beveridge, 2009; Beveridge et al., 2023). Furthermore, the fact that apical dominance is reduced in cultivars highly branched of chickpea and lentil, there is a tendency for there to be greater hormonal activity in axillary and apical buds (Müller and Leyser, 2011; Cao et al., 2023). Therefore, these collective data revealed that hormonal changes are evident between contrasting cultivars of chickpea and lentil and that there may be key transcripts involved in plant branching and apical dominance of these cultivars.

### Cytokinin and auxin signaling pathways

Until recently, cytokinin and auxin were considered the two major hormones directly involved in modulating apical dominance and stem branching in floral plants, with the hormone strigolactones recently being added to this list (Shimizu-Sato et al., 2009; Weijers and Wagner, 2016). In general, these two first hormones provide regulatory feedback on each other, in addition to each modulating the transcription of several transcription factors and hundreds of genes involved in their pathways (Muller and Leyser, 2011; Yuan et al., 2023). The cytokinin and auxin pathway interactions determine the balanced control of axillary branching and apical dominance since the auxin (indole-3-acetic acid; IAA) produced at the shoot apex translocates through phloem by PIN-FORMED (PIN) transporters, inhibiting isopentenyltransferase (IPT) enzymes and activating cytokinin oxidase/dehydrogenase (CKX) enzymes (Kuroha et al., 2009; Shimizu-Sato et al., 2009; Adamowski and Friml, 2015; Kieber and Schaller, 2018). In consequence, IAA inhibits the accumulation and promotes the degradation of cytokinin in dormant axillary buds, which then results in the inhibition of branching (Shimizu-Sato et al., 2009). In turn, the low or absence of auxin production (*e.g.*, decapitated plants) in the shoot apex no longer exerts this inhibitory effect on cytokinin, releasing IPT and inhibiting CKX enzymes, in this way the dormant axillary buds begin to accumulate cytokinin, consequently triggering branching (Shimizu-Sato et al., 2009; Qiu et al., 2019). Once these axillary buds are transformed into dominant shoots, they produce auxin (IAA), accumulate PIN transporters, and auxin translocation by PIN through the shoot-phloem again leads to inhibition of the cytokinin pathway (Shimizu-Sato et al., 2009; Muller and Leyser, 2011). Other major players are involved in this mechanism triggered by cytokinin to modulate plant branching, such as Arabidopsis histidine kinase (AHK) for cytokinin signal perception (Kumar and Verslues, 2015), LONELY GUY (LOG) for cytokinin biosynthesis (Kuroha et al., 2009), Arabidopsis histidine phosphotransfer proteins (AHPs) for cytokinin signaling (Hutchison et al., 2006), Arabidopsis response regulator proteins (ARRs) for activation of cytokinin response signaling (Zubo et al., 2017), ATP-BINDING CASSETTE G21 (ABCG21) for cytokinin transport (Kim et al., 2020), CYP735A1 for trans-zeatin biosynthesis (Takei et al., 2004), and zeatin o-glucosyltransferase (ZOG) for zeatin degradation (Frébort et al., 2011). Likewise, there are also other major players involved auxin pathway, such as auxin/indole-3-acetic acid (Aux/IAA) for auxin signaling (Overvoorde et al., 2005), YUCCA (YUC) for auxin biosynthesis (Zhao, 2010), IAA-leucine resistant (ILR) for auxin degradation (Hayashi et al., 2021), PIN-LIKES (PILS) and AUXIN1/LIKE-AUX1 (Aux/LAX) for auxin transport (Zhao et al., 2021), and receptor-like transmembrane kinase (TMK) for auxin perception and signaling (Gu et al., 2022).

In our RNA-seq datasets, 15 and 11 main differentially expressed transcripts annotated as involved in cytokinin and auxin signaling pathways of chickpea and lentil, respectively, were identified. These differentially expressed genes play notable roles in hormone perception, signaling, transport, biosynthesis, and degradation, indicating that these expression modulations can contribute to the regulation of axillary branching *versus* apical dominance in contrasting cultivars of these two crops. The high expression levels of *CaCKX3*, *CaAHK1*, *CaLOG3*, *CaAHP1/2/4*, *CaARR1*, *CaABCG21*, *CaCYP735A1* genes, involved in the cytokinin pathway, and *CaAux/IAA2/14*, *CaYUC10*, *CaPIN2*, and *CaILR1* genes, involved in auxin pathway, in axillary buds were associated with higher axillary branching in chickpea. Furthermore, the lower expression levels of *CaLOG3*, *CaAHP1*, *CaAHP4*, *CaPIN2*, *CaILR1*, and *CaAux/IAA2* genes in apical buds were also associated with the reduced apical dominance and higher axillary branching in chickpea. Likewise, the high expression levels of *LcAHK1*, *LcARR12*, and *LcTMK1/3* genes, and lower expression levels of *LcZOG1*, *LcAux/LAX1/2*, and *LcTMK2* genes in axillary buds were associated with higher axillary branching in lentil. Meanwhile, the high expression levels of *LaAHK1*, *LcARR2*, and *LcTMK1/3* genes, and lower expression levels of *LcCYP735A1*, *LcZOG1*, *LcPILS1*, *LcAux/LAX1/2*, *LcTMK2*, and *LcAux/IAA14* genes in apical buds were associated with the reduced apical dominance and higher axillary branching in lentil.

The transgenic overexpression of *AtCKX3* gene resulted in Arabidopsis plants with the phenotype of cytokinin-deficient plants and alteration in plant growth and development compared to wild-type control plants (Dello Ioio et al., 2012). Likewise, the transgenic overexpression of *AtAHK* gene resulted in Arabidopsis plants with altered cytokinin perception and signaling, consequently, showing affected growth and development (Bartrina et al., 2017). Mutant Arabidopsis plants for T-DNA insertion within the *AtLOG3* gene were less sensitive to cytokinin and showed phenotypic changes in plant development (Kuroha et al., 2009). Similarly, mutant Arabidopsis plants for T-DNA insertion within the multiple *AtAHP* genes showed reduced sensitive to cytokinin and altered development phenotype, indicating that these genes act redundantly as positive regulators of cytokinin signaling (Hutchison et al., 2006). The transgenic overexpression of different *AtARR* genes results in Arabidopsis plants with a variety of cytokinin-associated phenotypes (Osakabe et al., 2002; Ren et al., 2009). The *Atabci19*/*abci20*/*abci21* triple and *Atabci20*/*abci21* double knockout Arabidopsis mutants showed hypersensitive to cytokinin and altered plant development, indicating that AtABCG21 acts by fine-tuning the cytokinin response (Kim et al., 2020). The *Jatropha curcas Jccyp735a*-knockout mutant plants generated by genome editing showed retarded plant growth and altered trans-zeatin and trans-zeatin-riboside metabolism and changed cytokinin signaling pathway (Cai et al., 2018). The constitutive overexpression of *ZOG1* gene in transgenic maize and tobacco resulted in cytokinin-deficient plants, growth retardation, delayed senescence, and tasselseed formation (Martin et al., 2001; Rodo et al., 2008).

Meanwhile, transgenic overexpression of different *Aux/IAA* genes caused several auxin-related altered phenotypes in Arabidopsis and rice plants (Sato and Yamamoto, 2008; Song and Xu, 2013). Transgenic overexpression or triple and quadruple knockout mutants of *YUC* genes altered auxin biosynthesis and transport in Arabidopsis and influenced plant growth and development (Cheng et al., 2006; Munguía-Rodríguez et al., 2020). The *AtPIN3* and *AtPIN6* genes overexpression in Arabidopsis and tobacco plants enhanced auxin efflux, promoted auxin unbalance, and altered plant development, branching, and apical dominance (Lee and Cho, 2006; Cazzonelli et al., 2013). Arabidopsis plants with loss-of-function of *ILR* genes showed reduced sensitivity to auxin (Rampey et al., 2006). In contrast, transgenic overexpressing of the *ILR1* gene in tomato plants resulted in several phenotype alterations, including branching and growth of internodes (Wang et al., 2021b). Likewise, transgenic overexpression or loss-of-function assays showed that TMK transmembrane receptors are essential to auxin perception and signaling, and regulate differential growth and apical dominance (Cao et al., 2019; Marquès-Bueno et al., 2021). The transgenic overexpression of different *PILS* genes in Arabidopsis interferes with nuclear auxin signaling and plant growth and development (Sun et al., 2020; Feraru et al., 2022). Thus, these previous studies reveal the functional complexity of these genes identified as differentially expressed in our chickpea and lentil datasets. Furthermore, these studies indicate the narrow possibilities of using these highlighted genes related to cytokinin and auxin pathways in biotechnological tools to modulate the branching of these two crops. Therefore, these collective data indicate that the balance of cytokinin and auxin between axillary and apical buds is a determining factor for the regulation of plant branching in both chickpea and lentil.

### Strigolactones signaling pathway

Strigolactones promote ubiquitination of SCF^MAX2^/D14/SMXL protein complex, which is recognized by the 26S proteasome and directs to degradation, unlocking strigolactone-dependent signal transduction and releasing BRANCHED 1 (BRC1) transcription factor (Zhou et al., 2013; Wang et al., 2015; Bennett et al., 2016). In turn, BRC1-mediated downstream signaling leads to an inhibition of branching, while BRC1 inactivity causes an increased level of branching. Therefore, the presence of strigolactones and BRC1 at higher levels inhibits plant branching. To better understand this signaling pathway, CCD subfamily proteins are major players involved in the strigolactones biosynthesis (Basso et al., 2023), while SMAX/SMXL family proteins are involved in the strigolactone signaling pathway (Basso et al., 2024a), which a part of them is directly linked with the BRC1 transcription factor (Aguilar-Martínez et al., 2007; Bennett et al., 2016; Seale et al., 2017). In this way, BRC1 acts as one of the main players in this signaling pathway modulating the transcriptional activation of several downstream genes involved in plant branching. Also, other secondary partner proteins act as negative regulators of BRC1 and indirectly influence plant branching (Wang et al., 2019). Among them negative regulators, TiE1, LAP1, and BES1 proteins interact and inhibit BRC1, promoting an increase in plant branching (Yang et al., 2018; Diao et al., 2019; Hu et al., 2020; Maurya et al., 2020). In turn, the CARBOXYLESTERASE 15 enzyme (CXE15) acts in the strigolactones catabolism (Xu et al., 2021). Herein, the *CaCCD2* and *CaSMXL7* genes were up-regulated and associated with reduced chickpea branching, while the *CaSMXL2* gene up-regulation in the apical buds was associated with an increase in axillary branching. Meanwhile, the *LcCCD1*, *LcCCD5*, *LcSMXL3*, and *LcSMXL7* genes up-regulation in apical buds was associated with an increased in axillary branching of lentil, and *LcBRC1* gene down-regulation in apical buds was associated with a decreased in axillary branching. Therefore, several chickpea and lentil genes of the strigolactones pathway are potentially involved in the modulation of plant branching and suggested as targets for tissue-specific modulation via transgenesis with tissue-specific promoters and gene knockout using genome editing tools. Previous studies showed that the transgenic overexpression of some *CCD* genes resulted in reduced plant branching while gene knockout increased plant branching, in particular, *CDD* genes involved in strigolactones biosynthesis (Snowden et al., 2005; Ren et al., 2020; Wang et al., 2021a; Hao et al., 2023). On the other hand, the *CaMXL2* and *LcSMXL3* genes based on orthologue analysis were previously suggested as involved in the phloem formation independently from strigolactone signaling, while *CaSMXL7* and *LcSMXL7* genes were suggested as involved in the regulation of shoot branching and elongation (Basso et al., 2024a). Mutant plants for these *SMXL* genes involved in the strigolactones- and karrikins-independent pathway showed poor phloem formation, altered sugar accumulation, and seedling lethality (Wallner et al., 2017; Hardtke, 2023; Wallner et al., 2023). As already mentioned, the degradation of the complexed SMXL6,7,8 proteins mediated by strigolactones leads to the activation of the BRC1 signaling pathway to inhibit plant branching (Wang et al., 2015). The overexpression or knockout of the *SMXL7* gene has been shown to alter the number and growth of branches in Arabidopsis (Liang et al., 2016). In addition, SMXL7 was also shown as a transcription suppressor in Arabidopsis by binding to SnRK2.3 and SnRK2.6 promoters, which are positively involved in ABA-mediated response to drought stress (Korek and Marzec, 2023; Lian et al., 2023). Similarly, the BRC1-mutant plants displayed a higher number of branches (Aguilar-Martínez et al., 2007; González-Grandío et al., 2013), while *BRC1* gene overexpression in transgenic lines resulted in plants with reduced branching (Ding et al., 2020; Maurya et al., 2020; Min et al., 2021). Therefore, several leading candidate genes of the strigolactones signaling pathway were highlighted for further use in genetic engineering to improve chickpea and lentil architecture.

### Branching-related transcription factors and major proteins

Several major effect transcription factors and proteins have already been identified as involved in the positive or negative regulation of plant branching (Zhang et al., 2022; Yang et al., 2023). Among these, a group of 16 highly interconnected members, as well as other notable members involved in branching, were monitored in this study. Among these members, the CaEXB1, CaBAS1, CaAGL8, CaWOX4, CaHB21, CaHB40, and CaHB53 proteins were identified as associated with differential branching between contrasting cultivars of chickpea. Similarly, the LcLOF2, LcAS1, LcHB53, and LcPIF4 proteins were also identified as associated with plant branching between contrasting cultivars of lentil. However, these transcription factors have not yet been functionally characterized in chickpea and lentil, but their orthologues in Arabidopsis have been extensively studied. In particular, the EXB1 protein is a WRKY transcription factor that positively regulates the shoot branching by transcriptionally modulating *RAX* genes in Arabidopsis (Guo et al., 2015). The RNAi-mediated down-regulation of EXB1 resulted in Arabidopsis plants with fewer branches, while the transgenic overexpression resulted in increased branching (Guo et al., 2015; Yu et al., 2016). In addition, EXB1 was shown as modulated by abiotic stress conditions (Guo and Qin, 2016; Yu et al., 2017). Meanwhile, BAS1 is an enzyme modulated by auxin with capacity of inactivate brassinosteroids, which is up-regulated by LOB1 to accumulate low levels of brassinosteroids and reduce cell volume in the boundary zone and, consequently, regulate hypocotyl elongation and plant branching (Neff et al., 1999; Turk et al., 2005; Youn et al., 2016), while LOB1 transcription is modulated by brassinosteroids in Arabidopsis (Bell et al., 2012; Gendron et al., 2012). The negative modulation of brassinosteroid levels resulted in plants with typical brassinosteroid-deficient phenotypes (Han et al., 2017). In contrast, AGL8 (also known as FRUITFULL) is an Agamous-like MADS-box protein accumulated in apical meristems, negatively modulated by APETALA1 (formerly known as AGL7), which acts by regulating the transition between vegetative phase to reproductive phase, cell differentiation during Arabidopsis fruit development, and inflorescence architecture (Mandel and Yanofsky, 1995; Gu et al., 1998; Ferrándiz et al., 2000; Melzer et al., 2008; Paull et al., 2023). In turn, APETALA1 regulates the expression of several genes involved in floral development and plant branching (Winter et al., 2015; Goslin et al., 2017). In this context, AGL8 controls *SAUR10* gene expression to regulate Arabidopsis growth and architecture, and AGL8 overexpression or knockout significantly alters plant architecture (Bemer et al., 2017; Führer et al., 2020).

The WOX4 is a WUSCHEL-related HOMEOBOX protein that regulates the cell division and stem cell maintenance in procambium/cambium (Hirakawa et al., 2010; Nakata et al., 2012; Dolzblasz et al., 2016; Kucukoglu et al., 2017). The *WOX4* gene expression is down-regulated by the BES1 transcription factor, which develops antagonistic roles in shoot branching and cambium differentiation linked by the strigolactones signaling pathway (Hu et al., 2021). The RNAi-mediated down-regulation of the *WOX4* gene resulted in Arabidopsis plants with reduced vascular development and overaccumulate undifferentiated ground tissue, while the overexpression conferred a hypervascularization phenotype in tomato plants (Ji et al., 2009; Zhang et al., 2019). The *Malus domestica WOX4–2* gene overexpression significantly enhanced adventitious shoots in transgenic tobacco and regulated adventitious shoot regeneration in transgenic apple trees (Dong et al., 2022b). Meanwhile, the *HB21*, *HB40*, and *HB53* genes act redundantly as Homeobox transcription factors to inhibit branching and are positively regulated transcriptionally by BRC1 and SMAX1 (Zheng et al., 2021; Dun et al., 2023; van Es et al., 2024). They are expressed in axillary buds and in stomata guard cells and enhanced by low R:FR light, repress shoot branching, and directly co-regulate *NCED3* gene expression and ABA levels in Arabidopsis buds (O’Malley et al., 2016; González-Grandío et al., 2017). In this context, Arabidopsis plants with different combinations of mutants of these four genes showed a high number of axillary buds and longer hypocotyls (González-Grandío et al., 2017; Dong et al., 2022a; Sánchez-Gerschon et al., 2023).

The LOF2 is a LATERAL ORGAN FUSION transcription factor of the MYB family, positively transcriptionally regulated by the auxin transporter ABCB19 at the boundaries of lateral organs, that acts in the separation of lateral organ and axillary shoots, and initiation of axillary meristem in Arabidopsis and tomato (Lee et al., 2009; Naz et al., 2013; Zhao et al., 2013). The *LOF2* gene has a high sequence identity and is closely related to *LOF1*, both share redundant functions. The *lof1/lof2* double mutant plants have stronger defects in axillary meristem formation and organ separation (Lee et al., 2009), while the LOF gene overexpression resulted in dwarfed Arabidopsis plants (Gomez et al., 2011). Similarly, AS1 is an ASYMMETRIC LEAVES transcription factor of the MYB (SANT) family, that accumulates around vascular tissues in cotyledonary and leaf primordia, and in developing leaves, and acts in leaf development and negative regulation of branching in Arabidopsis (Byrne et al., 2000; Sun et al., 2002; Ikezaki et al., 2010). The AS1 and AS2 proteins bind to promoter regions and repress the *KNOXI* gene family, both involved in plant branching regulation (Guo et al., 2008; Lodha et al., 2013). The *as1* mutant plants exhibit severe pleiotropic phenotypes, in particular, elevated frequency of adventitious shoot formation (Semiarti et al., 2001; Xu et al., 2003; Ikezaki et al., 2010; Husbands et al., 2015). The *AS1* gene overexpression resulted in the formation of narrower and more elongated leaves, and a greater number (Theodoris et al., 2003). In turn, the PIF4 is a PHYTOCHROME-INTERACTING FACTOR transcription factor of the bHLH family that acts to regulate microtubule organization to mediate high temperature-induced hypocotyl cell elongation in Arabidopsis (Zhou et al., 2023). In addition, PIF4 together with PIF5 also regulates axillary branching via bud abscisic acid and stem auxin signaling, and induces dark- and stress-induced senescence in Arabidopsis, but is also negatively regulated by ELF3 and CRY1 (Sakuraba et al., 2014; Holalu et al., 2020; Zhai et al., 2020; Li et al., 2021, 2024). The *pif4*/*pif5* mutant plants exhibit delayed senescence while *PIF4* gene overexpression promotes leaf senescence and increases branching (Sakuraba et al., 2014; Zhai et al., 2020; Li et al., 2021). Therefore, these collective data based mainly on functional analysis of orthologs in Arabidopsis revealed several leading candidate genes for use in genetic engineering from transgenesis or genome editing aimed at improving chickpea and lentil architecture. Moreover, two putative branching-associated QTLs were suggested to occur in chickpea.

## Conclusion

In this study, the global transcript expression profile of two contrasting chickpea and lentil cultivars with plant architecture phenotype of little *versus* highly branched was revealed. A total of 1,624 and 2,512 transcripts were identified as differentially expressed between apical and axillary tissues and different contrasting cultivars of chickpea and lentil, respectively. These differentially expressed transcript sets were responsible for modulating several biological processes such as cell cycle, DNA transcription, energy metabolism, broad hormonal biosynthesis and signaling, proteolysis, and vegetative development between different tissues and contrasting cultivars of chickpea and lentil. In particular, the *CaEXL2*, *CaSnRK1/KING1*, *CaCCD2*, *CaSMXL2*, *CaSMXL7*, *CaEXB1*, *CaBAS1*, *CaAGL8*, *CaWOX4*, *CaHB21*, *CaHB40*, and *CaHB53* genes in chickpea, and *LcEXL2*, *LcSnRK1/KING1*, *LcSMXL7*, *LcBRC1*, *LcLOF2*, *LcAS1*, *LcHB21*, *LcHB53*, and *LcPIF4* genes in lentil were considered as main players involved in differentially regulate the plant branching between contrasting cultivars. Therefore, since each plant species has a particular and multi-mechanistic regulation at the level of gene expression and function associated with branching modulation (Guo et al., 2020), these collective data will contribute to understanding the general molecular mechanism that modulates branching in the chickpea and lentil. Furthermore, several putative high-effect genes associated with the chickpea and lentil branching are highlighted as potential targets for manipulation through genome editing and transgenesis aiming to improve plant architecture.

## Data availability statement

The datasets presented in this study can be found in online repositories. The names of the repository/repositories and accession number(s) can be found below: EMBL-EBI ArrayExpress database under the accession number E-MTAB-13679.

## Author contributions

MBa: Writing – original draft, Methodology, Investigation, Conceptualization. GG: Writing – review & editing, Investigation, Formal Analysis. CV: Writing – review & editing, Investigation. MBu: Writing – review & editing, Supervision, Data curation. FM: Writing – review & editing, Supervision, Project administration, Funding acquisition.

## References

[B1] Adamowski M. Friml J. (2015). PIN-dependent auxin transport: action, regulation, and evolution. Plant Cell 27, 20–32. doi: 10.1105/tpc.114.134874 25604445 PMC4330589

[B2] Aguilar-Martínez J. A. Poza-Carrión C. Cubas P. (2007). Arabidopsis BRANCHED1 acts as an integrator of branching signals within axillary buds. Plant Cell 19, 458–472. doi: 10.1105/tpc.106.048934 17307924 PMC1867329

[B3] Andrews S. (2010) FastQC: A quality control tool for high throughput sequence data. Available online at: http://www.bioinformatics.babraham.ac.uk/projects/fastqc/.

[B4] Arif A. Parveen N. Waheed M. Q. Atif R. M. Waqar I. Shah T. M. (2021). A comparative study for assessing the drought-tolerance of chickpea under varying natural growth environments. Front. Plant Sci. 11. doi: 10.3389/fpls.2020.607869 PMC7928316 33679816

[B5] Asati R. Tripathi M. K. Tiwari S. Yadav R. K. Tripathi N. (2022). Molecular breeding and drought tolerance in chickpea. Life 12, 1846. doi: 10.3390/life12111846 36430981 PMC9698494

[B6] Avonce N. Leyman B. Mascorro-Gallardo J. O. Van Dijck P. Thevelein J. M. Iturriaga G. (2004). The Arabidopsis trehalose-6-P synthase *AtTPS1* gene is a regulator of glucose, abscisic acid, and stress signaling. Plant Physiol. 136, 3649–3659. doi: 10.1104/pp.104.052084 15516499 PMC527163

[B7] Barbier F. F. Cao D. Fichtner F. Weiste C. Perez-Garcia M.-D. Caradeuc M.. (2021). HEXOKINASE1 signalling promotes shoot branching and interacts with cytokinin and strigolactone pathways. New Phytol. 231, 1088–1104. doi: 10.1111/nph.17427 33909299

[B8] Barbier F. F. Dun E. A. Kerr S. C. Chabikwa T. G. Beveridge C. A. (2019). An update on the signals controlling shoot branching. Trends Plant Sci. 24, 220–236. doi: 10.1016/j.tplants.2018.12.001 30797425

[B9] Barbier F. F. Lunn J. E. Beveridge C. A. (2015). Ready, steady, go! A sugar hit starts the race to shoot branching. Curr. Opin. Plant Biol. 25, 39–45. doi: 10.1016/j.pbi.2015.04.004 25938609

[B10] Bartrina I. Jensen H. Novák O. Strnad M. Werner T. Schmülling T. (2017). Gain-of-function mutants of the cytokinin receptors AHK2 and AHK3 regulate plant organ size, flowering time and plant longevity. Plant Physiol. 173 (3), 1783–1797. doi: 10.1104/pp.16.01903 PMC5338655 28096190

[B11] Basso M. F. Arraes F. B. M. Grossi-de-Sa M. Moreira V. J. V. Alves-Ferreira M. Grossi-de-Sa M. F. (2020). Insights into genetic and molecular elements for transgenic crop development. Front. Plant Sci. 11. doi: 10.3389/fpls.2020.00509 PMC7243915 32499796

[B12] Basso M. F. Contaldi F. Lo Celso F. Baratto C. Grossi-de-Sa M. F. Barone G.. (2024a). Identification and expression profile of the *SMAX/SMXL* family genes in chickpea and lentil provide important players of biotechnological interest involved in plant branching. Planta 259, 1–23. doi: 10.1007/s00425-023-04277-y PMC10651550 37966555

[B13] Basso M. F. Contaldi F. Lo Celso F. Karalija E. Paz-Carrasco L. Barone G.. (2023). Expression profile of the *NCED/CCD* genes in chickpea and lentil during abiotic stress reveals a positive correlation with increased plant tolerance. Plant Sci. 336, 111817. doi: 10.1016/j.plantsci.2023.111817 37562731

[B14] Basso M. F. Ferreira P. C. G. Kobayashi A. K. Harmon F. G. Nepomuceno A. L. Molinari H. B. C.. (2019). MicroRNAs and new biotechnological tools for its modulation and improving stress tolerance in plants. Plant Biotechnol. J. 17, 1482–1500. doi: 10.1111/pbi.13116 30947398 PMC6662102

[B15] Basso M. F. Neves M. F. Grossi-de-Sa M. F. (2024b). Agriculture evolution, sustainability and trends, focusing on Brazilian agribusiness: a review. Front. Sustain. Food Syst. 7. doi: 10.3389/fsufs.2023.1296337

[B16] Bell E. M. Lin W. C. Husbands A. Y. Yu L. Jaganatha V. Jablonska B.. (2012). Arabidopsis LATERAL ORGAN BOUNDARIES negatively regulates brassinosteroid accumulation to limit growth in organ boundaries. PNAS 109, 21146–21151. doi: 10.1073/pnas.1210789109 23213252 PMC3529045

[B17] Bemer M. van Mourik H. Muiño J. M. Ferrándiz C. Kaufmann K. Angenent G. C. (2017). FRUITFULL controls SAUR10 expression and regulates Arabidopsis growth and architecture. J. Exp. Bot. 68, 3391–3403. doi: 10.1093/jxb/erx184 28586421 PMC5853401

[B18] Bennett T. Liang Y. Seale M. Ward S. Müller D. Leyser O. (2016). Strigolactone regulates shoot development through a core signalling pathway. Biol. Open 5, 1806–1820. doi: 10.1242/bio.021402 27793831 PMC5200909

[B19] Beveridge C. A. Rameau C. Wijerathna-Yapa A. (2023). Lessons from a century of apical dominance research. J. Exp. Bot. 74, 3903–3922. doi: 10.1093/jxb/erad137 37076257 PMC10400159

[B20] Bolger A. M. Lohse M. Usadel B. (2014). Trimmomatic: a flexible trimmer for Illumina sequence data. Bioinformatics 30, 2114–2120. doi: 10.1093/bioinformatics/btu170 24695404 PMC4103590

[B21] Bu D. Luo H. Huo P. Wang Z. Zhang S. He Z.. (2021). KOBAS-i: intelligent prioritization and exploratory visualization of biological functions for gene enrichment analysis. Nucleic Acids Res. 49, W317–W325. doi: 10.1093/nar/gkab447 34086934 PMC8265193

[B22] Byrne M. E. Barley R. Curtis M. Arroyo J. M. Dunham M. Hudson A.. (2000). Asymmetric leaves1 mediates leaf patterning and stem cell function in Arabidopsis. Nature 408, 967–971. doi: 10.1038/35050091 11140682

[B23] Cai L. Zhang L. Fu Q. Xu Z. F. (2018). Identification and expression analysis of cytokinin metabolic genes IPTs, CYP735A and CKXs in the biofuel plant *Jatropha curcas* . PeerJ 6, e4812. doi: 10.7717/peerj.4812 29785355 PMC5960259

[B24] Cao D. Chabikwa T. Barbier F. Dun E. A. Fichtner F. Dong L.. (2023). Auxin-independent effects of apical dominance induce changes in phytohormones correlated with bud outgrowth. Plant Physiol. 192, 1420–1434. doi: 10.1093/plphys/kiad034 36690819 PMC10231355

[B25] Cao M. Chen R. Li P. Yu Y. Zheng R. Ge D.. (2019). TMK1-mediated auxin signalling regulates differential growth of the apical hook. Nature 568, 240–243. doi: 10.1038/s41586-019-1069-7 30944466

[B26] Cazzonelli C. I. Vanstraelen M. Simon S. Yin K. Carron-Arthur A. Nisar N.. (2013). Role of the Arabidopsis PIN6 auxin transporter in auxin homeostasis and auxin-mediated development. PloS One 8, e70069. doi: 10.1371/journal.pone.0070069 23922907 PMC3726503

[B27] Cheng Y. Dai X. Zhao Y. (2006). Auxin biosynthesis by the YUCCA flavin monooxygenases controls the formation of floral organs and vascular tissues in Arabidopsis. Genes Dev. 20, 1790–1799. doi: 10.1101/gad.1415106 16818609 PMC1522075

[B28] Cici S.-Z.-H. Adkins S. Hanan J. (2008). A canopy architectural model to study the competitive ability of chickpea with Sowthistle. Ann. Bot. 101, 1311–1318. doi: 10.1093/aob/mcn040 18375962 PMC2710251

[B29] Cordoba E. Aceves-Zamudio D. L. Hernández-Bernal A. F. Ramos-Vega M. León P. (2015). Sugar regulation of SUGAR TRANSPORTER PROTEIN 1 (STP1) expression in *Arabidopsis thaliana* . J. Exp. Bot. 66, 147–159. doi: 10.1093/jxb/eru394 25281700 PMC4265152

[B30] Dello Ioio R. Galinha C. Fletcher A. G. Grigg S. P. Molnar A. Willemsen V.. (2012). A PHABULOSA/cytokinin feedback loop controls root growth in Arabidopsis. Curr. Biol. 22, 1699–1704. doi: 10.1016/j.cub.2012.07.005 22902752

[B31] DennisG.ShermanB. T.HosackD. A.YangJ.GaoW.LaneH. C.. (2003). DAVID: database for annotation, visualization, and integrated discovery. Genome Biol. 4, R60. doi: 10.1186/gb-2003-4-9-r60 12734009

[B32] Diao Y. Zhan J. Zhao Y. Liu L. Liu P. Wei X.. (2019). GhTIE1 regulates branching through modulating the transcriptional activity of TCPs in cotton and Arabidopsis. Front. Plant Sci. 10. doi: 10.3389/fpls.2019.01348 PMC6827420 31719830

[B33] Ding N. Qin Q. Wu X. Miller R. Zaitlin D. Li D.. (2020). Antagonistic regulation of axillary bud outgrowth by the BRANCHED genes in tobacco. Plant Mol. Biol. 103, 185–196. doi: 10.1007/s11103-020-00983-3 32124178

[B34] Dolzblasz A. Nardmann J. Clerici E. Causier B. van der Graaff E. Chen J.. (2016). Stem cell regulation by Arabidopsis WOX genes. Mol. Plant 9, 1028–1039. doi: 10.1016/j.molp.2016.04.007 27109605

[B35] Dong S. Tarkowska D. Sedaghatmehr M. Welsch M. Gupta S. Mueller-Roeber B.. (2022a). The HB40-JUB1 transcriptional regulatory network controls gibberellin homeostasis in Arabidopsis. Mol. Plant 15, 322–339. doi: 10.1016/j.molp.2021.10.007 34728415

[B36] Dong H. Zheng Q. Zhou Y. Zhou Y. Bao Z. Lan Q.. (2022b). MdWOX4–2 modulated *MdLBD41* functioning in adventitious shoot of apple (*Malus domestica*). Plant Physiol. Biochem. 186, 11–18. doi: 10.1016/j.plaphy.2022.06.026 35797915

[B37] Dubois M. Van den Broeck L. Inzé D. (2018). The pivotal role of ethylene in plant growth. Trends Plant Sci. 23, 311–323. doi: 10.1016/j.tplants.2018.01.003 29428350 PMC5890734

[B38] Dun E. A. Brewer P. B. Gillam E. M. J. Beveridge C. A. (2023). Strigolactones and shoot branching: What is the real hormone and how does it work? Plant Cell Physiol. 64, 967–983. doi: 10.1093/pcp/pcad088 37526426 PMC10504579

[B39] Feraru E. Feraru M. I. Moulinier-Anzola J. Schwihla M. Santos J. F. S. Sun L.. (2022). ) PILS proteins provide a homeostatic feedback on auxin signaling output. Development 149, dev200929. doi: 10.1242/dev.200929 35819066 PMC9340555

[B40] Ferguson B. J. Beveridge C. A. (2009). Roles for auxin, cytokinin, and strigolactone in regulating shoot branching. Plant Physiol. 149, 1929–1944. doi: 10.1104/pp.109.135475 19218361 PMC2663762

[B41] Ferrándiz C. Gu Q. Martienssen R. Yanofsky M. F. (2000). Redundant regulation of meristem identity and plant architecture by FRUITFULL, APETALA1 and CAULIFLOWER. Development 127, 725–734. doi: 10.1242/dev.127.4.725 10648231

[B42] Fichtner F. Barbier F. F. Annunziata M. G. Feil R. Olas J. J. Mueller-Roeber B.. (2021). Regulation of shoot branching in Arabidopsis by trehalose 6-phosphate. New Phytol. 229, 2135–2151. doi: 10.1111/nph.17006 33068448

[B43] Fichtner F. Olas J. J. Feil R. Watanabe M. Krause U. Hoefgen R.. (2020). Functional features of TREHALOSE-6-PHOSPHATE SYNTHASE1, an essential enzyme in Arabidopsis. Plant Cell 32, 1949–1972. doi: 10.1105/tpc.19.00837 32276986 PMC7268806

[B44] Figueroa C. M. Lunn J. E. (2016). A tale of two sugars: Trehalose 6-phosphate and sucrose. Plant Physiol. 172, 7–27. doi: 10.1104/pp.16.00417 27482078 PMC5074632

[B45] Frébort I. Kowalska M. Hluska T. Frébortová J. Galuszka P. (2011). Evolution of cytokinin biosynthesis and degradation. J. Exp. Bot. 62, 2431–2452. doi: 10.1093/jxb/err004 21321050

[B46] Freixes S. Thibaud M. C. Tardieu F. Muller B. (2002). Root elongation and branching is related to local hexose concentration in *Arabidopsis thaliana* seedlings. Plant Cell Environ. 25, 1357–1366. doi: 10.1046/j.1365-3040.2002.00912.x

[B47] Führer M. Gaidora A. Venhuizen P. Dobrogojski J. Béziat C. Feraru M. I.. (2020). FRUITFULL is a repressor of apical hook opening in *Arabidopsis thaliana* . Int. J. Mol. Sci. 21, 6438. doi: 10.3390/ijms21176438 32899394 PMC7504503

[B48] Gautam T. Dutta M. Jaiswal V. Zinta G. Gahlaut V. Kumar S. (2022). Emerging roles of SWEET sugar transporters in plant development and abiotic stress responses. Cells 11, 1303. doi: 10.3390/cells11081303 35455982 PMC9031177

[B49] Gazzarrini S. Tsai A. Y.-L. (2014). Trehalose-6-phosphate and SnRK1 kinases in plant development and signaling: the emerging picture. Front. Plant Sci. 5. doi: 10.3389/fpls.2014.00119 PMC3978363 24744765

[B50] Ge L. Yu J. Wang H. Luth D. Bai G. Wang K.. (2016). Increasing seed size and quality by manipulating BIG SEEDS1 in legume species. PNAS 113, 12414–12419. doi: 10.1073/pnas.1611763113 27791139 PMC5098654

[B51] Gendron J. M. Liu J.-S. Fan M. Bai M.-Y. Wenkel S. Springer P. S.. (2012). Brassinosteroids regulate organ boundary formation in the shoot apical meristem of Arabidopsis. PNAS 109, 21152–21157. doi: 10.1073/pnas.1210799110 23213257 PMC3529081

[B52] Gomez M. D. Urbez C. Perez-Amador M. A. Carbonell J. (2011). Characterization of constricted fruit (ctf) mutant uncovers a role for AtMYB117/LOF1 in ovule and fruit development in *Arabidopsis thaliana* . PloS One 6, e18760. doi: 10.1371/journal.pone.0018760 21533201 PMC3076444

[B53] González-Grandío E. Pajoro A. Franco-Zorrilla J. M. Tarancón C. Immink R. G. Cubas P. (2017). Abscisic acid signaling is controlled by a BRANCHED1/HD-ZIP I cascade in Arabidopsis axillary buds. PNAS 114, E245–e254. doi: 10.1073/pnas.1613199114 28028241 PMC5240681

[B54] González-Grandío E. Poza-Carrión C. Sorzano C. O. Cubas P. (2013). BRANCHED1 promotes axillary bud dormancy in response to shade in Arabidopsis. Plant Cell 25, 834–850. doi: 10.1105/tpc.112.108480 23524661 PMC3634692

[B55] Goslin K. Zheng B. Serrano-Mislata A. Rae L. Ryan P. T. Kwaśniewska K.. (2017). Transcription factor interplay between LEAFY and APETALA1/CAULIFLOWER during floral initiation. Plant Physiol. 174, 1097–1109. doi: 10.1104/pp.17.00098 28385730 PMC5462026

[B56] Grossi-de-Sa M. F. Basso M. F. (2024). “Ciências agrárias e as revoluções na produção de alimentos: Do passado ao futuro, Chapter 8,” in Segurança alimentar e nutricional: O papel da ciência brasileira no combate à fome, vol. 1 . Ed. Hungria M. H. (Academia Brasileira de Ciências), 48–55. 170 p. Available at: https://www.abc.org.br/wp-content/uploads/2024/03/Seguranca-Alimentar-e-Nutricional-O-Papel-da-Ciencia-Brasileira-no-Combate-a-Fome-LIVRO-ABC-2024.pdf.

[B57] Gu B. Dong H. Smith C. Cui G. Li Y. Bevan M. W. (2022). Modulation of receptor-like transmembrane kinase 1 nuclear localization by DA1 peptidases in Arabidopsis. PNAS 119, e2205757119. doi: 10.1073/pnas.2205757119 36161927 PMC9546594

[B58] Gu Q. Ferrándiz C. Yanofsky M. F. Martienssen R. (1998). The FRUITFULL MADS-box gene mediates cell differentiation during Arabidopsis fruit development. Development 125, 1509–1517. doi: 10.1242/dev.125.8.1509 9502732

[B59] Guo W. Chen L. Herrera-Estrella L. Cao D. Tran L.-S. P. (2020). Altering plant architecture to improve performance and resistance. Trends Plant Sci. 25, 1154–1170. doi: 10.1016/j.tplants.2020.05.009 32595089

[B60] Guo D. Qin G. (2016). EXB1/WRKY71 transcription factor regulates both shoot branching and responses to abiotic stresses. Plant Signaling Behav. 11, e1150404. doi: 10.1080/15592324.2016.1150404 PMC4883898 26914912

[B61] Guo M. Thomas J. Collins G. Timmermans M. C. (2008). Direct repression of KNOX loci by the ASYMMETRIC LEAVES1 complex of Arabidopsis. Plant Cell 20, 48–58. doi: 10.1105/tpc.107.056127 18203921 PMC2254922

[B62] Guo D. Zhang J. Wang X. Han X. Wei B. Wang J.. (2015). The WRKY transcription factor WRKY71/EXB1 controls shoot branching by transcriptionally regulating RAX genes in Arabidopsis. Plant Cell 27, 3112–3127. doi: 10.1105/tpc.15.00829 26578700 PMC4682308

[B63] Haile T. A. Stonehouse R. Weller J. L. Bett K. E. (2021). Genetic basis for lentil adaptation to summer cropping in northern temperate environments. Plant Genome 14, e20144. doi: 10.1002/tpg2.20144 34643336 PMC12893650

[B64] Hallé F. Oldeman R. A. (1970). “Essai sur l’architecture et la dynamique de croissance des arbres tropicaux,” in Collection de Monographies de Botanique et de Biologie Végétale, vol. 6. (Masson, Paris), 25–21. 140 p.

[B65] Han Y. J. Kim Y. S. Hwang O. J. Roh J. Ganguly K. Kim S. K.. (2017). Overexpression of *Arabidopsis thaliana* brassinosteroid-related acyltransferase 1 gene induces brassinosteroid-deficient phenotypes in creeping bentgrass. PloS One 12, e0187378. doi: 10.1371/journal.pone.0187378 29084267 PMC5662239

[B66] Hao J. Yang Y. Futrell S. Kelly E. A. Lorts C. M. Nebie B.. (2023). CRISPR/Cas9-mediated mutagenesis of *Carotenoid Cleavage Dioxygenase* (CCD) genes in sorghum alters strigolactone biosynthesis and plant biotic interactions. Phytobiomes J. 7, 339–351. doi: 10.1094/PBIOMES-08-22-0053-R

[B67] Hardtke C. S. (2023). Phloem development. New Phytol. 239, 852–867. doi: 10.1111/nph.19003 37243530

[B68] Hayashi K. I. Arai K. Aoi Y. Tanaka Y. Hira H. Guo R.. (2021). The main oxidative inactivation pathway of the plant hormone auxin. Nat. Communication 12, 6752. doi: 10.1038/s41467-021-27020-1 PMC8608799 34811366

[B69] Hirakawa Y. Kondo Y. Fukuda H. (2010). TDIF peptide signaling regulates vascular stem cell proliferation via the WOX4 homeobox gene in Arabidopsis. Plant Cell 22, 2618–2629. doi: 10.1105/tpc.110.076083 20729381 PMC2947162

[B70] Holalu S. V. Reddy S. K. Blackman B. K. Finlayson S. A. (2020). Phytochrome interacting factors 4 and 5 regulate axillary branching via bud abscisic acid and stem auxin signalling. Plant Cell Environ. 43, 2224–2238. doi: 10.1111/pce.13824 32542798

[B71] Hu J. Hu X. Yang Y. He C. Hu J. Wang X. (2021). Strigolactone signaling regulates cambial activity through repression of WOX4 by transcription factor BES1. Plant Physiol. 188, 255–267. doi: 10.1093/plphys/kiab487 PMC8774819 34687296

[B72] Hu J. Ji Y. Hu X. Sun S. Wang X. (2020). BES1 functions as the co-regulator of D53-like SMXLs to inhibit BRC1 expression in strigolactone-regulated shoot branching in Arabidopsis. Plant Commun. 1, 100014. doi: 10.1016/j.xplc.2019.100014 33404550 PMC7748003

[B73] Husbands A. Y. Benkovics A. H. Nogueira F. T. S. Lodha M. Timmermans M. C. P. (2015). The ASYMMETRIC LEAVES complex employs multiple modes of regulation to affect adaxial-abaxial patterning and leaf complexity. Plant Cell 27, 3321–3335. doi: 10.1105/tpc.15.00454 26589551 PMC4707451

[B74] Hutchison C. E. Li J. Argueso C. Gonzalez M. Lee E. Lewis M. W.. (2006). The Arabidopsis histidine phosphotransfer proteins are redundant positive regulators of cytokinin signaling. Plant Cell 18, 3073–3087. doi: 10.1105/tpc.106.045674 17122069 PMC1693944

[B75] Ikezaki M. Kojima M. Sakakibara H. Kojima S. Ueno Y. Machida C.. (2010). Genetic networks regulated by ASYMMETRIC LEAVES1 (AS1) and AS2 in leaf development in *Arabidopsis thaliana*: KNOX genes control five morphological events. Plant J. 61, 70–82. doi: 10.1111/tpj.2009.61.issue-1 19891706

[B76] Ji J. Strable J. Shimizu R. Koenig D. Sinha N. Scanlon M. J. (2009). WOX4 promotes procambial development. Plant Physiol. 152, 1346–1356. doi: 10.1104/pp.109.149641 20044450 PMC2832261

[B77] Jiangtao C. Yingzhen K. Qian W. Yuhe S. Daping G. Jing L.. (2015). MapGene2Chrom, a tool to draw gene physical map based on Perl and SVG languages. Yi Chuan = Hereditas 37, 91–97. doi: 10.16288/j.yczz.2015.01.013 25608819

[B78] Julius B. T. Leach K. A. Tran T. M. Mertz R. A. Braun D. M. (2017). Sugar transporters in plants: new insights and discoveries. Plant Cell Physiol. 58, 1442–1460. doi: 10.1093/pcp/pcx090 28922744

[B79] Karalija E. Vergata C. Basso M. F. Negussu M. Zaccai M. Grossi-de-Sa M. F.. (2022). Chickpeas’ tolerance of drought and heat: Current knowledge and next steps. Agronomy 12, 2248. doi: 10.3390/agronomy12102248

[B80] Katyayini N. U. Rinne P. L. H. Tarkowská D. Strnad M. Van der Schoot C. (2020). Dual role of gibberellin in perennial shoot branching: Inhibition and activation. Front. Plant Sci. 11. doi: 10.3389/fpls.2020.00736 PMC7289990 32582259

[B81] Kebrom T. H. (2017). A growing stem inhibits bud outgrowth - The overlooked theory of apical dominance. Front. Plant Sci. 8. doi: 10.3389/fpls.2017.01874 PMC5671643 29163599

[B82] Kelly G. David-Schwartz R. Sade N. Moshelion M. Levi A. Alchanatis V.. (2012). The pitfalls of transgenic selection and new roles of AtHXK1: a high level of AtHXK1 expression uncouples hexokinase1-dependent sugar signaling from exogenous sugar. Plant Physiol. 159, 47–51. doi: 10.1104/pp.112.196105 22451715 PMC3375979

[B83] Kieber J. J. Schaller G. E. (2018). Cytokinin signaling in plant development. Development 145, dev149344. doi: 10.1242/dev.149344 29487105

[B84] Kim A. Chen J. Khare D. Jin J. Y. Yamaoka Y. Maeshima M.. (2020). Non-intrinsic ATP-binding cassette proteins ABCI19, ABCI20 and ABCI21 modulate cytokinin response at the endoplasmic reticulum in *Arabidopsis thaliana* . Plant Cell Rep. 39, 473–487. doi: 10.1007/s00299-019-02503-0 32016506 PMC7346704

[B85] Kim D. Paggi J. M. Park C. Bennett C. Salzberg S. L. (2019). Graph-based genome alignment and genotyping with HISAT2 and HISAT-genotype. Nat. Biotechnol. 37, 907–915. doi: 10.1038/s41587-019-0201-4 31375807 PMC7605509

[B86] Korek M. Marzec M. (2023). Strigolactones and abscisic acid interactions affect plant development and response to abiotic stresses. BMC Plant Biol. 23, 314. doi: 10.1186/s12870-023-04332-6 37308831 PMC10262459

[B87] Koul B. Sharma K. Sehgal V. Yadav D. Mishra M. Bharadwaj C. (2022). Chickpea (*Cicer arietinum* L.) biology and biotechnology: From domestication to biofortification and biopharming. Plants 11, 2926. doi: 10.3390/plants11212926 36365379 PMC9654780

[B88] Kucukoglu M. Nilsson J. Zheng B. Chaabouni S. Nilsson O. (2017). WUSCHEL-RELATED HOMEOBOX4 (WOX4)-like genes regulate cambial cell division activity and secondary growth in Populus trees. New Phytol. 215, 642–657. doi: 10.1111/nph.14631 28609015

[B89] Kumar M. N. Verslues P. E. (2015). Stress physiology functions of the Arabidopsis histidine kinase cytokinin receptors. Physiologia Plantarum 154, 369–380. doi: 10.1111/ppl.12290 25263537

[B90] Kuroha T. Tokunaga H. Kojima M. Ueda N. Ishida T. Nagawa S.. (2009). Functional analyses of LONELY GUY cytokinin-activating enzymes reveal the importance of the direct activation pathway in Arabidopsis. Plant Cell 21, 3152–3169. doi: 10.1105/tpc.109.068676 19837870 PMC2782294

[B91] Landi N. Piccolella S. Ragucci S. Faramarzi S. Clemente A. Papa S.. (2021). Valle Agricola Chickpeas: Nutritional profile and metabolomics traits of a typical landrace legume from Southern Italy. Foods 10, 3390. doi: 10.3390/foods10030583 PMC8002183 33802023

[B92] Lee S. H. Cho H. T. (2006). PINOID positively regulates auxin efflux in Arabidopsis root hair cells and tobacco cells. Plant Cell 18, 1604–1616. doi: 10.1105/tpc.105.035972 16731587 PMC1488908

[B93] Lee D. K. Geisler M. Springer P. S. (2009). LATERAL ORGAN FUSION1 and LATERAL ORGAN FUSION2 function in lateral organ separation and axillary meristem formation in Arabidopsis. Development 136, 2423–2432. doi: 10.1242/dev.031971 19542355

[B94] Lemoine R. La Camera S. Atanassova R. Dédaldéchamp F. Allario T. Pourtau N.. (2013). Source-to-sink transport of sugar and regulation by environmental factors. Front. Plant Sci. 4. doi: 10.3389/fpls.2013.00272 PMC3721551 23898339

[B95] Li N. Bo C. Zhang Y. Wang L. (2021). PHYTOCHROME INTERACTING FACTORS PIF4 and PIF5 promote heat stress induced leaf senescence in Arabidopsis. J. Exp. Bot. 72, 4577–4589. doi: 10.1093/jxb/erab158 33830198 PMC8446286

[B96] Li J. Liu Y. Zhang J. Cao L. Xie Q. Chen G.. (2023). Suppression of a hexokinase gene *SlHXK1* in tomato affects fruit setting and seed quality. Plant Physiol. Biochem. 205, 108160. doi: 10.1016/j.plaphy.2023.108160 37944243

[B97] Li Z.-Y. Ma N. Zhang F.-J. Li L.-Z. Li H.-J. Wang X.-F.. (2024). Functions of phytochrome interacting factors (PIFs) in adapting plants to biotic and abiotic stresses. Int. J. Mol. Sci. 25, 2198. doi: 10.3390/ijms25042198 38396875 PMC10888771

[B98] Li A. Sun X. Liu L. (2022). Action of salicylic acid on plant growth. Front. Plant Sci. 13. doi: 10.3389/fpls.2022.878076 PMC9093677 35574112

[B99] Lian Y. Lian C. Wang L. Li Z. Yuan G. Xuan L.. (2023). Suppressor Of Max2 Like 6, 7, And 8 Interact With Ddb1 Binding Wd Repeat Domain Hypersensitive To Aba Deficient 1 To Regulate The Drought Tolerance And Target Sucrose Nonfermenting 1 Related Protein Kinase 2.3 to abscisic acid response in Arabidopsis. Biomolecules 13, 1406. doi: 10.3390/biom13091406 37759806 PMC10526831

[B100] Liang Y. Ward S. Li P. Bennett T. Leyser O. (2016). SMAX1-LIKE7 signals from the nucleus to regulate shoot development in Arabidopsis via partially EAR motif-independent mechanisms. Plant Cell 28, 1581–1601. doi: 10.1105/tpc.16.00286 27317673 PMC4981136

[B101] Liao Y. Smyth G. K. Shi W. (2013). featureCounts: an efficient general purpose program for assigning sequence reads to genomic features. Bioinformatics 30, 923–930. doi: 10.1093/bioinformatics/btt656 24227677

[B102] Liber M. Duarte I. Maia A. T. Oliveira H. R. (2021). The history of lentil (*Lens culinaris* subsp. Culinaris) domestication and spread as revealed by genotyping-by-sequencing of wild and landrace accessions. Front. Plant Sci. 12. doi: 10.3389/fpls.2021.628439 PMC8030269 33841458

[B103] Lin Q. Wang J. Gong J. Zhang Z. Wang S. Sun J.. (2023). The *Arabidopsis thaliana* trehalose-6-phosphate phosphatase gene AtTPPI improve chilling tolerance through accumulating soluble sugar and JA. Environ. Exp. Bot. 205, 105117. doi: 10.1016/j.envexpbot.2022.105117

[B104] Lin Q. Yang J. Wang Q. Zhu H. Chen Z. Dao Y.. (2019). Overexpression of the trehalose-6-phosphate phosphatase family gene AtTPPF improves the drought tolerance of *Arabidopsis thaliana* . BMC Plant Biol. 19, 381. doi: 10.1186/s12870-019-1986-5 31477017 PMC6721209

[B105] Liu W. Peng B. Song A. Jiang J. Chen F. (2019). Sugar transporter, *CmSWEET17*, promotes bud outgrowth in *Chrysanthemum Morifolium* . Genes 11, 26. doi: 10.3390/genes11010026 31878242 PMC7017157

[B106] Lodha M. Marco C. F. Timmermans M. C. (2013). The ASYMMETRIC LEAVES complex maintains repression of KNOX homeobox genes via direct recruitment of Polycomb-repressive complex2. Genes Dev. 27, 596–601. doi: 10.1101/gad.211425.112 23468429 PMC3613607

[B107] Mandel M. A. Yanofsky M. F. (1995). The Arabidopsis AGL8 MADS box gene is expressed in inflorescence meristems and is negatively regulated by APETALA1. Plant Cell 7, 1763–1771. doi: 10.1105/tpc.7.11.1763 8535133 PMC161036

[B108] Marquès-Bueno M. M. Armengot L. Noack L. C. Bareille J. Rodriguez L. Platre M. P.. (2021). Auxin-regulated reversible inhibition of TMK1 signaling by MAKR2 modulates the dynamics of root gravitropism. Curr. Biol. 31, 228–237.e10. doi: 10.1016/j.cub.2020.10.011 33157019 PMC7809621

[B109] Martin R. C. Mok D. W. S. Smets R. Harry A. Onckelen V. Mok C. M. (2001). Development of transgenic tobacco harboring a zeatin O-glucosyltransferase gene from *Phaseolus* . In Vitro Cell. Dev. Biol. - Plant 37, 354–360. doi: 10.1007/s11627-001-0063-5

[B110] Martín-Fontecha E. S. Tarancón C. Cubas P. (2018). To grow or not to grow, a power-saving program induced in dormant buds. Curr. Opin. Plant Biol. 41, 102–109. doi: 10.1016/j.pbi.2017.10.001 29125947

[B111] Mason M. G. Ross J. J. Babst B. A. Wienclaw B. N. Beveridge C. A. (2014). Sugar demand, not auxin, is the initial regulator of apical dominance. PNAS 111, 6092–6097. doi: 10.1073/pnas.1322045111 24711430 PMC4000805

[B112] Maurya J. P. Miskolczi P. C. Mishra S. Singh R. K. Bhalerao R. P. (2020). A genetic framework for regulation and seasonal adaptation of shoot architecture in hybrid aspen. PNAS 117, 11523–11530. doi: 10.1073/pnas.2004705117 32393640 PMC7260942

[B113] Melzer S. Lens F. Gennen J. Vanneste S. Rohde A. Beeckman T. (2008). Flowering-time genes modulate meristem determinacy and growth form in *Arabidopsis thaliana* . Nat. Genet. 40, 1489–1492. doi: 10.1038/ng.253 18997783

[B114] Mengarelli D. A. Roldán Tewes L. Balazadeh S. Zanor M. I. (2021). FITNESS acts as a negative regulator of immunity and influences the plant reproductive output after *Pseudomonas syringae* infection. Front. Plant Sci. 12. doi: 10.3389/fpls.2021.606791 PMC7889524 33613599

[B115] Min Z. Chen L. Zhang Y. Li Z. Liu M. Li W. P.. (2021). VvBRC inhibits shoot branching in grapevine. Scientia Hortic. 289, 110370. doi: 10.1016/j.scienta.2021.110370

[B116] Mitache M. Baidani A. Bencharki B. Idrissi O. (2024). Exploring the impact of light intensity under speed breeding conditions on the development and growth of lentil and chickpea. Plant Methods 20, 30. doi: 10.1186/s13007-024-01156-9 38369489 PMC10874544

[B117] Miyagawa Y. Tamoi M. Shigeoka S. (2001). Overexpression of a cyanobacterial fructose-1,6-/sedoheptulose-1, 7-bisphosphatase in tobacco enhances photosynthesis and growth. Nat. Biotechnol. 19, 965–969. doi: 10.1038/nbt1001-965 11581664

[B118] Morales-Herrera S. Jourquin J. Coppé F. Lopez-Galvis L. De Smet T. Safi A.. (2023). Trehalose-6-phosphate signaling regulates lateral root formation in *Arabidopsis thaliana* . PNAS 120, e2302996120. doi: 10.1073/pnas.2302996120 37748053 PMC10556606

[B119] Müller D. Leyser O. (2011). Auxin, cytokinin and the control of shoot branching. Ann. Bot. 107, 1203–1212. doi: 10.1093/aob/mcr069 21504914 PMC3091808

[B120] Munguía-Rodríguez A. G. López-Bucio J. S. Ruiz-Herrera L. F. Ortiz-Castro R. Guevara-García Á.A. Marsch-Martínez N.. (2020). *YUCCA4* overexpression modulates auxin biosynthesis and transport and influences plant growth and development via crosstalk with abscisic acid in *Arabidopsis thaliana* . Genet. Mol. Biol. 43, e20190221. doi: 10.1590/1678-4685-gmb-2019-0221 32105289 PMC7197984

[B121] Nakata M. Matsumoto N. Tsugeki R. Rikirsch E. Laux T. Okada K. (2012). Roles of the middle domain-specific WUSCHEL-RELATED HOMEOBOX genes in early development of leaves in Arabidopsis. Plant Cell 24, 519–535. doi: 10.1105/tpc.111.092858 22374393 PMC3315230

[B122] Naz A. A. Raman S. Martinez C. C. Sinha N. R. Schmitz G. Theres K. (2013). Trifoliate encodes an MYB transcription factor that modulates leaf and shoot architecture in tomato. PNAS 110, 2401–2406. doi: 10.1073/pnas.1214300110 23341595 PMC3568312

[B123] Neff M. M. Nguyen S. M. Malancharuvil E. J. Fujioka S. Noguchi T. Seto H.. (1999). BAS1: A gene regulating brassinosteroid levels and light responsiveness in Arabidopsis. PNAS 96, 15316–15323. doi: 10.1073/pnas.96.26.15316 10611382 PMC24817

[B124] Nunes C. O’Hara L. E. Primavesi L. F. Delatte T. L. Schluepmann H. Somsen G. W.. (2013). The trehalose 6-phosphate/SnRK1 signaling pathway primes growth recovery following relief of sink limitation. Plant Physiol. 162, 1720–1732. doi: 10.1104/pp.113.220657 23735508 PMC3707538

[B125] O’Malley R. C. Huang S. C. Song L. Lewsey M. G. Bartlett A. Nery J. R.. (2016). Cistrome and epicistrome features shape the regulatory DNA landscape. Cell 165, 1280–1292. doi: 10.1016/j.cell.2016.04.038 27203113 PMC4907330

[B126] Ongaro V. Bainbridge K. Williamson L. Leyser O. (2008). Interactions between axillary branches of Arabidopsis. Mol. Plant 1, 388–400. doi: 10.1093/mp/ssn007 19825548

[B127] Ongaro V. Leyser O. (2007). Hormonal control of shoot branching. J. Exp. Bot. 59, 67–74. doi: 10.1093/jxb/erm134 17728300

[B128] Osakabe Y. Miyata S. Urao T. Seki M. Shinozaki K. Yamaguchi-Shinozaki K. (2002). Overexpression of Arabidopsis response regulators, ARR4/ATRR1/IBC7 and ARR8/ATRR3, alters cytokinin responses differentially in the shoot and in callus formation. Biochem. Biophys. Res. Commun. 293, 806–815. doi: 10.1016/S0006-291X(02)00286-3 12054542

[B129] Osella A. V. Mengarelli D. A. Mateos J. Dong S. Yanovsky M. J. Balazadeh S.. (2018). FITNESS, a CCT domain-containing protein, deregulates reactive oxygen species levels and leads to fine-tuning trade-offs between reproductive success and defence responses in Arabidopsis. Plant Cell Environ. 41, 2328–2341. doi: 10.1111/pce.13354 29852518

[B130] Otori K. Tamoi M. Tanabe N. Shigeoka S. (2017). Enhancements in sucrose biosynthesis capacity affect shoot branching in Arabidopsis. Biosci. Biotechnol. Biochem. 81, 1470–1477. doi: 10.1080/09168451.2017.1321954 28471323

[B131] Otori K. Tanabe N. Tamoi M. Shigeoka S. (2019). Sugar Transporter Protein 1 (STP1) contributes to regulation of the genes involved in shoot branching via carbon partitioning in Arabidopsis. Biosci. Biotechnol. Biochem. 83, 472–481. doi: 10.1080/09168451.2018.1550355 30488772

[B132] Overvoorde P. J. Okushima Y. Alonso J. M. Chan A. Chang C. Ecker J. R.. (2005). Functional genomic analysis of the AUXIN/INDOLE-3-ACETIC ACID gene family members in *Arabidopsis thaliana* . . Plant Cell 17, 3282–3300. doi: 10.1105/tpc.105.036723 16284307 PMC1315369

[B133] Paul M. J. Gonzalez-Uriarte A. Griffiths C. A. Hassani-Pak K. (2018). The role of trehalose 6-phosphate in crop yield and resilience. Plant Physiol. 177, 12–23. doi: 10.1104/pp.17.01634 29592862 PMC5933140

[B134] Paull R. E. Ksouri N. Kantar M. Zerpa-Catanho D. Chen N. J. Uruu G.. (2023). Differential gene expression during floral transition in pineapple. Plant Direct 7, e541. doi: 10.1002/pld3.541 38028646 PMC10644199

[B135] Peters S. Egert A. Stieger B. Keller F. (2010). Functional identification of Arabidopsis *ATSIP2* (At3g57520) as an alkaline α-galactosidase with a substrate specificity for raffinose and an apparent sink-specific expression pattern. Plant Cell Physiol. 51, 1815–1819. doi: 10.1093/pcp/pcq127 20739305

[B136] Piergiovanni A. R. (2022). *Ex situ* conservation of plant genetic resources: An overview of chickpea (*Cicer arietinum* L.) and lentil (*Lens culinaris* Medik.) worldwide collections. Diversity 14, 941. doi: 10.3390/d14110941

[B137] Ponnu J. Wahl V. Schmid M. (2011). Trehalose-6-phosphate: Connecting plant metabolism and development. Front. Plant Sci. 2. doi: 10.3389/fpls.2011.00070 PMC3355582 22639606

[B138] Qiu Y. Guan S. C. Wen C. Li P. Gao Z. Chen X. (2019). Auxin and cytokinin coordinate the dormancy and outgrowth of axillary bud in strawberry runner. BMC Plant Biol. 19, 528. doi: 10.1186/s12870-019-2151-x 31783789 PMC6884756

[B139] Rampey R. A. Woodward A. W. Hobbs B. N. Tierney M. P. Lahner B. Salt D. E.. (2006). An Arabidopsis basic helix-loop-helix leucine zipper protein modulates metal homeostasis and auxin conjugate responsiveness. Genetics 174, 1841–1857. doi: 10.1534/genetics.106.061044 17028341 PMC1698629

[B140] Ramsay L. Koh C. S. Kagale S. Gao D. Kaur S. Haile T.. (2021). Genomic rearrangements have consequences for introgression breeding as revealed by genome assemblies of wild and cultivated lentil species. BioRxiv. doi: 10.1101/2021.07.23.453237

[B141] Reddy D. S. Bhatnagar-Mathur P. Reddy P. S. Sri Cindhuri K. Sivaji Ganesh A. Sharma K. K. (2016). Identification and validation of reference genes and their impact on normalized gene expression studies across cultivated and wild *Cicer* species. PloS One 11, e0148451. doi: 10.1371/journal.pone.0148451 26863232 PMC4749333

[B142] Ren C. Guo Y. Kong J. Lecourieux F. Dai Z. Li S.. (2020). Knockout of *VvCCD8* gene in grapevine affects shoot branching. BMC Plant Biol. 20, 47. doi: 10.1186/s12870-020-2263-3 31996144 PMC6990564

[B143] Ren B. Liang Y. Deng Y. Chen Q. Zhang J. Yang X.. (2009). Genome-wide comparative analysis of type-A Arabidopsis response regulator genes by overexpression studies reveals their diverse roles and regulatory mechanisms in cytokinin signaling. Cell Res. 19, 1178–1190. doi: 10.1038/cr.2009.88 19621034

[B144] Robinson M. D. McCarthy D. J. Smyth G. K. (2009). edgeR: a Bioconductor package for differential expression analysis of digital gene expression data. Bioinformatics 26, 139–140. doi: 10.1093/bioinformatics/btp616 19910308 PMC2796818

[B145] Rodo A. P. Brugière N. Vankova R. Malbeck J. Olson J. M. Haines S. C.. (2008). Over-expression of a zeatin O-glucosylation gene in maize leads to growth retardation and tasselseed formation. J. Exp. Bot. 59, 2673–2686. doi: 10.1093/jxb/ern137 18515825 PMC2486472

[B146] Sakuraba Y. Jeong J. Kang M.-Y. Kim J. Paek N.-C. Choi G. (2014). Phytochrome-interacting transcription factors PIF4 and PIF5 induce leaf senescence in Arabidopsis. Nat. Commun. 5, 4636. doi: 10.1038/ncomms5636 25119965

[B147] Salam B. B. Barbier F. Danieli R. Teper-Bamnolker P. Ziv C. Spíchal L.. (2021). Sucrose promotes stem branching through cytokinin. Plant Physiol. 185, 1708–1721. doi: 10.1093/plphys/kiab003 33793932 PMC8133652

[B148] Salam B. B. Malka S. K. Zhu X. Gong H. Ziv C. Teper-Bamnolker P.. (2017). Etiolated stem branching is a result of systemic signaling associated with sucrose level. Plant Physiol. 175, 734–745. doi: 10.1104/pp.17.00995 28860154 PMC5619910

[B149] Sánchez-Gerschon V. Ferrándiz C. Balanzà V. (2023). HB21/40/53 promote inflorescence arrest through ABA accumulation at the end of flowering. bioRxiv. doi: 10.1101/2023.04.20.537726 PMC11288733 38669447

[B150] Sandhu J. S. Singh S. (2007). “History and origin,” in Lentil: An ancient crop for modern times. Eds. Yadav S. S. McNeil D. L. Stevenson P. C. (Springer Netherlands, Dordrecht), 1–9. 461 p. doi: 10.1007/978-1-4020-6313-8

[B151] Sato A. Yamamoto K. T. (2008). Overexpression of the non-canonical *Aux/IAA* genes causes auxin-related aberrant phenotypes in Arabidopsis. Physiologia Plantarum 133, 397–405. doi: 10.1111/j.1399-3054.2008.01055.x 18298415

[B152] Schneider S. Schneidereit A. Konrad K. R. Hajirezaei M. R. Gramann M. Hedrich R.. (2006). Arabidopsis INOSITOL TRANSPORTER4 mediates high-affinity H+ symport of myo-inositol across the plasma membrane. Plant Physiol. 141, 565–577. doi: 10.1104/pp.106.077123 16603666 PMC1475457

[B153] Schröder F. Lisso J. Müssig C. (2012). Expression pattern and putative function of *EXL1* and homologous genes in Arabidopsis. Plant Signaling Behav. 7, 22–27. doi: 10.4161/psb.7.1.18369 PMC3357360 22301961

[B154] Seale M. Bennett T. Leyser O. (2017). BRC1 expression regulates bud activation potential but is not necessary or sufficient for bud growth inhibition in Arabidopsis. Development 144, 1661–1673. doi: 10.1242/dev.145649 28289131 PMC5450845

[B155] Semiarti E. Ueno Y. Tsukaya H. Iwakawa H. Machida C. Machida Y. (2001). The *ASYMMETRIC LEAVES2* gene of *Arabidopsis thaliana* regulates formation of a symmetric lamina, establishment of venation and repression of meristem-related homeobox genes in leaves. Development 128, 1771–1783. doi: 10.1242/dev.128.10.1771 11311158

[B156] Shimizu-Sato S. Tanaka M. Mori H. (2009). Auxin-cytokinin interactions in the control of shoot branching. Plant Mol. Biol. 69, 429–435. doi: 10.1007/s11103-008-9416-3 18974937

[B157] Silva-Perez V. Shunmugam A. S. K. Rao S. Cossani C. M. Tefera A. T. Fitzgerald G. J.. (2022). Breeding has selected for architectural and photosynthetic traits in lentils. Front. Plant Sci. 13. doi: 10.3389/fpls.2022.925987 PMC9453451 36092438

[B158] Singh U. Gaur P. M. Chaturvedi S. K. Hazra K. K. Singh G. (2019). Changing plant architecture and density can increase chickpea productivity and facilitate for mechanical harvesting. Int. J. Plant Production 13, 193–202. doi: 10.1007/s42106-019-00047-7

[B159] Sinha R. Sharma T. R. Singh A. K. (2019). Validation of reference genes for qRT-PCR data normalisation in lentil (*Lens culinaris*) under leaf developmental stages and abiotic stresses. Physiol. Mol. Biol. Plants 25, 123–134. doi: 10.1007/s12298-018-0609-1 30804635 PMC6352542

[B160] Snowden K. C. Simkin A. J. Janssen B. J. Templeton K. R. Loucas H. M. Simons J. L.. (2005). The decreased apical dominance1/*Petunia hybrida* CAROTENOID CLEAVAGE DIOXYGENASE 8 gene affects branch production and plays a role in leaf senescence, root growth, and flower development. Plant Cell 17, 746–759. doi: 10.1105/tpc.104.027714 15705953 PMC1069696

[B161] Song Y. Xu Z.-F. (2013). Ectopic overexpression of an AUXIN/INDOLE-3-ACETIC ACID (Aux/IAA) gene *OsIAA4* in rice induces morphological changes and reduces responsiveness to auxin. Int. J. Mol. Sci. 14, 13645–13656. doi: 10.3390/ijms140713645 23812082 PMC3742208

[B162] Stefan T. Wu X. N. Zhang Y. Fernie A. Schulze W. X. (2022). Regulatory modules of metabolites and protein phosphorylation in Arabidopsis genotypes with altered sucrose allocation. Front. Plant Sci. 13. doi: 10.3389/fpls.2022.891405 PMC9161306 35665154

[B163] Stein O. Granot D. (2019). An overview of sucrose synthases in plants. Front. Plant Sci. 10. doi: 10.3389/fpls.2019.00095 PMC6375876 30800137

[B164] Stirnberg P. Zhao S. Williamson L. Ward S. Leyser O. (2012). FHY3 promotes shoot branching and stress tolerance in Arabidopsis in an AXR1-dependent manner. Plant J. 71, 907–920. doi: 10.1111/j.1365-313X.2012.05038.x 22540368

[B165] Strobl S. M. Kischka D. Heilmann I. Mouille G. Schneider S. (2018). The tonoplastic inositol transporter INT1 from *Arabidopsis thaliana* impacts cell elongation in a sucrose-dependent way. Front. Plant Sci. 9. doi: 10.3389/fpls.2018.01657 PMC6250803 30505313

[B166] Sun L. Feraru E. Feraru M. I. Waidmann S. Wang W. Passaia G.. (2020). PIN-LIKES coordinate brassinosteroid signaling with nuclear auxin input in *Arabidopsis thaliana* . Curr. Biol. 30, 1579–1588.e6. doi: 10.1016/j.cub.2020.02.002 32169207 PMC7198975

[B167] Sun H. Li W. Burritt D. J. Tian H. Zhang H. Liang X.. (2022). Strigolactones interact with other phytohormones to modulate plant root growth and development. Crop J. 10, 1517–1527. doi: 10.1016/j.cj.2022.07.014

[B168] Sun Y. Zhou Q. Zhang W. Fu Y. Huang H. (2002). ASYMMETRIC LEAVES1, an Arabidopsis gene that is involved in the control of cell differentiation in leaves. Planta 214, 694–702. doi: 10.1007/s004250100673 11882937

[B169] Takei K. Yamaya T. Sakakibara H. (2004). Arabidopsis CYP735A1 and CYP735A2 encode cytokinin hydroxylases that catalyze the biosynthesis of trans-Zeatin. J. Biol. Chem. 279, 41866–41872. doi: 10.1074/jbc.M406337200 15280363

[B170] Tamoi M. Hiramatsu Y. Nedachi S. Otori K. Tanabe N. Maruta T.. (2014). Increase in the activity of fructose-1,6-bisphosphatase in cytosol affects sugar partitioning and increases the lateral shoots in tobacco plants at elevated CO_2_ levels. Photosynthesis Res. 108, 15–23. doi: 10.1007/s11120-011-9645-1 21400200

[B171] Tarancón C. González-Grandío E. Oliveros J. C. Nicolas M. Cubas P. (2017). A conserved carbon starvation response underlies bud dormancy in woody and herbaceous species. Front. Plant Sci. 8. doi: 10.3389/fpls.2017.00788 PMC5440562 28588590

[B172] Theodoris G. Inada N. Freeling M. (2003). Conservation and molecular dissection of ROUGH SHEATH2 and ASYMMETRIC LEAVES1 function in leaf development. PNAS 100, 6837–6842. doi: 10.1073/pnas.1132113100 12750468 PMC164533

[B173] Thimm O. Bläsing O. Gibon Y. Nagel A. Meyer S. Krüger P.. (2004). MAPMAN: a user-driven tool to display genomics data sets onto diagrams of metabolic pathways and other biological processes. Plant J. 37, 914–939. doi: 10.1111/j.1365-313X.2004.02016.x 14996223

[B174] Tripathi K. Kumari J. Gore P. G. Mishra D. C. Singh A. K. Mishra G. P.. (2022). Agro-morphological characterization of lentil germplasm of Indian national genebank and development of a core set for efficient utilization in lentil improvement programs. Front. Plant Sci. 12. doi: 10.3389/fpls.2021.751429 PMC8828943 35154171

[B175] Turk E. M. Fujioka S. Seto H. Shimada Y. Takatsuto S. Yoshida S.. (2005). BAS1 and SOB7 act redundantly to modulate Arabidopsis photomorphogenesis via unique brassinosteroid inactivation mechanisms. Plant J. 42, 23–34. doi: 10.1111/j.1365-313X.2005.02358.x 15773851

[B176] Usadel B. Nagel A. Steinhauser D. Gibon Y. Bläsing O. E. Redestig H.. (2006). PageMan: An interactive ontology tool to generate, display, and annotate overview graphs for profiling experiments. BMC Bioinf. 7, 535. doi: 10.1186/1471-2105-7-535 PMC1766370 17176458

[B177] van Es S. W. Muñoz-Gasca A. Romero-Campero F. J. González-Grandío E. de Los Reyes P. Tarancón C.. (2024). A gene regulatory network critical for axillary bud dormancy directly controlled by Arabidopsis BRANCHED1. New Phytol. 241, 1193–1209. doi: 10.1111/nph.19420 38009929

[B178] Van Houtte H. Vandesteene L. López-Galvis L. Lemmens L. Kissel E. Carpentier S.. (2013). Overexpression of the trehalase gene AtTRE1 leads to increased drought stress tolerance in Arabidopsis and is involved in abscisic acid-induced stomatal closure. Plant Physiol. 161, 1158–1171. doi: 10.1104/pp.112.211391 23341362 PMC3585587

[B179] Varshney R. K. Song C. Saxena R. K. Azam S. Yu S. Sharpe A. G.. (2013). Draft genome sequence of chickpea (*Cicer arietinum*) provides a resource for trait improvement. Nat. Biotechnol. 31, 240–246. doi: 10.1038/nbt.2491 23354103

[B180] Wallner E. S. López-Salmerón V. Belevich I. Poschet G. Jung I. Grünwald K.. (2017). Strigolactone- and karrikin-independent SMXL proteins are central regulators of phloem formation. Curr. Biol. 27, 1241–1247. doi: 10.1016/j.cub.2017.03.014 28392107 PMC5405109

[B181] Wallner E. S. Tonn N. Shi D. Luzzietti L. Wanke F. Hunziker P.. (2023). OBERON3 and SUPPRESSOR OF MAX2 1-LIKE proteins form a regulatory module driving phloem development. Nat. Commun. 14, 2128. doi: 10.1038/s41467-023-37790-5 37059727 PMC10104830

[B182] Wang M. Le Moigne M. A. Bertheloot J. Crespel L. Perez-Garcia M. D. Ogé L.. (2019). BRANCHED1: A key hub of shoot branching. Front. In Plant Sci. 10. doi: 10.3389/fpls.2019.00076 PMC6379311 30809235

[B183] Wang X. Liu D. Lin J. Zhu T. Liu N. Yang X.. (2021a). Carotenoid cleavage dioxygenase genes of *Chimonanthus praecox*, *CpCCD7* and *CpCCD8*, regulate shoot branching in Arabidopsis. Int. J. Mol. Sci. 22, 8750. doi: 10.3390/ijms22168750 34445457 PMC8395739

[B184] Wang X. Meng J. Deng L. Wang Y. Liu H. Yao J. L.. (2021b). Diverse functions of IAA-Leucine Resistant PpILR1 provide a genic basis for auxin-ethylene crosstalk during peach fruit ripening. Front. Plant Sci. 12. doi: 10.3389/fpls.2021.655758 PMC8149794 34054901

[B185] Wang L. Wang B. Jiang L. Liu X. Li X. Lu Z.. (2015). Strigolactone signaling in arabidopsis regulates shoot development by targeting D53-like SMXL repressor proteins for ubiquitination and degradation. Plant Cell 27, 3128–3142. doi: 10.1105/tpc.15.00605 26546446 PMC4682305

[B186] Wasternack C. (2015). How jasmonates earned their laurels: Past and present. J. Plant Growth Regul. 34, 761–794. doi: 10.1007/s00344-015-9526-5

[B187] Wei Z. Li J. (2020). Regulation of brassinosteroid homeostasis in higher plants. Front. Plant Sci. 11. doi: 10.3389/fpls.2020.583622 PMC7550685 33133120

[B188] Weijers D. Wagner D. (2016). Transcriptional responses to the auxin hormone. Annu. Rev. Plant Biol. 67, 539–574. doi: 10.1146/annurev-arplant-043015-112122 26905654

[B189] Weller J. L. Ortega R. (2015). Genetic control of flowering time in legumes. Front. Plant Sci. 6. doi: 10.3389/fpls.2015.00207 PMC4391241 25914700

[B190] Wickham H. (2016). ggplot2: Elegant graphics for data analysis (New York: Springer-Verlag), 2016. doi: 10.1007/978-3-319-24277-4

[B191] Wilcoxon F. (1945). Individual comparisons by ranking methods. Biometrics Bull. 1, 80–83. doi: 10.2307/3001968 18903631

[B192] Wingler A. (2017). Transitioning to the next phase: The role of sugar signaling throughout the plant life cycle. Plant Physiol. 176, 1075–1084. doi: 10.1104/pp.17.01229 28974627 PMC5813577

[B193] Wingler A. Henriques R. (2022). Sugars and the speed of life - Metabolic signals that determine plant growth, development and death. Physiologia Plantarum 174, e13656. doi: 10.1111/ppl.13656 35243645 PMC9314607

[B194] Winter C. M. Yamaguchi N. Wu M.-F. Wagner D. (2015). Transcriptional programs regulated by both LEAFY and APETALA1 at the time of flower formation. Physiol. Plantarum 155, 55–73. doi: 10.1111/ppl.12357 PMC5757833 26096587

[B195] Xia X. Dong H. Yin Y. Song X. Gu X. Sang K.. (2021). Brassinosteroid signaling integrates multiple pathways to release apical dominance in tomato. PNAS 118, e2004384118. doi: 10.1073/pnas.2004384118 33836559 PMC7980468

[B196] Xie Y. Liu Y. Ma M. Zhou Q. Zhao Y. Zhao B.. (2020). Arabidopsis FHY3 and FAR1 integrate light and strigolactone signaling to regulate branching. Nat. Commun. 11, 1955. doi: 10.1038/s41467-020-15893-7 32327664 PMC7181604

[B197] Xu E. Chai L. Zhang S. Yu R. Zhang X. Xu C.. (2021). Catabolism of strigolactones by a carboxylesterase. Nat. Plants 7, 1495–1504. doi: 10.1038/s41477-021-01011-y 34764442

[B198] Xu L. Xu Y. Dong A. Sun Y. Pi L. Xu Y.. (2003). Novel as1 and as2 defects in leaf adaxial-abaxial polarity reveal the requirement for ASYMMETRIC LEAVES1 and 2 and ERECTA functions in specifying leaf adaxial identity. Development 130, 4097–4107. doi: 10.1242/dev.00622 12874130

[B199] Yang T. Liu K. Poppy L. Mulenga A. Gampe C. (2021). Minimizing lentil harvest loss through improved agronomic practices in sustainable agro-systems. Sustainability 13, 1896. doi: 10.3390/su13041896

[B200] Yang Y. Nicolas M. Zhang J. Yu H. Guo D. Yuan R.. (2018). The TIE1 transcriptional repressor controls shoot branching by directly repressing BRANCHED1 in Arabidopsis. PloS Genet. 14, e1007296. doi: 10.1371/journal.pgen.1007296 29570704 PMC5884558

[B201] Yang Q. Yuan C. Cong T. Zhang Q. (2023). The secrets of meristems initiation: Axillary meristem initiation and floral meristem initiation. Plants 12, 1879. doi: 10.3390/plants12091879 37176937 PMC10181267

[B202] Yao C. Finlayson S. A. (2015). Abscisic acid is a general negative regulator of Arabidopsis axillary bud growth. Plant Physiol. 169, 611–626. doi: 10.1104/pp.15.00682 26149576 PMC4577412

[B203] Youn J.-H. Kim M. K. Kim E.-J. Son S.-H. Lee J. E. Jang M.-S.. (2016). ARF7 increases the endogenous contents of castasterone through suppression of BAS1 expression in *Arabidopsis thaliana* . Phytochemistry 122, 34–44. doi: 10.1016/j.phytochem.2015.11.006 26608667

[B204] Yu Y. Liu Z. Wang L. Kim S.-G. Seo P. J. Qiao M.. (2016). WRKY71 accelerates flowering via the direct activation of FLOWERING LOCUS T and LEAFY in *Arabidopsis thaliana* . Plant J. 85, 96–106. doi: 10.1111/tpj.13092 26643131

[B205] Yu Y. Wang L. Chen J. Liu Z. Park C.-M. Xiang F. (2017). WRKY71 acts antagonistically against salt-delayed flowering in *Arabidopsis thaliana* . Plant Cell Physiol. 59, 414–422. doi: 10.1093/pcp/pcx201 29272465

[B206] Yuan Y. Khourchi S. Li S. Du Y. Delaplace P. (2023). Unlocking the multifaceted mechanisms of bud outgrowth: Advances in Understanding Shoot Branching. Plants 12, 3628. doi: 10.3390/plants12203628 37896091 PMC10610460

[B207] Zhai H. Xiong L. Li H. Lyu X. Yang G. Zhao T.. (2020). Cryptochrome 1 inhibits shoot branching by repressing the self-activated transcription loop of PIF4 in Arabidopsis. Plant Commun. 1, 100042. doi: 10.1016/j.xplc.2020.100042 33367238 PMC7748022

[B208] Zhang J. Eswaran G. Alonso-Serra J. Kucukoglu M. Xiang J. Yang W.. (2019). Transcriptional regulatory framework for vascular cambium development in Arabidopsis roots. Nat. Plants 5, 1033–1042. doi: 10.1038/s41477-019-0522-9 31595065 PMC6795544

[B209] Zhang L. Fang W. Chen F. Song A. (2022). The role of transcription factors in the regulation of plant shoot branching. Plants 11, 1997. doi: 10.3390/plants11151997 35956475 PMC9370718

[B210] Zhang Y. Primavesi L. F. Jhurreea D. Andralojc P. J. Mitchell R. A. C. Powers S. J.. (2009). Inhibition of SNF1-related protein kinase1 activity and regulation of metabolic pathways by trehalose-6-phosphate. Plant Physiol. 149, 1860–1871. doi: 10.1104/pp.108.133934 19193861 PMC2663748

[B211] Zhao Y. (2010). Auxin biosynthesis and its role in plant development. Annu. Rev. Plant Biol. 61, 49–64. doi: 10.1146/annurev-arplant-042809-112308 20192736 PMC3070418

[B212] Zhao H. Liu L. Mo H. Qian L. Cao Y. Cui S.. (2013). The ATP-binding cassette transporter ABCB19 regulates postembryonic organ separation in Arabidopsis. PloS One 8, e60809. doi: 10.1371/journal.pone.0060809 23560110 PMC3613370

[B213] Zhao H. Maokai Y. Cheng H. Guo M. Liu Y. Wang L.. (2021). Characterization of auxin transporter AUX, PIN and PILS gene families in pineapple and evaluation of expression profiles during reproductive development and under abiotic stresses. PeerJ 9, e11410. doi: 10.7717/peerj.11410 34221708 PMC8231336

[B214] Zheng X. Yang X. Chen Z. Xie W. Yue X. Zhu H.. (2021). Arabidopsis SMAX1 overaccumulation suppresses rosette shoot branching and promotes leaf and petiole elongation. Biochem. Biophys. Res. Commun. 553, 44–50. doi: 10.1016/j.bbrc.2021.03.006 33756344

[B215] Zhou F. Lin Q. Zhu L. Ren Y. Zhou K. Shabek N.. (2013). D14-SCF(D3)-dependent degradation of D53 regulates strigolactone signalling. Nature 504, 406–410. doi: 10.1038/nature12878 24336215 PMC4096652

[B216] Zhou D. Wang X. Wang X. Mao T. (2023). PHYTOCHROME INTERACTING FACTOR 4 regulates microtubule organization to mediate high temperature-induced hypocotyl elongation in Arabidopsis. Plant Cell 35, 2044–2061. doi: 10.1093/plcell/koad042 36781395 PMC10226600

[B217] Zou H.-F. Zhang Y.-Q. Wei W. Chen H.-W. Song Q.-X. Liu Y.-F.. (2012). The transcription factor AtDOF4.2 regulates shoot branching and seed coat formation in Arabidopsis. Biochem. J. 449, 373–388. doi: 10.1042/BJ20110060 23095045

[B218] Zubo Y. O. Blakley I. C. Yamburenko M. V. Worthen J. M. Street I. H. Franco-Zorrilla J. M.. (2017). Cytokinin induces genome-wide binding of the type-B response regulator ARR10 to regulate growth and development in Arabidopsis. PNAS 114, E5995–E6004. doi: 10.1073/pnas.1620749114 28673986 PMC5530654

