# Supplementary material for: Genome-wide transcript expression analysis reveals major chickpea and lentil genes associated with plant branching
Source: Front Plant Sci. 2024 Jun 19;15:1384237. doi: 10.3389/fpls.2024.1384237 (PMC11220206; doi:10.3389/fpls.2024.1384237)
Supplement: Supplementary file 1 [file DataSheet_1.docx]

**SUPPORTING INFORMATION**


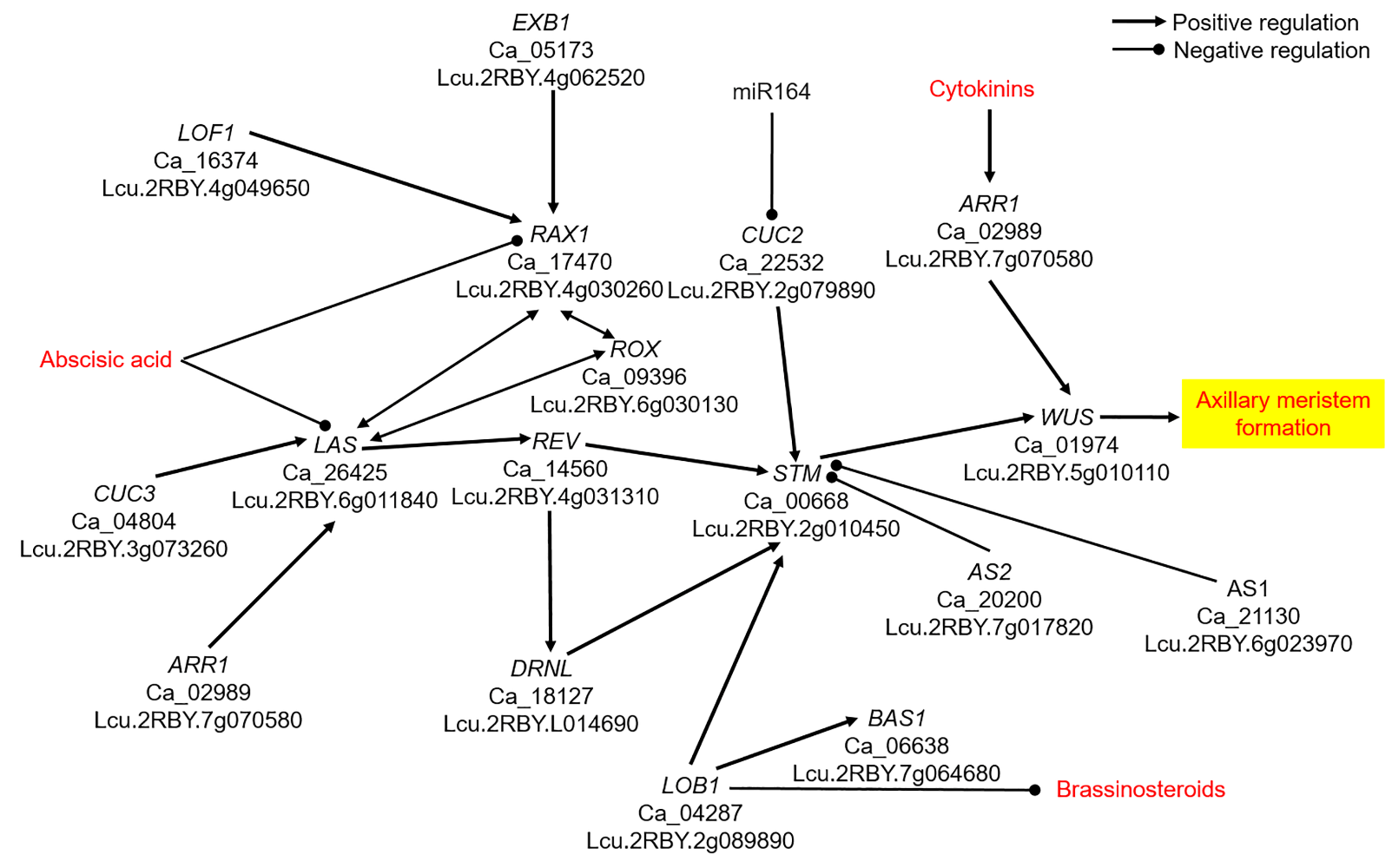


**Figure S1.** Major transcription factors involved in regulating axillary meristem formation in *Arabidopsis thaliana*, as reviewed by Zhang et al. (2022), and their orthologous genes (gene identifier) in chickpea and lentil.


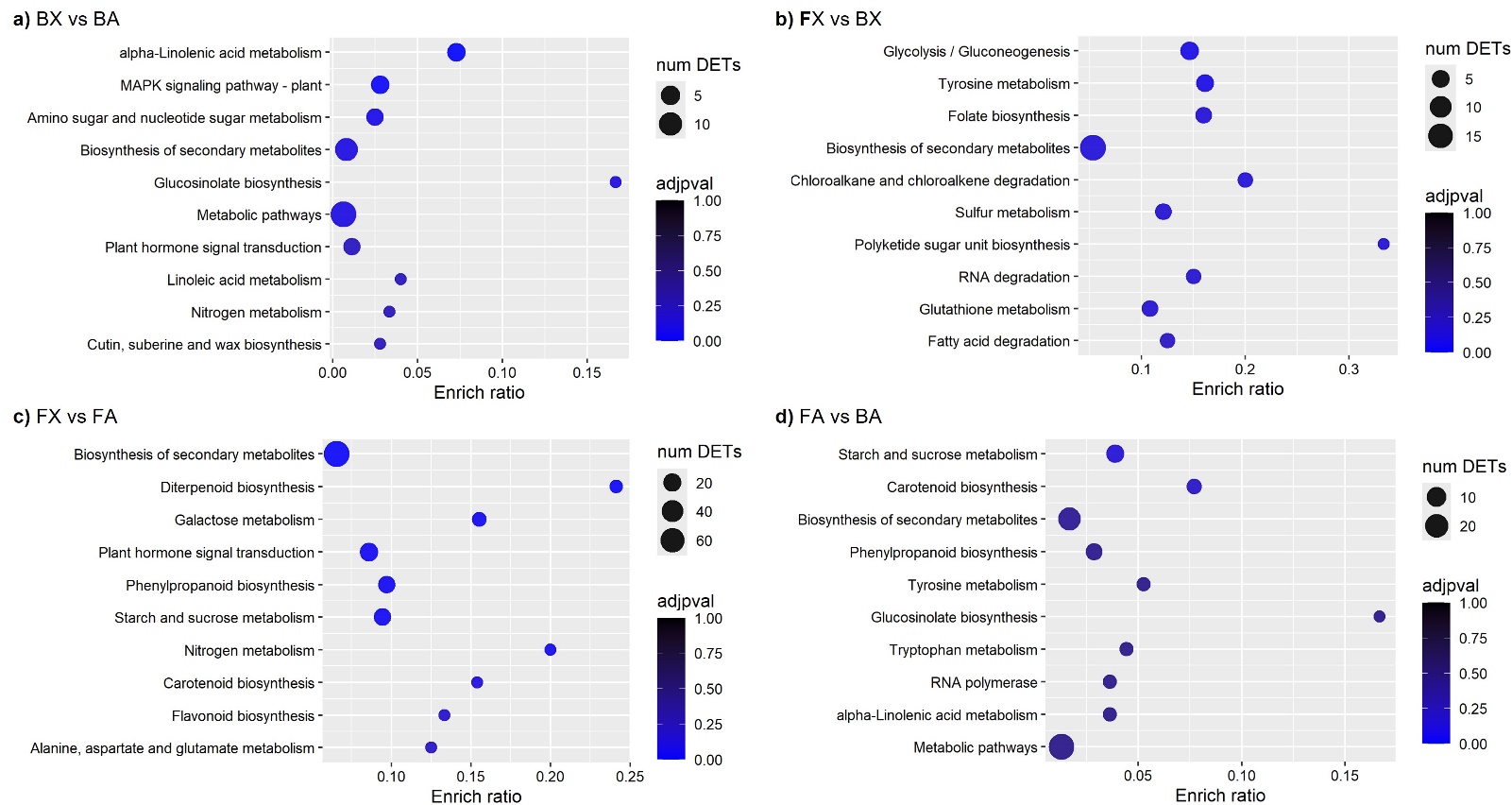


**Figure S2.** KEGG pathway enrichment analysis of differentially expressed transcript (DET) sets in pairwise comparisons between chickpea genotypes and tissues. (**a**) BX *versus* BA, (**b**) FX *versus* BX, (**c**) FX *versus* FA, (**d**) FA *versus* BA. KEGG enrichment bubble diagrams display the top 10 enriched KEGG categories. The X-axis represents the enrichment ratio based on number of DETs belonging to the KEGG category/number of DETs belonging to the same KEGG category in the background genome. The Y-axis represents the enriched KEGG categories ordered by the number of detected DETs. The size of the dots represents the number of DETs included in each KEGG category. The dots color represents the adjusted *p*-values for each KEGG category enrichment. BX: Blanco lechoso axillary bud, BA: Blanco lechoso apical bud, FX: FLIP07-318C axillary bud, FA: FLIP07-318C apical bud.


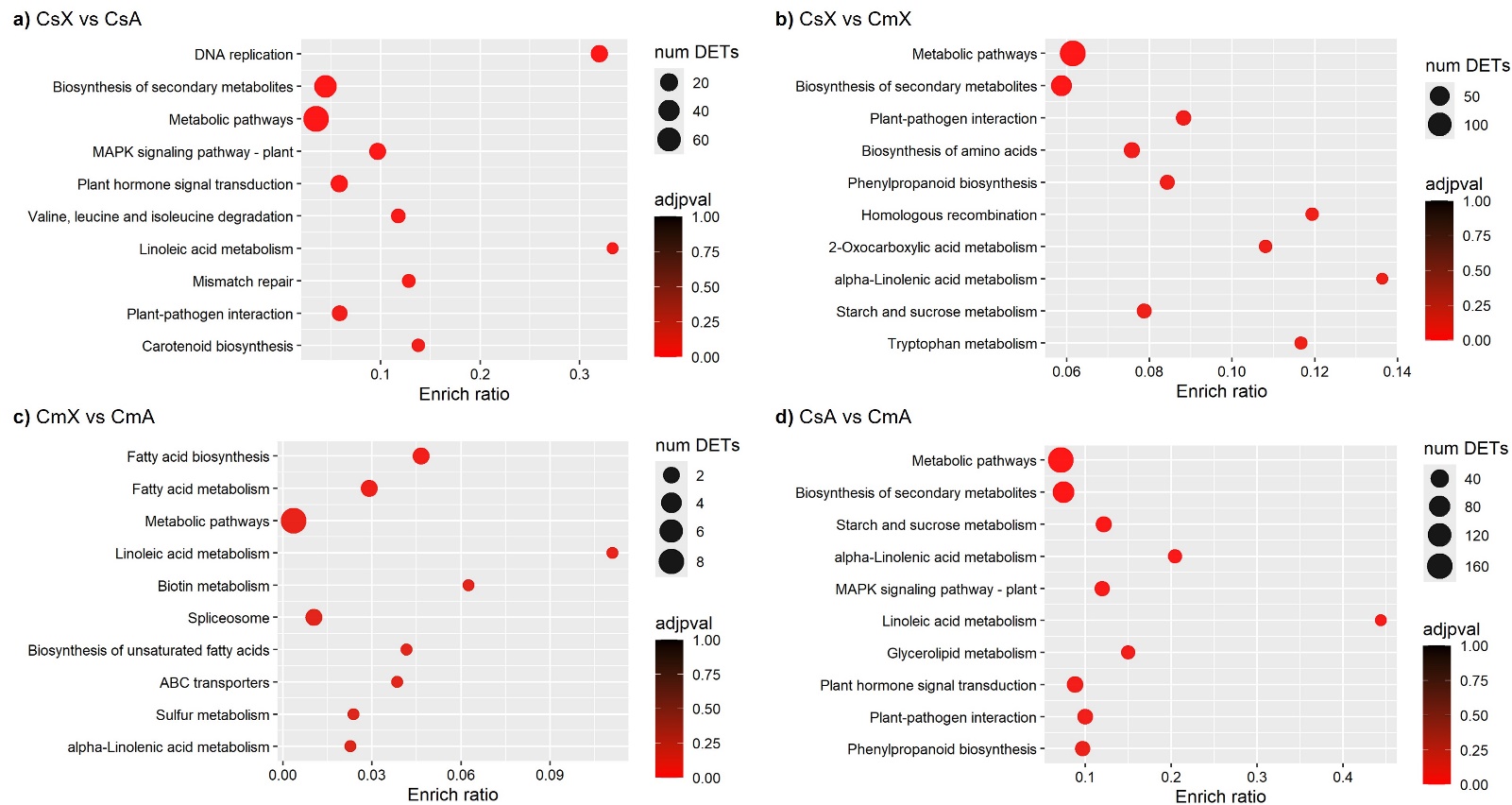


**Figure S3.** KEGG pathway enrichment analysis of differentially expressed transcript (DET) sets in pairwise comparisons between lentil genotypes and tissues. (**a**) CsX *versus* CsA, (**b**) CsX *versus* CmX, (**c**) CmX *versus* CmA, (**d**) CsA *versus* CmA. KEGG enrichment bubble diagrams display the top 10 enriched KEGG categories. The X-axis represents the enrichment ratio based on number of DETs belonging to the KEGG category/number of DETs belonging to the same KEGG category in the background genome. The Y-axis represents the enriched KEGG categories ordered by the number of detected DETs. The size of the dots represents the number of DETs included in each KEGG category. The dots color represents the adjusted *p*-values for each KEGG category enrichment. CsX: Castellana axillary bud, CsA: Castellana apical bud, CmX: Campisi axillary bud, and CmA: Campisi apical bud.


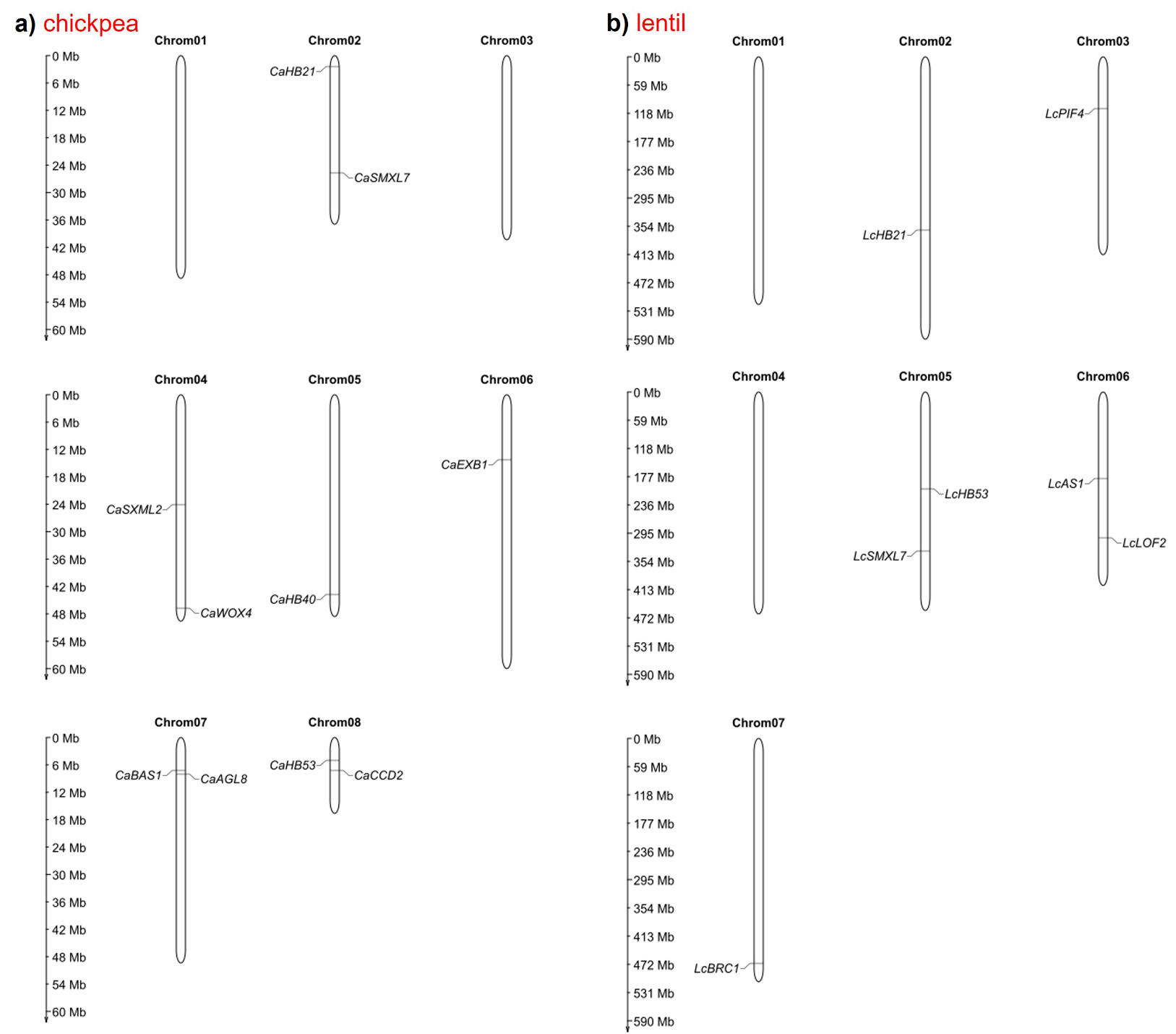


**Figure S4.** Chromosomal locations of the major genes associated with branching along the (**a**) chickpea and (**b**) lentil chromosomes. Relative chromosome length scaled in megabases (Mb) and bars on chromosomes indicate the gene positions.


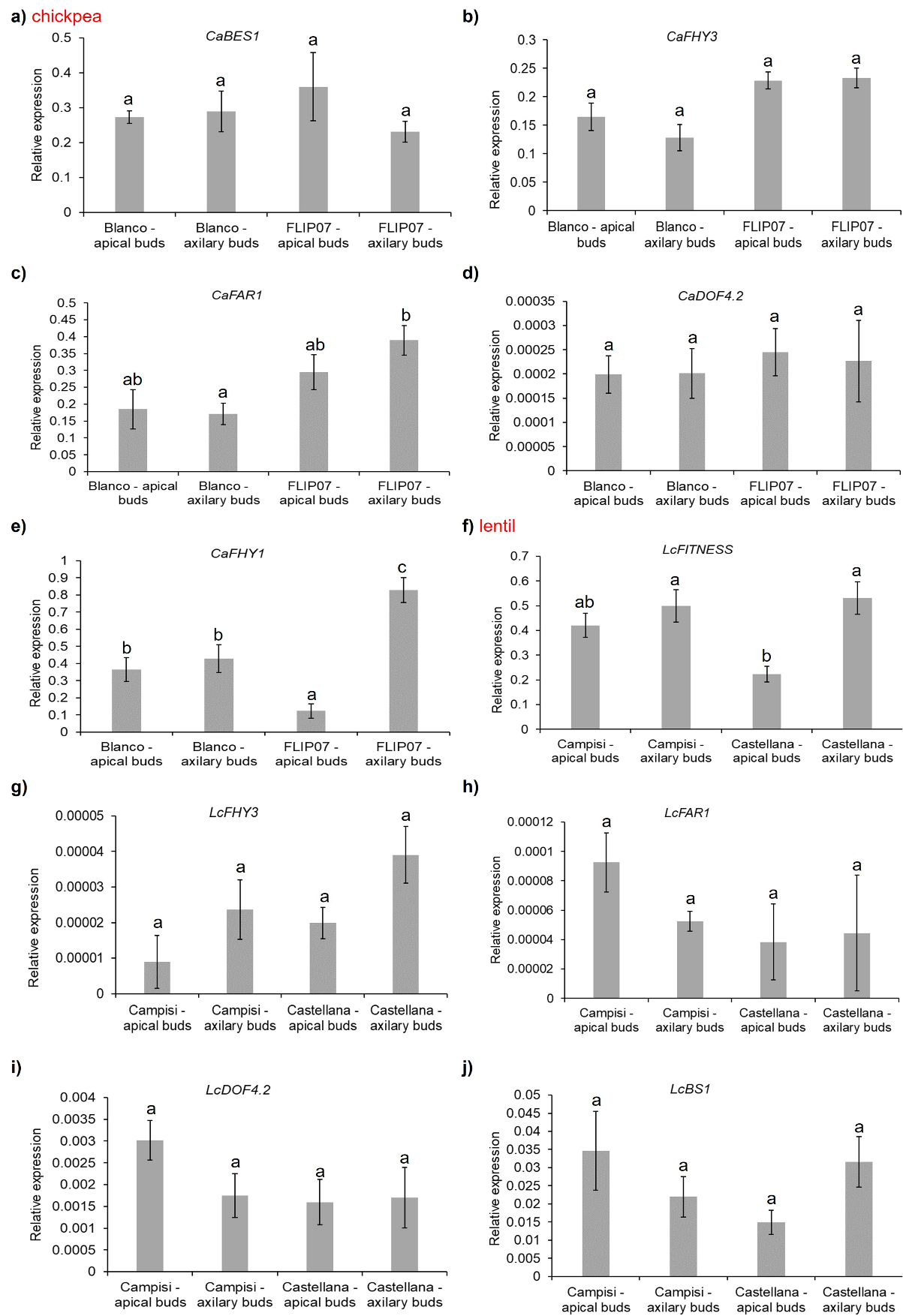


**Figure S5.** Gene expression profile measured by real-time RT-PCR in apical and axillary buds of chickpea cultivars Blanco lechoso and FLIP07-318C and lentil cultivars Campisi and Castellana. Relative expression profiles of chickpea **a)** *CaBES1*, **b)** *CaFHY3*, **c)** *CaFAR1*, **d)** *CaDOF4.2*, **e)** *CaFHY1*, and lentil **f)** *LcFITNESS*, **g)** *LcFHY3*, **h)** *LcFAR1*, **i)** *LcDOF4.2*, **j)** *LcBS1* genes. Gene expression values were calculated with the 2^-(∆Ct) formula and normalized with *CaCAC* and *LcTUB* as endogenous reference genes (Table S1). Error bars represent confidence intervals corresponding to three biological replicates. Different letters on the bars indicate significant statistical differences according to Tukey’s test at a 95% significance level.


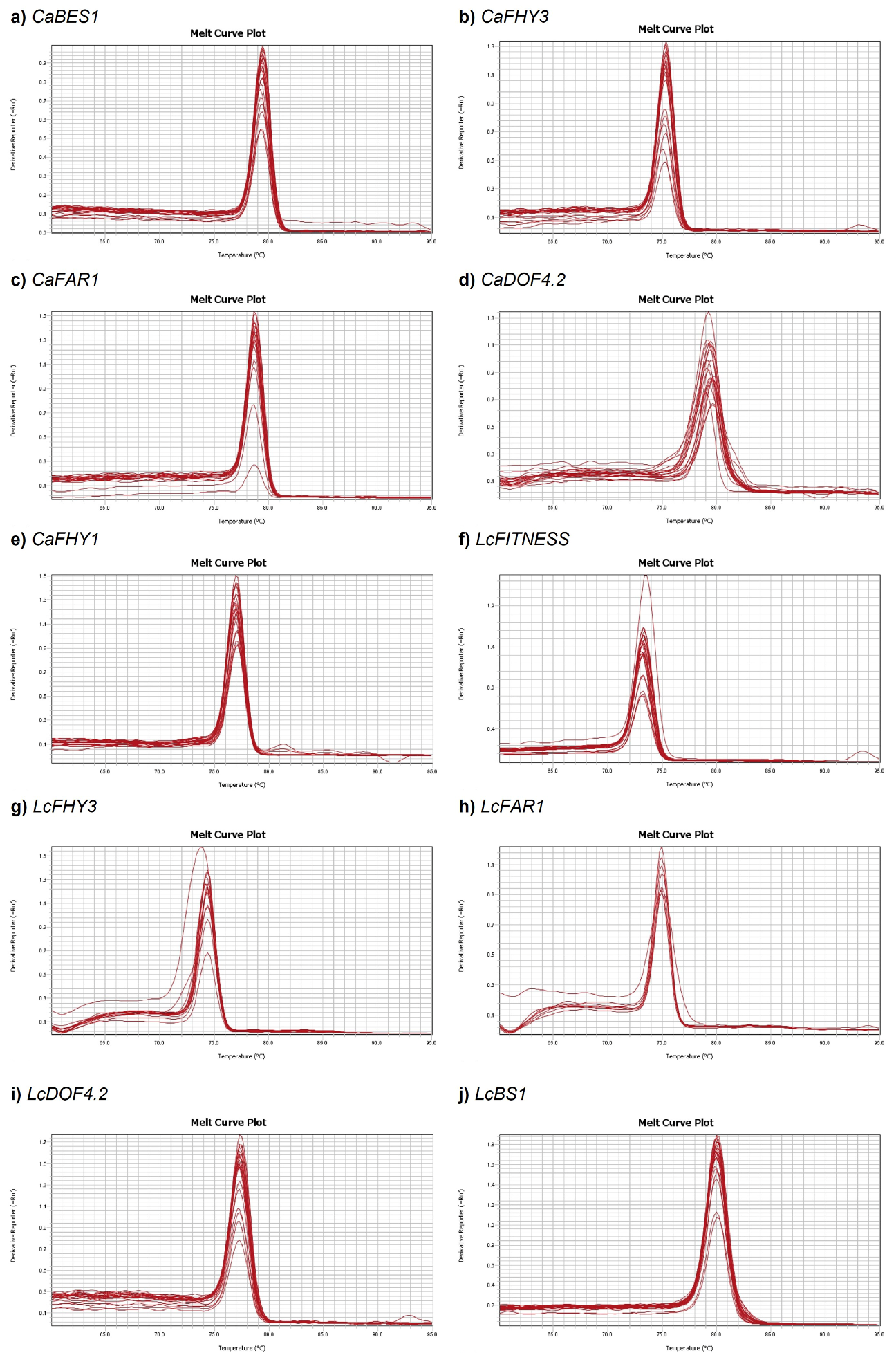


**Figure S6.** Melting curve plot of gene amplification by real-time RT-PCR in apical and axillary buds of chickpea cultivars Blanco lechoso and FLIP07-318C and lentil cultivars Campisi and Castellana. Plot of chickpea **a)** *CaBES1*, **b)** *CaFHY3*, **c)** *CaFAR1*, **d)** *CaDOF4.2*, **e)** *CaFHY1*, and lentil **f)** *LcFITNESS*, **g)** *LcFHY3*, **h)** *LcFAR1*, **i)** *LcDOF4.2*, **j)** *LcBS1* genes (Table S1).

**Table S1.** Primer sequences used in the real-time RT-PCR.

| **Gene** | **Primer name** | **Primer sequence** | ***Tm* (^o^C)** | **Amplicon (nt)** |
| --- | --- | --- | --- | --- |
| *CaBES1*  *Ca_04963* | CaBES1q(F) | TGCGAAGATATACGCGGGAT | 60 | 150 |
|  | CaBES1q(R) | ATTGCCCTTGATCCCTTGCG | 60 |  |
| *CaFHY3*  *Ca_02661* | Ca02661FHY3q(F) | TCCCGGAACACGTCATTGTT | 60 | 171 |
|  | Ca02661FHY3q(R) | CTGTCAACCAGCTTCCACCA | 60 |  |
| *CaFAR1*  *Ca_10247* | Ca10247FAR1q(F) | TGGGAATCACGTTTCCTCCG | 60 | 196 |
|  | Ca10247FAR1q(R) | AGGGTTTTCAGCCTGCACTT | 60 |  |
| *CaDOF4.2*  *Ca_00318* | Ca00318q(F) | TGGTGGTGGTTGTCGTAAGG | 60 | 112 |
|  | Ca00318q(R) | CATCGTCTGCTGTTGTGCAG | 60 |  |
| *CaFHY1*  *Ca_25363* | CaFHY1q(F) | CAGTTGGATTTGCTTAGACCGAA | 60 | 143 |
|  | CaFHY1q(R) | TCAGCGGATGAAGCATCGAC | 60 |  |
| *CaG6PD^*^*  *Ca_14672* | CaG6PDq(F) | ACAACGATACCAGGGTGTTACC | 60 | 116 |
|  | CaG6PDq(R) | TCTCCCATGATGCCTTTAACTC | 60 |  |
| *CaTIP41^*^*  *Ca_05619* | CaTIP41q(F) | GTTGTACTTCGGGAGAGTTGCT | 60 | 115 |
|  | CaTIP41q(R) | GGAGCTTCTGGCTTATGATGCT | 60 |  |
| *CaCAC^*^*  *Ca_02271* | CaCACq(F) | CATGGACTAGACCACCAATTCA | 60 | 110 |
|  | CaCACq(R) | AACAGTGTTGTACCCGCTCTTT | 60 |  |
| *LcFITNESS*  *Lcu.2RBY.5g049150* | LcFITNESSq(F) | CCACAAAACTCTCGTGATAGTAGC | 60 | 115 |
|  | LcFITNESSq(R) | TCACCCCAAAATTGCATTTGCT | 60 |  |
| *LcFHY3*  *Lcu.2RBY.2g095080* | LcFHY3q(F) | ACCGTTGGTGCTTTTCGTTG | 60 | 141 |
|  | LcFHY3q(R) | CGTGGAGCATGTTCTCCCAT | 60 |  |
| *LcFAR1*  *Lcu.2RBY.5g061340* | LcFAR1q(F) | TTCAGCTTGAGGATGCCCAG | 60 | 86 |
|  | LcFAR1q(R) | TGATCGTAGTACAGCCCCCA | 60 |  |
| *LcDOF4.2*  *Lcu.2RBY.2g004530* | LcDOF4.2.2q(F) | AGCTCATCCTCAGCCTCAGA | 60 | 136 |
|  | LcDOF4.2.2q(R) | TGTTCCTCCCTGAGTCCAGT | 60 |  |
| *LcBS1*  *Lcu.2RBY.1g038880* | LcBS1.2q(F) | CTTCCAGTGAAAGTCCATGCC | 60 | 80 |
|  | LcBS1.2q(R) | AGAGGCAGTCTAGTCTGTGGT | 60 |  |
| *LcRPL2^*^*  *Lcu.2RBY.5g066210* | LcRPL2q(F) | ATTGAGCTGAACCCAGGACAA | 60 | 80 |
|  | LcRPL2q(R) | GACGTGGGCTCCTTCAAGATC | 60 |  |
| *LcTUB^*^*  *Lcu.2RBY.6g047660* | LcTubq(F) | CACCCAGCAAATGTGGGATT | 60 | 80 |
|  | LcTubq(R) | TGGCAGATGCGGTGAGGTA | 60 |  |
| *LcRBC1^*^*  *Lcu.2RBY.L006960*  *Lcu.2RBY.7g020650*  *Lcu.2RBY.7g020530* | LcRBC1q(F) | ATGCCTGCTCTGACCGAGAT | 60 | 80 |
|  | LcRBC1q(R) | TTTCCCCAAGGGTGTCCTAAA | 60 |  |

* Reference genes for expression normalization in the chickpea (Reddy et al. 2016) and lentil (Sinha et al. 2019).

**Table S2.** Summary of RNA libraries, Illumina sequencing raw data, filtered reads, and mapped reads for the samples of chickpea and lentil.

| **Chickpea** | | | | **Raw** | **Filtered** | | **Mapped** | | | | | |
| --- | --- | --- | --- | --- | --- | --- | --- | --- | --- | --- | --- | --- |
| **Cultivar** | **Organ** | **Samples** | **ID** | **Raw Read Pairs** | **Both Surviving** | **% surviving** | **aln_conc=1** | **aln_conc>1** | **aln_disc=1** | **aln_unpair=1** | **aln_unpair>1** | **overall_aln%** |
| Blanco lechoso | Axillary bud | BXT-1-1_S29 | BX1 | 9,290,503 | 8,695,024 | **93.59%** | 7,824,446 | 338,327 | 258,342 | 204,225 | 26,736 | **98.18%** |
|  |  | BXT-1-2_S30 | BX2 | 14,559,735 | 13,260,289 | **91.08%** | 11,949,956 | 618,387 | 316,579 | 313,531 | 42,939 | **98.51%** |
|  |  | BXT-1-3_S31 | BX3 | 14,065,466 | 12,769,683 | **90.79%** | 11,089,311 | 513,011 | 614,716 | 464,746 | 72,185 | **97.77%** |
|  | Apical bud | BAT-1-1_S26 | BA1 | 11,995,947 | 11,195,847 | **93.33%** | 10,101,648 | 461,184 | 300,051 | 223,960 | 28,746 | **98.15%** |
|  |  | BAT-1-2_S27 | BA2 | 3,374,557 | 3,091,832 | **91.62%** | 2,721,092 | 138,413 | 125,349 | 107,733 | 17,697 | **98.57%** |
|  |  | BAT-1-3_S28 | BA3 | 11,619,208 | 10,688,497 | **91.99%** | 9,149,930 | 396,339 | 605,067 | 451,284 | 64,986 | **97.39%** |
| FLIP07-318C | Axillary bud | FXT-1-2_S15 | FX1 | 8,610,267 | 8,038,413 | **93.36%** | 6,952,041 | 342,668 | 389,380 | 267,357 | 48,405 | **97.56%** |
|  |  | FXT-1-3_S16 | FX2 | 9,905,376 | 9,078,856 | **91.66%** | 7,962,254 | 359,884 | 392,661 | 283,762 | 44,217 | **97.80%** |
|  | Apical bud | FAT-1-1_S17 | FA1 | 11,137,712 | 10,306,301 | **92.54%** | 9,327,013 | 422,677 | 252,091 | 191,432 | 24,505 | **98.09%** |
|  |  | FAT-1-3_S19 | FA2 | 14,527,599 | 13,316,821 | **91.67%** | 11,429,089 | 519,738 | 746,574 | 515,757 | 80,917 | **97.57%** |
|  |  | IAT-1-2_S9 | FA3 | 14,222,929 | 13,021,492 | **91.55%** | 11,208,223 | 539,844 | 744,759 | 558,725 | 99,242 | **98.47%** |

| **Lentil** | | | | **Raw** | **Filtered** | | **Mapped** | | | | | |
| --- | --- | --- | --- | --- | --- | --- | --- | --- | --- | --- | --- | --- |
| **Cultivar** | **Organ** | **Samples** | **ID** | **Raw read pairs** | **Both surviving** | **% surviving** | **aln_conc=1** | **aln_conc>1** | **aln_disc=1** | **aln_unpair=1** | **aln_unpair>1** | **overall_aln%** |
| Campisi | Apical bud | IAT-1-1_S8 | CmA1 | 9,163,671 | 8,412,852 | **91.81%** | 6,762,658 | 332,584 | 465,117 | 554,990 | 85,920 | **93.68%** |
|  |  | IAT-1-3_S10 | CmA2 | 11,855,107 | 10,907,893 | **92.01%** | 9,097,118 | 424,566 | 416,811 | 508,846 | 72,427 | **93.78%** |
|  |  | FAT-1-2_S18 | CmA3 | 5,862,483 | 5,492,123 | **93.68%** | 4,699,721 | 221,189 | 149,545 | 249,602 | 36,708 | **94.93%** |
|  | Axillary bud | IXT-1-1_S11 | CmX1 | 11,466,506 | 10,737,152 | **93.64%** | 9,181,780 | 415,387 | 310,755 | 499,849 | 68,673 | **94.92%** |
|  |  | IXT-1-2_S12 | CmX2 | 11,891,143 | 10,877,887 | **91.48%** | 9,358,322 | 456,298 | 292,183 | 534,668 | 78,473 | **95.73%** |
|  |  | IXT-1-3_S13 | CmX3 | 13,481,417 | 12,410,646 | **92.06%** | 10,483,648 | 458,381 | 389,580 | 570,424 | 82,342 | **93.94%** |
| Castellana | Apical bud | CAT-1-1_S23 | CsA1 | 9,542,567 | 8,829,671 | **92.53%** | 7,582,295 | 350,613 | 328,729 | 354,742 | 55,279 | **95.89%** |
|  |  | CAT-1-2_S24 | CsA2 | 9,328,787 | 8,466,644 | **90.76%** | 7,260,806 | 310,820 | 333,043 | 452,691 | 69,011 | **96.44%** |
|  |  | CAT-1-3_S25 | CsA3 | 7,907,636 | 7,301,786 | **92.34%** | 6,128,019 | 280,295 | 353,398 | 359,147 | 59,711 | **95.47%** |
|  | Axillary bud | CXT-1-1_S20 | CsX1 | 13,210,248 | 12,136,882 | **91.87%** | 10,295,856 | 433,643 | 535,169 | 581,922 | 86,969 | **95.57%** |
|  |  | CXT-1-2_S21 | CsX2 | 12,628,642 | 11,671,624 | **92.42%** | 9,919,257 | 463,587 | 514,089 | 639,320 | 106,869 | **96.56%** |
|  |  | CXT-1-3_S22 | CsX3 | 8,441,207 | 7,936,037 | **94.02%** | 6,876,258 | 285,165 | 264,558 | 326,680 | 48,288 | **95.94%** |

Legend: aln_conc=1: paired reads aligned concordantly exactly 1 time; aln_conc>1: paired reads aligned concordantly >1 time; aln_disc=1: paired reads aligned discordantly 1 time; aln_unpair=1: one of the pair aligned exactly 1 time; aln_unpair>1: one of the pair aligned >1 time; overall_aln%: overall alignment rate; total: total mapped reads; unique: uniquely mapped reads; ambiguous: ambiguously mapped reads.

**Table S3.** Validation of relative expression level obtained from RNA-seq compared with real-time RT-PCR in chickpea and lentil datasets. BX: Blanco lechoso axillary bud, BA: Blanco lechoso apical bud, FX: FLIP07-318C axillary bud, FA: FLIP07-318C apical bud, CsX: Castellana axillary bud, CsA: Castellana apical bud, CmX: Campisi axillary bud, and CmA: Campisi apical bud. DET: differentially expressed transcript.

| **Chickpea** | | | | | | | | | | **Pearson correlation coefficient and *p*-values (between parenthesis) between RNA-seq and real-time RT-PCR data^!^** |
| --- | --- | --- | --- | --- | --- | --- | --- | --- | --- | --- |
| **Gene** | **Contrast** | **Transcript ID** | **RNA-seq^*^** | | | | **RT-PCR^#^** | | |  |
|  |  |  | **logFC** | ***p*-value** | **FDR** | **DET** | **logFC** | **Tukey^$^** | **DET**^&^ |  |
| *CaBES1* | BX_BA | XM_004500981.3 | 0.043468928 | 0.824871882 | 0.981226986 | ns | 0.00512 | ns | ns | 0.29214 (2.6378E-10) |
|  | FX_FA | XM_004500981.3 | -0.422357212 | 0.055448685 | 0.09353888 | ns | -0.03878 | ns | ns |  |
|  | FX_BX | XM_004500981.3 | -0.329837169 | 0.134213074 | 0.22466613 | ns | -0.01760 | ns | ns |  |
|  | FA_BA | XM_004500981.3 | -0.329837169 | 0.134213074 | 0.22466613 | ns | 0.02630 | ns | ns |  |
| *CaFHY3* | BX_BA | XM_004486890.3 | 0.171544775 | 0.389157105 | 0.920001052 | ns | -0.01109 | ns | ns | 0.13712 (2.63156E-10) |
|  | FX_FA | XM_004486890.3 | -0.284373695 | 0.197635262 | 0.275623356 | ns | 0.00131 | ns | ns |  |
|  | FX_BX | XM_004486890.3 | -0.290236088 | 0.189458063 | 0.295370426 | ns | 0.03153 | ns | ns |  |
|  | FA_BA | XM_004486890.3 | 0.165682382 | 0.404424959 | 0.644638826 | ns | 0.01913 | ns | ns |  |
| *CaFAR1* | BX_BA | XM_004491110.3 | 0.142281469 | 0.350950715 | 0.90691287 | ns | -0.00435 | ns | ns | 0.82841 (1.10875E-09) |
|  | FX_FA | XM_004491110.3 | 0.25699205 | 0.124377001 | 0.186885153 | ns | 0.02834 | ns | ns |  |
|  | FX_BX | XM_004491110.3 | 0.145800713 | 0.384173236 | 0.503559428 | ns | 0.06589 | * | yes |  |
|  | FA_BA | XM_004491110.3 | 0.031090132 | 0.838481121 | 0.926196774 | ns | 0.03320 | ns | ns |  |
| *CaDOF4.2* | BX_BA | XM_004485743.3 | -0.03530612 | 0.91155585 | 0.992482668 | ns | 0.00000 | ns | ns | 0.72041 (5.37540E-07) |
|  | FX_FA | XM_004485743.3 | -0.083061267 | 0.824046795 | 0.866704331 | ns | -0.00001 | ns | ns |  |
|  | FX_BX | XM_004485743.3 | -0.67204488 | 0.068344309 | 0.130930037 | ns | 0.00001 | ns | ns |  |
|  | FA_BA | XM_004485743.3 | -0.624289732 | 0.055261409 | 0.198387074 | ns | 0.00001 | ns | ns |  |
| *CaFHY1* | BX_BA | XM_004514334.3 | 0.062415266 | 0.780195567 | 0.973828867 | ns | 0.01901 | ns | ns | 0.77131 (0.00003) |
|  | FX_FA | XM_004514334.3 | 1.062159409 | 9.02123E-06 | 5.3046E-05 | yes | 0.21231 | * | yes |  |
|  | FX_BX | XM_004514334.3 | 0.883979521 | 0.000218199 | 0.001070369 | yes | 0.12052 | * | yes |  |
|  | FA_BA | XM_004514334.3 | -0.115764622 | 0.605358706 | 0.793989388 | ns | -0.07277 | * | yes |  |
| Percentage of agreement between RNA-seq and real-time RT-PCR data^@^ | | | | | | | | | 90% |  |

| **Lentil** | | | | | | | | | | | **Pearson correlation coefficient and *p*-values (between parenthesis) between RNA-seq and real-time RT-PCR data^!^** |
| --- | --- | --- | --- | --- | --- | --- | --- | --- | --- | --- | --- |
| **Gene** | **Contrast** | **Transcript ID** | **RNA-seq^*^** | | | | **RT-PCR^#^** | | | |  |
|  |  |  | **logFC** | ***p*-value** | **FDR** | **DET** | **logFC** | **Tukey^$^** | **DET**^&^ | |  |
| *LcFITNESS* | CmX_CmA | Lcu.2RBY.5g049150.1 | 1.001258188 | 0.019051775 | 0.758161221 | ns | 0.02352 | ns | ns | | 0.21896 (0.00004) |
|  | CsX_CsA | Lcu.2RBY.5g049150.1 | 0.002725618 | 0.994988068 | 1 | ns | 0.09285 | * | yes | |  |
|  | CsX_CmX | Lcu.2RBY.5g049150.1 | -1.153217899 | 0.006663955 | 0.055326719 | ns | 0.00994 | ns | ns | |  |
|  | CsA_CmA | Lcu.2RBY.5g049150.1 | -0.154685329 | 0.722269469 | 0.814801494 | ns | -0.05940 | ns | ns | |  |
| *LcFHY3* | CmX_CmA | Lcu.2RBY.2g095080.1 | 0.141792445 | 0.550419757 | 1 | ns | 0.000004 | ns | ns | | 0.35172 (2.02790E-09) |
|  | CsX_CsA | Lcu.2RBY.2g095080.1 | -0.19307475 | 0.415062917 | 0.565042698 | ns | 0.000006 | ns | ns | |  |
|  | CsX_CmX | Lcu.2RBY.2g095080.1 | -0.162691596 | 0.490831877 | 0.794068735 | ns | 0.000005 | ns | ns | |  |
|  | CsA_CmA | Lcu.2RBY.2g095080.1 | 0.172175598 | 0.469815759 | 0.609630429 | ns | 0.000003 | ns | ns | |  |
| *LcFAR1* | CmX_CmA | Lcu.2RBY.5g061340.1 | -0.270830566 | 0.404031172 | 1 | ns | -0.00001 | ns | ns | | 0.95901 (0.000008) |
|  | CsX_CsA | Lcu.2RBY.5g061340.1 | -0.619958195 | 0.05498555 | 0.130082446 | ns | 0.00000 | ns | ns | |  |
|  | CsX_CmX | Lcu.2RBY.5g061340.1 | 0.149855957 | 0.642999018 | 0.877166733 | ns | 0.00000 | ns | ns | |  |
|  | CsA_CmA | Lcu.2RBY.5g061340.1 | 0.498983585 | 0.123309572 | 0.232714119 | ns | -0.00002 | ns | ns | |  |
| *LcDOF4.2* | CmX_CmA | Lcu.2RBY.2g004530.1 | 0.105298052 | 0.817613296 | 1 | ns | -0.00038 | ns | ns | | 0.04548 (2.64441E-06) |
|  | CsX_CsA | Lcu.2RBY.2g004530.1 | 1.29622947 | 0.006570057 | 0.026218185 | yes | 0.00003 | ns | ns | |  |
|  | CsX_CmX | Lcu.2RBY.2g004530.1 | 0.354011809 | 0.412457575 | 0.744729343 | ns | -0.00002 | ns | ns | |  |
|  | CsA_CmA | Lcu.2RBY.2g004530.1 | -0.83691961 | 0.095257985 | 0.192180284 | ns | -0.00043 | ns | ns | |  |
| *LcBS1* | CmX_CmA | Lcu.2RBY.1g038880.1 | -0.805705387 | 0.004054867 | 0.372167113 | ns | -0.00381 | ns | ns | | 0.54517 (5.9404E-06) |
|  | CsX_CsA | Lcu.2RBY.1g038880.1 | -0.548122087 | 0.050964296 | 0.12295919 | ns | 0.00504 | ns | ns | |  |
|  | CsX_CmX | Lcu.2RBY.1g038880.1 | -0.345167122 | 0.217940141 | 0.55475691 | ns | 0.00292 | ns | ns | |  |
|  | CsA_CmA | Lcu.2RBY.1g038880.1 | -0.602750422 | 0.031260133 | 0.084956508 | ns | -0.00593 | ns | ns | |  |
| Percentage of agreement between RNA-seq and real-time RT-PCR data^@^ | | | | | | | | | | 90% |  |

* The RNA-seq data related to differential expression transcripts (DETs) were presented as log(fold change) (LogFC), supported by FDR <0.05. The monitored DETs were asked whether or not they are differentially expressed by both RNA-seq: “yes” indicates transcript differentially expressed, while “ns” indicates transcript no differentially expressed when treatments were contrasted.

^#^ For relative gene expression by real-time RT-PCR, the cycle threshold (Ct) values obtained for each DET and endogenous references (Table S1) were used to calculate first the average of the technical triplicates, then, the average Ct values of each biological replicate were transformed to relative expression using the 2^-∆Ct formula (Figures S5 and S6). Subsequently, the ∆Ct value of each biological replicate was used to calculate the average of the biological triplicate for each treatment. Then, these average ∆Ct values of each treatment were transformed to fold change (FC) using the 2^-∆∆Ct formula contrasting the suitable treatments, and, finally, these fold change values were transformed to LogFC using the Log(FC) formula.

**^$^** The relative expression values obtained using real-time RT-PCR for each transcript, in each treatment, with three biological replicates each, were evaluated for statistical differences according to Tukey’s test at a 95% significance level. The asterisk indicates differences in the relative expression with statistical support between contrasted treatments, while “ns” indicates non-statistically significant differences.

^&^ The monitored DETs by real-time RT-PCR were asked whether or not they are differentially expressed with statistical support, and the answer was "ns" (for non-significant) or "yes" (when DET).

^@^ For the percentage of agreement between RNA-seq and real-time RT-PCR data, 20 comparisons for chickpea and lentil were performed corresponding to five DETs and four contrasts each, and the accordance was calculated based on number of contrasts with DET or non-DET supported by statistical equality between RNA-seq and real-time RT-PCR data *versus* the number of contrasts non-equality between RNA-seq and real-time RT-PCR data.

^!^ In the Pearson correlation coefficient analysis, RNA-seq values corresponded to TPM (transcripts per million) in each library or sample, while real-time RT-PCR values corresponded to 2^-∆Ct in each library or sample.

**File S1.** Gene ID, gene category, description, log(fold change) for differentially expressed transcripts in the pairwise comparison for both chickpea genotypes and tissues, *P*-value, and FDR values (excel file).

**File S2.** Gene ID, gene category, description, log(fold change) for differentially expressed transcripts in the pairwise comparison for both lentil genotypes and tissues, *P*-value, and FDR values (excel file).

**File S3.** Significantly enriched GO terms of differentially expressed transcripts between two different chickpea and lentil genotypes and tissues. Only differentially expressed transcripts with FDR < 0.05 and log(fold change) lower than -2 or greater than 2 were used (excel file).

**File S4.** Plant hormone pathways that were impacted by the differentially expressed transcripts identified in chickpea and lentil by RNA-seq. These data were generated by using MapMan software (excel file).
